# Optimal AGC regulators for power systems under restructured configuration with hybrid ESS

**DOI:** 10.1038/s41598-025-19710-3

**Published:** 2025-10-14

**Authors:** Ram Naresh Mishra, Narendra Kumar, Mohd Zuhaib, Marwan Ahmad Abdullah Sufyan

**Affiliations:** 1 Department of Electrical Engineering, GLA University, Mathura, UP 281406 India; 2 Department of Electrical Engineering, Delhi Technological University, Delhi, 110042 India; 3 College of Engineering and Information Technology, Aljanad University of Science and Technology, Taiz, Yemen

**Keywords:** Automatic generation control, FOPID controller, Polar fuzzy controller, (1 + FOID) controller, Electric vehicle, PII^ʎ^ DD^µ^ controller, Ultra-capacitor, Energy science and technology, Engineering, Mathematics and computing

## Abstract

Several new rivals are entering the market as a result of the global restructuring of the electric power sector, which is also intensifying competition among existing market players. Better services, greater choices, and less expensive power are all intended to benefit consumers. For such systems to be more reliable, new control techniques with hybrid ESS are crucial. The CSOA-based hybrid ESS for a restructured T-G-S power system combines a polar fuzzy controller (PFC) with an FOPID controller. Testing is done on this controller’s resilience with CTD, random load demand, and parameter uncertainty. Additionally, a reconstructed N-G-S system is used to assess the viability of the suggested controller. A CSOA-based combined FOPID controller and polar fuzzy controller using hybrid ESS can improve frequency responses for Area-1 and Area-2 by 15.59% and 21.83%, respectively, and enhance the response of power variation of the tie-line under contract violation by 27.16% when compared to the CSOA: PII^ʎ^DD^µ^ controller. The effectiveness of a CSOA-based combination (1 + FOID) controller and a polar fuzzy controller for a restructured T-H-G power system is demonstrated in another study. Other controllers, including GA: 1 + PFC, SOA: FOPID, SOA: PID, GA: PID, and OARs, are used to verify its performance. These controllers include a variety of linkages, including a control variable ∆P_dc_ with the turbine’s controller. The proposed control scheme has the lowest cost function among various control strategies and has been shown to outperform several existing control methods. Moreover, it offers incredibly dependable performance under different load conditions.

**Supplementary Information:**

The online version contains supplementary material available at 10.1038/s41598-025-19710-3.

## Introduction

### Literature review

The developments based on cutting-edge communication innovations include the introduction of green sources of energy, implementation of innovative concepts such as the smart grid, and the automation of electric system regulators. These changes all add to the complexity of electrical systems^1,2^. A consistent influence is exerted by the aforementioned elements on the safety, steadiness, and operation of the power facilities. A great deal of attention has been paid to the management of frequency in PS because of the significance of this topic^3^.The amount of intrusion of renewable energy sources (RES) into power networks, such as wind farms and solar plants, causes frequency changes, which in turn leads to a rise in the level of uncertainty associated with active power generation. This leads to fluctuate in frequency rapidly, which would be a consequence of the stochastic character of demand. Because of this, the employment of LFC approaches that are more dependable and optimal is essential to handle such challenges. Non-integer values define the ordering of the integral and derivative operators in the FO controller architecture, providing greater flexibility in device design. Oustaloup^4^ provided the first suggestion for the use of FO operators in control. Therefore, researchers have used a great number of FO controllers that are based on optimization approaches in order to solve LFC difficulties. This is due to the fact that FO controllers have benefits over the IO-based conventional controller^3^. Alomoush conducted a performance evaluation of using FOPID and IOPID controllers in dual-area electrical systems^5^. Reference^6^ analyses the LFC of an isolated system with a BB-BC-based FOPID controller. Within the scope of the study^7^, isolated multi-source PS that makes use of a FOPID controller for LFC In the event of parameter ambiguity. In addition, the influence of SMES on two agent reconstructed PS was explored in^8^, which used bat optimization tailored FOPID controller information. Higher-order power systems have used FOPID based on the GOA for LFC^9^. A dual-area RPS is suggested to use a FOPI controller for frequency management^10^. A FOPID controller is used in a revolutionary reduced order LFC approach described in^11^. The frequency and tie-line of the multi-source reconstructed power network were stabilized by the use of a FOIDF controller. The system is compared using a tweaked IO-based PIDF controller^12^. Al-Mayyahi et al.^13^ suggested the Bat optimization based FOPID controller for monitoring the circular motion. In another research, Zhang et al.^14^ presented WOA based FOPID control. Many control techniques based on FO controllers have been developed and used in various contexts to provide adequate control. EVs’ involvement in deregulated LFC environment in conjunction with other traditional energy sources such as gas turbines, thermal, and hydropower plants. Lurie^15^ presented the TID controller first. It replaces the proportional controller with a tilted PID parameter having a TF of s^-1/n^, closely connected to the FOPID. TID controllers may reject disturbances better than PID controllers and reduce system parameter changes in closed-loop systems. Several studies have demonstrated the supremacy of TID controller over PID controller in increasing the speed of the controller response and system stability^15–17^. A FOTID controller tuned using the SSA was developed by Sharma et al.^18^ for frequency settlement of hybrid power system. Additionally, a comparison was made between the error indices and transient response of the suggested controllers based on the SSA and GWO algorithms, and the controllers that are already in use. This was done to determine which controllers were superior. As a result, the simulations in that paper indicate the appropriateness of the FOTID controller.

For LFC uses, Sharma et al.^19^ introduced a dual-stage regulator. The controller comprises IOPD controllers and FOTID controllers. However, Lu et al.^20^ suggested a methodical tuning strategy for the FOTID controller for high-order and first-order processes with time delay. The study provided a detailed description of the design process and related procedures for the robust TID controller. Ultimately, it was evident from the simulation results that the suggested controller outperformed the PID, FOPI, and FOPID controllers in terms of resilience and transient performance. Moreover, this study utilized hybrid controller i.e. the functionality of TID and FOPID controllers. Mohamed et al.^21^ created a hybrid controller for load frequency management that combines FOPID and TID controllers. The ISE criteria was used to optimize the six distinct tuneable parameters in the developed controller utilizing the Manta Ray Foraging (MRF) method. That article looked at the developed controller’s resilience in the face of load disruptions and system parameter variations. Ahmet et al.^22^ suggested a hybrid FOPID-TID controllers for load frequency and EV management. Furthermore, the article used the AEO technique to find the hybrid controller’s ideal settings that performs much better, more robustly, and steadily throughout a broad range of quick reactions during transients and parameter uncertainty. Chaudhary et al.^23^ discussed combined FOPI-FOPTID controller based on ACO and GNA to meet AGC objectives effectively as compared to PID, FOPID, and FOPI-FOPID controllers. Yanmaz et al.^24^ suggested another hybrid controller based on FOPTID to regulate a static compensation system efficiently. The Pathfinder Optimization Algorithm (POA) was used in that study to optimize three different types of model predictive controllers. Empirical findings have shown the efficacy of the offered FOPTID-MPC controller.

Polar fuzzy logic controllers address FLC’s disadvantages. Linguistic values fluctuate with θ, the unit circle angle, and membership values are on µ(θ). Polar fuzzy is beneficial when polar coordinates are natural or a cyclic variable. Polar fuzzy sets quantify truth-valued linguistic variables. The sole difference between polar and regular fuzzy sets is Polar fuzzy sets are specified by angle and repeat forms every 2π radian. The researchers have shown their interest to advocate the performance analysis of PFC for LFC^25,26^. Lofty et.al designed PFC for hybrid power system with V2GT^27^. Mishra et.al. described PFC considering various membership functions for multi-source RPS^28,29^. Different research attempts to compare the effectiveness of various hybrid ESSs for the AGC of a deregulated THG power system. The article proposes and compares an optimum controller with a COC-FOD controller to enhance AGC performance. The COC-FOD controller demonstrated its robustness and effectiveness by offering acceptable, stable performance during significant fluctuations in the system parameters^30^. This study describes the utilization of hybrid ESS with unique AGC regulators for multi-source restructured power systems.

### The gap in research and motivation

The FOPID controller extends the classical PID by introducing fractional differ-integral orders, which provide additional tuning flexibility. This leads to improved frequency regulation by offering finer control over the dynamic response. FOPID has been shown to achieve lower overshoot, faster settling time, and enhanced disturbance rejection compared to classical PID, making it particularly suitable for AGC under uncertain and varying load conditions. The polar fuzzy controller (PFC) further enhances AGC performance by embedding fuzzy logic in polar coordinates (magnitude and angle). Unlike conventional fuzzy controllers, which may require a large rule base, the polar representation simplifies rule formulation and improves adaptability across a wide operating range. PFC is highly effective in handling nonlinearities, model uncertainties, and noisy measurements, while providing robust performance, smooth control signals, and easy scalability to multi-area interconnected systems.

The integration of FOPID and PFC as integrated concepts is still not well explored, even though the study of activities about the FOPID controller^5–9,11,13,14,24^ and polar fuzzy controllers^25,26,28–30^ is done individually. Thus, in the AGC of the restructured T-G-S power system, CSOA based combined FOPID and PFC (gbellmf) controller is implemented. The proposed strategy aims to achieve superior dynamic performance, robustness against parameter variations, communication delays, and renewable-induced uncertainties, and adaptability for both small-scale micro grids and large power systems in a restructured market environment. Thus, the investigation the authors have already conducted in^5–9,11,13,14,24–26,28,30^ is expanded upon in this work. The study’s authors^5^ have independently implemented the FOPID and IOPID controllers. Here, the authors used a PII^ʎ^DD^µ^ controller, a combination of FOPID and IOPID controllers. The researchers discuss the FOPI^10^ and FOIDF^12^. This study’s authors used a combined (1 + FOID) and PFC (gbellmf) controller, which performed better than the individual controllers for the restructured T-H-G system. The majority of writers have employed ESS in their research. However, researchers have not yet made extensive use of hybrid ESS. However,^20^ reports on studies on hybrid ESS, such as SMES + RFB, FES + RFB, and UC + RFB. This study makes use of hybrid energy storage systems (ESS), which combine electric vehicles (EVs) and ultra-capacitors (UCs). The investigations were motivated by the deficiencies mentioned above to enhance the dynamic responsiveness of the suggested restructured systems.

### Contribution

The contribution discussed in this article can be summed up as follows:

1. To test the feasibility of CSOA-optimized combined FOPID and PFC with hybrid ESS for a reorganized T-G-S system. Its performance is compared with CSOA-FOPID + PFC, CSOA-FOPID, CSOA-PIDD, CSOA-PID, and GA-PID controllers.
2. The resilience of the controller (a) is evaluated under RLD, parameter uncertainty, and CTD. Furthermore, the viability of the recommended controller is verified in a restructured N-G-S system.
3. To test the viability of CSOA tuned PII^ʎ^DD^µ^ controller for a reorganized T-G-S system utilizing hybrid ESS. Its performance is compared with CSOA: PIILDDM, CSOA: PIILD, CSOA: PID, and GA: PID.
4. To assess the flexibility and reliability of CSOA: FOPID + PFC and CSOA: PII^ʎ^DD^µ^ controllers with hybrid ESS during random load disturbances (RLD) and discuss the superiority of these controllers.
5. To explore the efficacy of CSOA-optimized combined (1 + FOID) controller and polar fuzzy controller (PFC) for the restructured T-H-G system. Its performance is tested with other controllers such as GA: 1 + PFC (gbellmf), SOA: FOPID, SOA: PID, GA: PID, and OARs with various interconnections that include control variable ∆P_dc_ with controller of the turbine.

### Outline of the research article

The research study is structured as follows: The second section examines the recommended systems. Section “The controller’s architecture” presents the design of the proposed controllers, along with a detailed explanation and a flow chart of CSOA, SOA, and GA. Section “The hybrid ESS” covers the hybrid ESS. Section “Simulation outcomes and discussion” presents the simulation results and provides an explanation of them. Section “Risks to the validity of the findings” addresses threats to the validity of the findings. Portion 7 represents the article’s last portion.

## Restructured systems under investigation

**Fig. 1:**
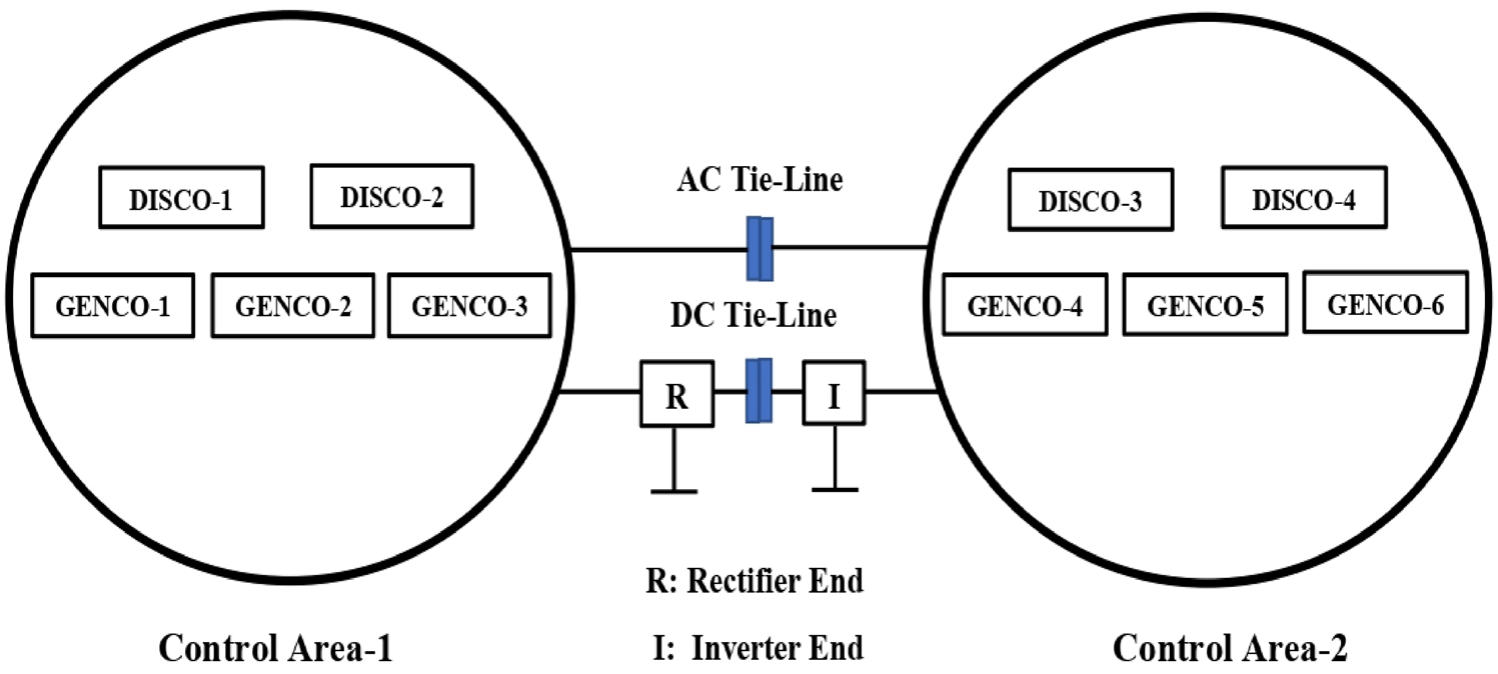
Block diagram of proposed system.

**Fig. 2:**
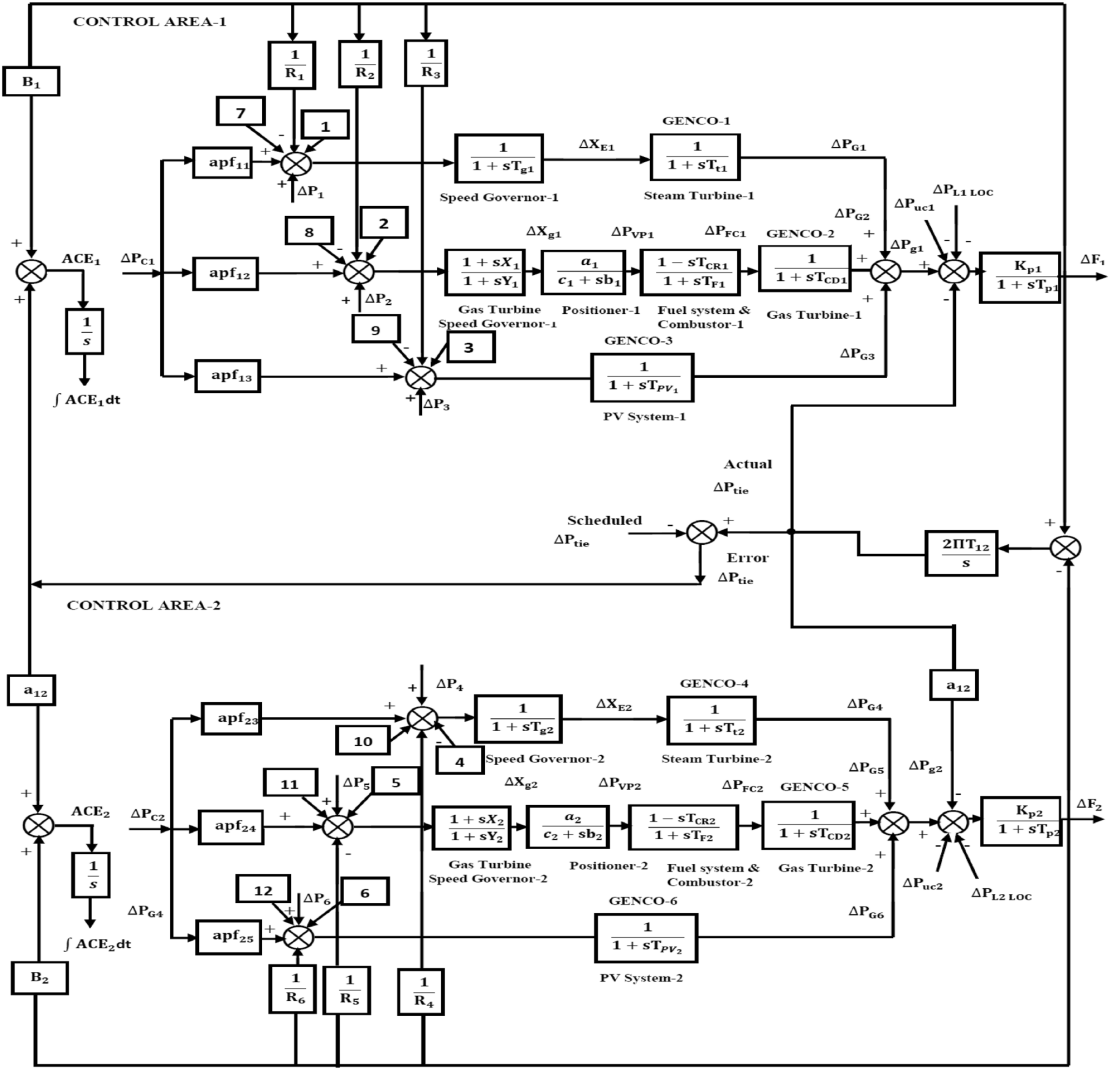
The TF model of restructured T-G-S system.

**Fig. 3:**
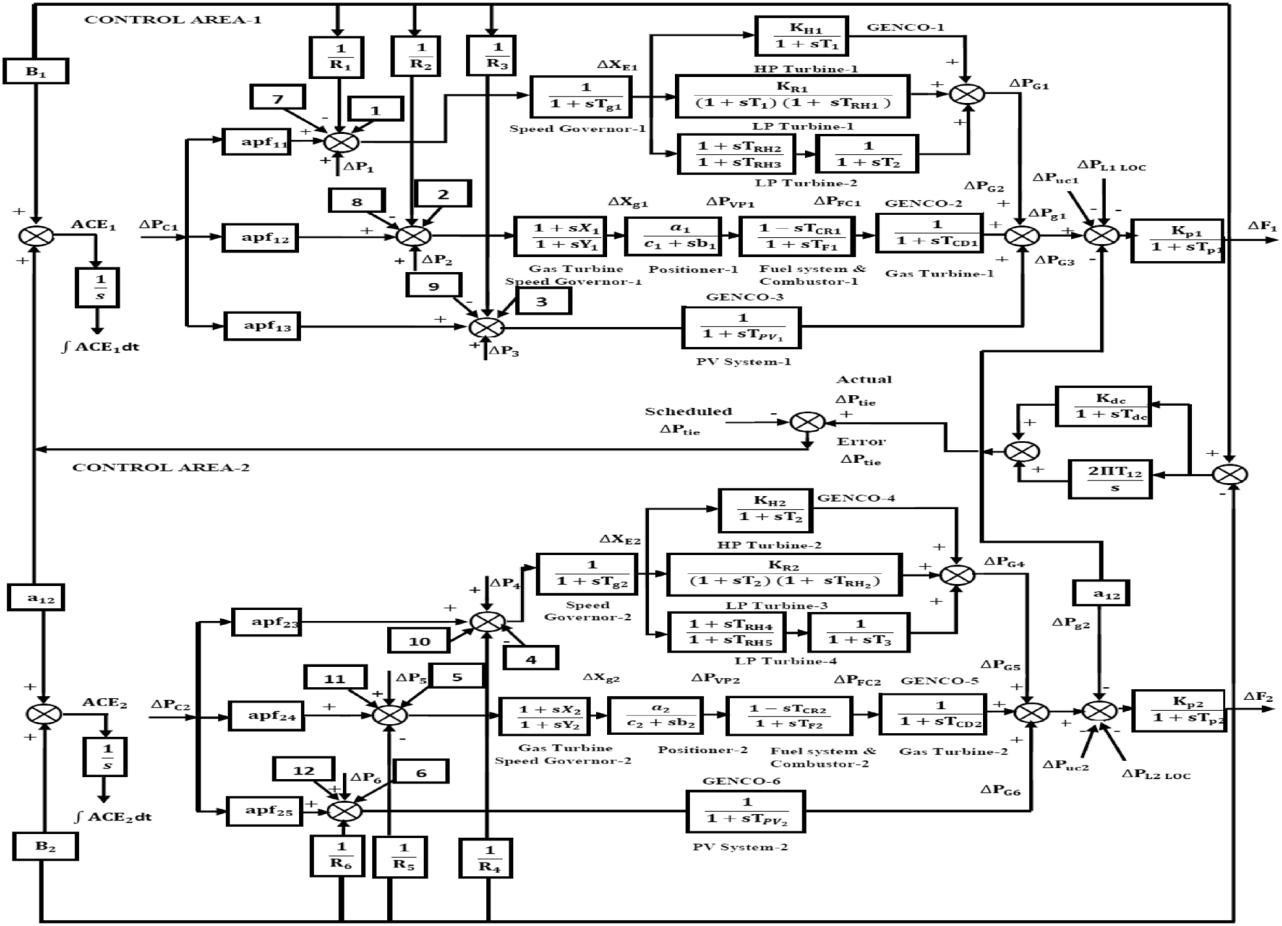
The TF model of restructured N-G-S system.

**Fig. 4:**
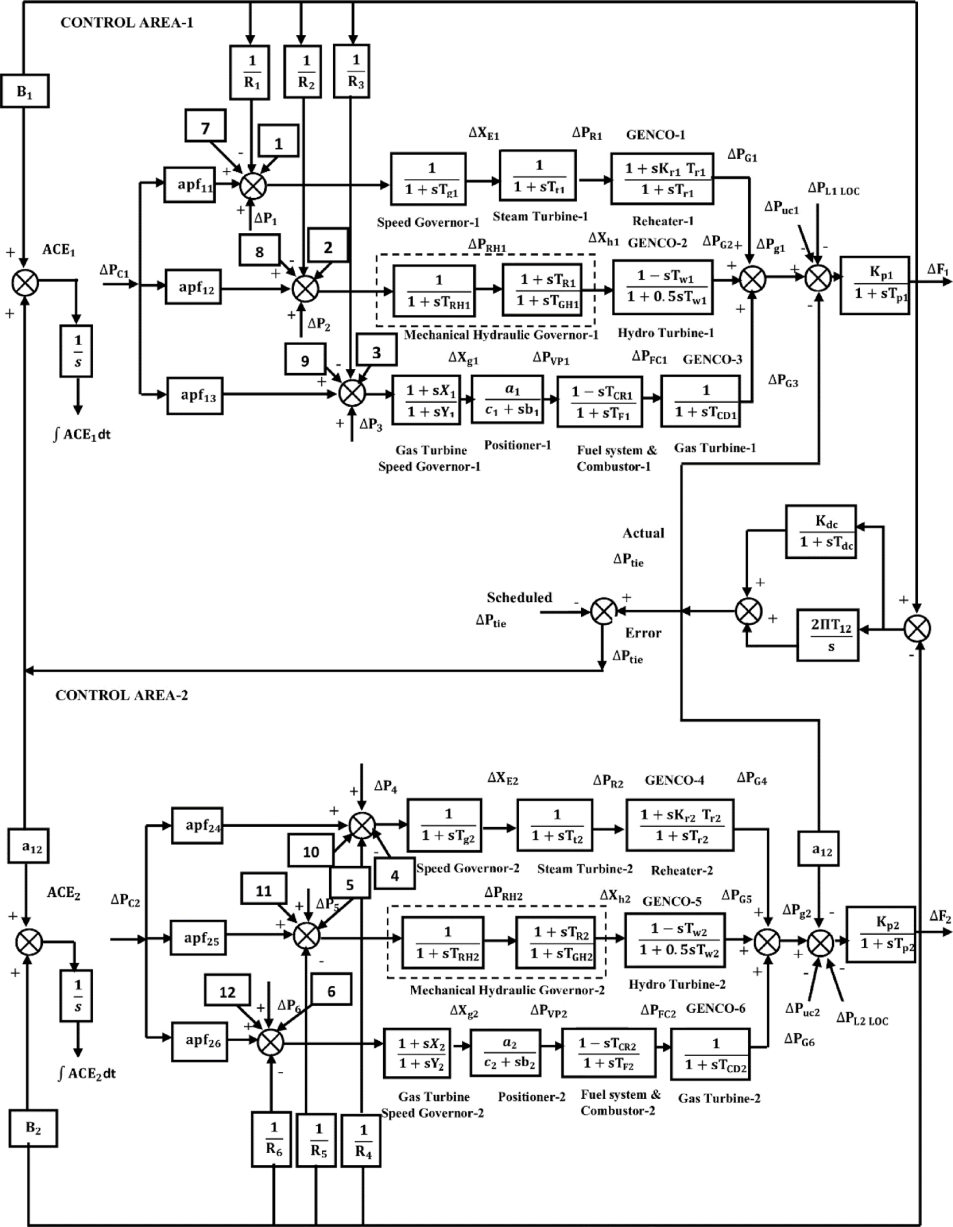
The TF model of restructured T-H-G system.

This article considers three different RPS. Each system comprises of two control regions that are linked by parallel tie-lines. Figure 1 shows that each control area has two DISCOs and three GENCOs. Figure 2 depicts a restructured T-G-S system, with each region consisting of a non-reheat thermal power plant, a gas turbine system, and a solar PV system with two DISCOs. Figure 3 depicts a reconstructed N-G-S system comprising three generating businesses, each equipped with different types of nuclear, gas, and solar power facilities, and two distribution companies in each region. In the case of a restructured T-H-G system, as shown in Fig. 4, there are three generating firms with varying capacities: thermal, hydro, and gas units. The area participation factors for each system are apf_1_ = 0.6, apf_2_ = 0.2, and apf_3_ = 0.2. A DPM oversees DISCO-GENCO power exchanges.

Furthermore, the following parts offer a mathematical depiction of the system models:

### Non-reheat and reheat turbine model

The governor and the turbine of thermal power plant can be described by following equations.

$$G_{{governor}} = \frac{1}{{1 + sT_{g} }}$$

$$G_{{turbine}} = \frac{1}{{1 + sT_{t} }}$$

where, the time constants of the governor and the steam turbine are denoted by T_g_ and T_t_, respectively.

The following is the re-heater’s TF.

$$G_{{Tr}} (s) = \frac{{1 + sK_{r} T_{r} }}{{1 + sT_{r} }}$$

### Gas turbine model^27^

The gas turbine positioner and speed governor’s TFs are as follows:

$${G}_{Gg}\left(s\right)=\frac{1+\text{sX}}{1+\text{sY}}$$

$${G}_{Gp}\left(s\right)=\frac{a}{\text{c}+\text{sb}}$$

The TF for the combustor and fuel system is as follows:

$${G}_{Gf}\left(s\right)=\frac{1-{\text{sT}}_{\text{CR }}}{1+{\text{sT}}_{\text{F}}}$$

The gas turbine’s TF is as follows:

$${G}_{Gt}\left(s\right)=\frac{1}{{1+\text{sT}}_{\text{CD}}}$$

### Solar PV model

A linear TF can represent the photovoltaic power plant, as shown in the following equation. In this equation, the solar irradiance fluctuation is the input, and output is PV power^23,27^. Here, T_PV_ stands for the temporal constant associated with the PV model.

$$G_{{PV}} = \frac{1}{{1 + sT_{{PV}} }}$$

### Nuclear turbine model

The TF of the nuclear turbine.
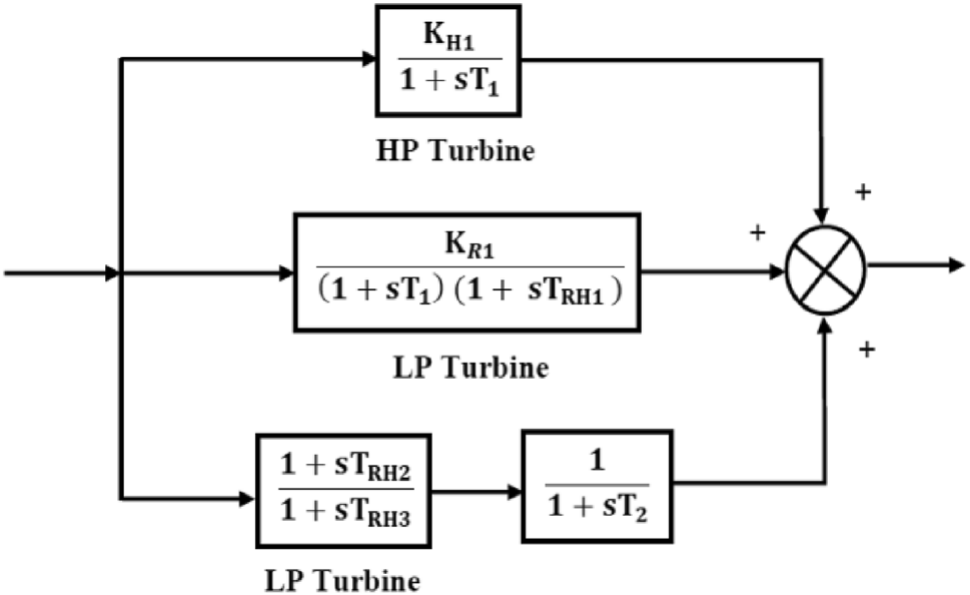


### Hydro turbine model

The hydraulic governor’s transfer function is listed below

$$G_{{Hg}} (s) = \left[ {\frac{1}{{1 + sT_{{RH}} }}} \right]\left[ {\frac{{1 + sT_{R} }}{{1 + sT_{{GH}} }}} \right]$$

The TF of hydro turbine.

$G_{Ht} (s) = \left[ {\frac{{1 - sT_{w} }}{{1 + 0.5sT_{w} }}} \right]$; where the time constant for hydraulic turbines is T_w_.

The following describes the power system’s TF.

$$Gps(s) = \frac{Kps}{{1 + sTps}}$$

This study uses a 6 × 4 DPM as follows. Where α denotes the contract participation factor (Cpf).

1 $$\left( {\begin{array}{*{20}c} {\alpha 11} & {\alpha 12} & {\alpha 13} & {\alpha 14} \\ {\alpha 21} & {\alpha 22} & {\alpha 23} & {\alpha 24} \\ {\alpha 31} & {\alpha 32} & {\alpha 33} & {\alpha 34} \\ {\alpha 41} & {\alpha 42} & {\alpha 43} & {\alpha 44} \\ {\alpha 51} & {\alpha 52} & {\alpha 53} & {\alpha 54} \\ {\alpha 61} & {\alpha 62} & {\alpha 63} & {\alpha 64} \\ \end{array} } \right)$$

The steady-state flow of an EHVAC tie-line connecting the system under analysis is as follows:

2 $$\Delta P_{{tie}}^{{actual}} = \frac{{2\pi T_{{12}} }}{S}(\Delta F_{1} - \Delta F_{2} )$$

This is how the system’s continuous tie-line electricity flow seems when the CCC mode is in use.

3 $$\Delta P_{{{\text{dc}}}} = \frac{{K_{{{\text{dc}}}} }}{{1 + sT_{{{\text{dc}}}} }}(\Delta F_{1} - \Delta F_{2} )$$

The following is the composition for parallel interties:

4 $$\Delta P_{{tie}}^{{Actual}} = \Delta P_{{tie}}^{{actual}} + \Delta P_{{dc}}$$

In the case of parallel interties, ΔP_tie_^Scheduled^ is described as

5 $$\Delta P_{{tie}} ^{{Scheduled}} = \sum\limits_{{i = 1}}^{3} {\sum\limits_{{j = 3}}^{4} {\alpha _{{ij}} \Delta P_{{Lj}} } } - \sum\limits_{{i = 4}}^{6} {\sum\limits_{{j = 1}}^{2} {\alpha _{{ij}} \Delta P_{{Lj}} } }$$

The error occurs due to scheduled tie line power as follows

6 $$\Delta P_{tie}^{Error} = \Delta P_{tie}^{Actual} - \Delta P_{tie}^{Scheduled}$$

Area control errors (ACEs) are outlined below for zones 1 and 2.

7 $$ACE_{1} = B_{1} \Delta F_{1} + \Delta P_{tie}^{Error}$$

8 $$ACE_{2} = B_{2} \Delta F_{2} + \alpha_{12} \Delta P_{tie}^{Error}$$

## The controller’s architecture

This section describes the various controllers like the combined FOPID + PFC, (1 + I^λ^D^µ^) + PFC, PII^ʎ^DD^µ^, PII^λ^D, PIDD, PID, and OARs. The Cuckoo search optimization algorithm (CSOA), SOA, genetic algorithm (GA) are used to find the optimize value of controller’s gain considering the objective function ISTSE. The control strategies are described as follows:

### The combined FOPID and polar fuzzy controller

#### Design of FOPID controller

Fractional calculus is used by FO controllers to solve differential equations. With this advancement, traditional calculus has become outdated. The Riemann–Liouville function represents the fractional difference-integral according to the usual definition.

9 $$aDt^{\alpha } f(t) = \frac{1}{\Gamma (n - \alpha )}\frac{{d^{n} }}{{dt^{n} }}\int\limits_{a}^{t} {(t - \tau )^{n - \alpha - 1} } f(\tau )d\tau$$

where n − 1 ≤ α < n, n is an integer, for fractional integral,

10 $$aDt^{ - \alpha } f(t) = \frac{1}{\Gamma (\alpha )}\int\limits_{a}^{t} {(t - \tau )^{\alpha - 1} } f(\tau )d\tau$$

Applying the Laplace transform,

11 $$L\{ aD_{t} ^{{ - \alpha }} f(t)\} = S^{\alpha } F(s) - sum_{{k = 0}}^{{n - 1}} S^{k} aDt^{{\alpha - k - 1}} f(t)|_{{t = 0}}$$

Using operators for FO increases the DOF and yields accurate solutions applied in various control-related applications according to numerous approximation methods that researchers are familiar with for fractional-order components. Oustaloup’s 5th-order approximation method is used in this paper. The same FO integral and derivative method is used throughout the optimization procedure. The approximated TF is provided by

12 $$O_{f} (s) = S^{\alpha } = K\;\Pi _{{ - N}}^{N} \frac{{s + \omega ^{\prime } k}}{{s + \omega k}}$$

The Oustaloup’s filter’s gains, zeros, and poles can be expressed as,

13 $$\omega k = \omega b\left( {\frac{\omega h}{{\omega b}}} \right)^{{\frac{{k + N + \frac{1}{2}(1 + \alpha )}}{2N + 1}}} ,\;\omega ^{\prime}k = \omega b\left( {\frac{\omega h}{{\omega b}}} \right)^{{\frac{{k + N + \frac{1}{2}(1 - \alpha )}}{2N + 1}}} ,\;K = \omega \;h^{\alpha }$$

Oustaloup’s filter order is 2N + 1 in Eq. (12). The 5th-order Oustaloup’s filter is employed in this research for FO operators^12^. This method creates a TF within the required frequency range [ωb, ωh] using a recursive distribution of N poles and N zeros. The simulation considers ωb = 0.01 rad/sec, ωh = 50 rad/sec, and N = 3. Here, "k" stands for the adjustable gain such that, at a frequency of 1 rad/s, the gain is 0 dB when k = 1 defines the frequencies of the zeros and poles and selects their number beforehand. The formula that describes the TF of a FOPID controller is as follows^22^:

14 $$u(s) = K_{p} + K_{i} \frac{1}{{s^{\lambda } }} + K_{d} s^{\mu }$$

K_p_, K_i_, and K_d_ are gains in this case. The parameters ʎ and µ are also employed for tuning.

#### Design of polar fuzzy controller (PFC)

**Fig. 5:**
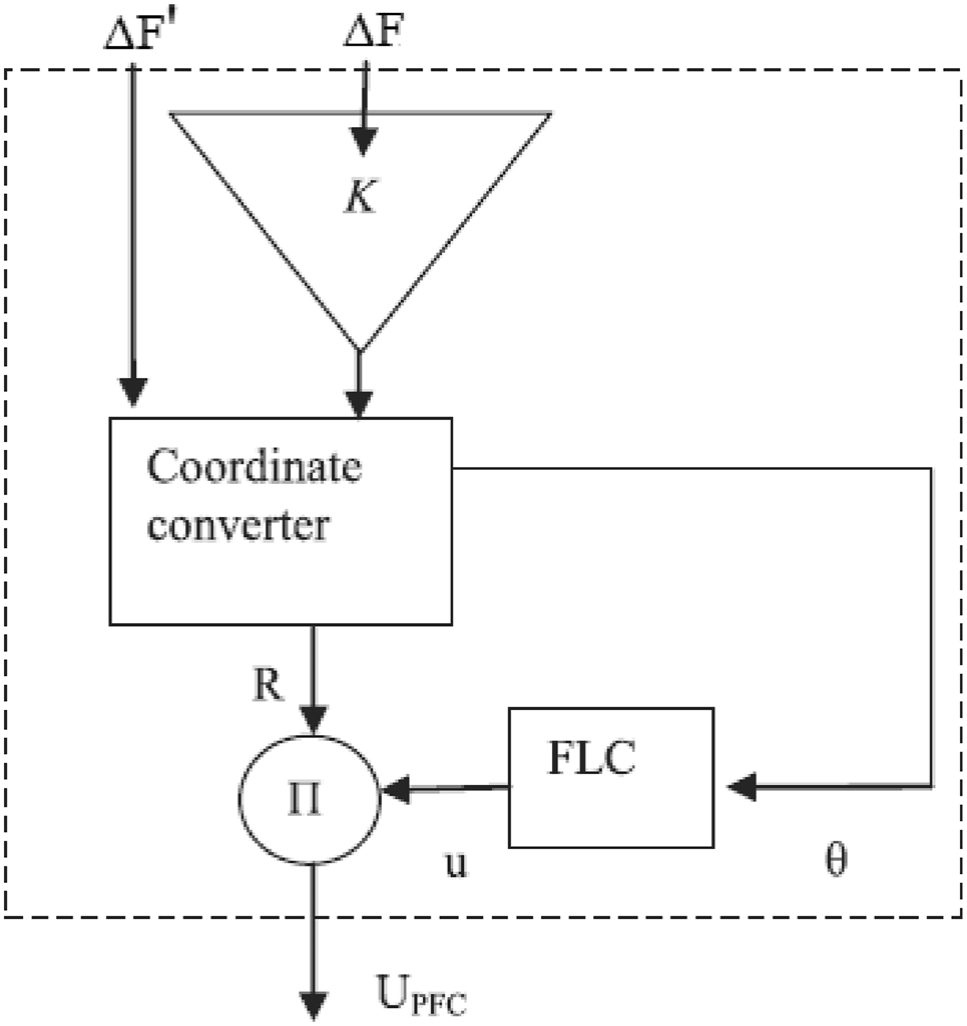
Structure of PFC.

**Fig. 6:**
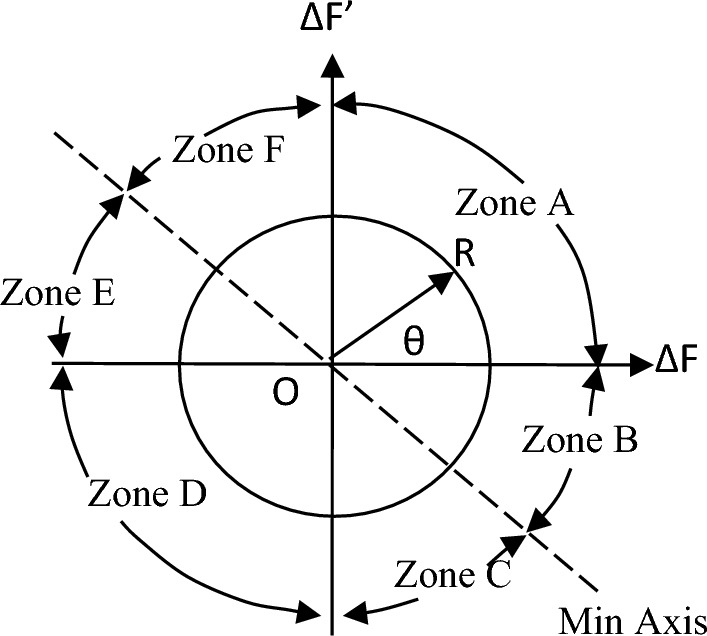
Plan for six zones.

**Fig. 7:**
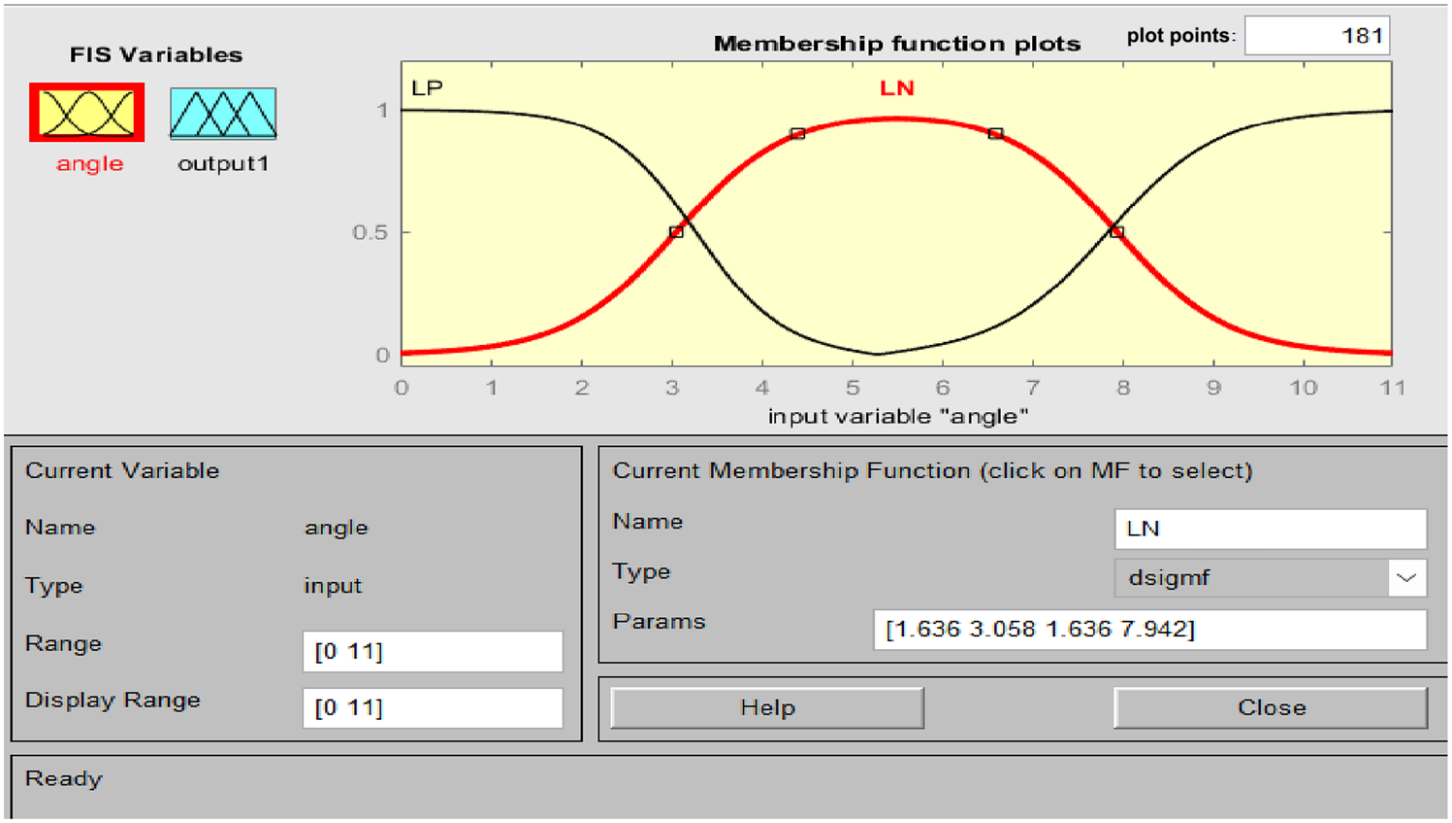
Fuzzy sets for input variable for PFC (dsigmf).

**Fig. 8:**
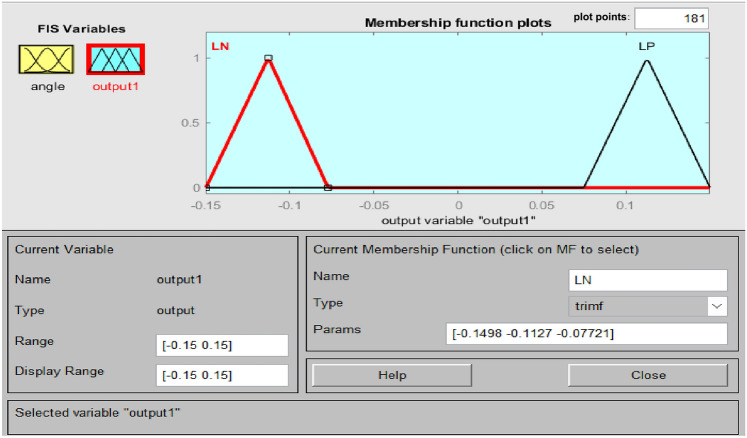
Fuzzy sets of output variable for PFC (trimf).

**Fig. 9:**
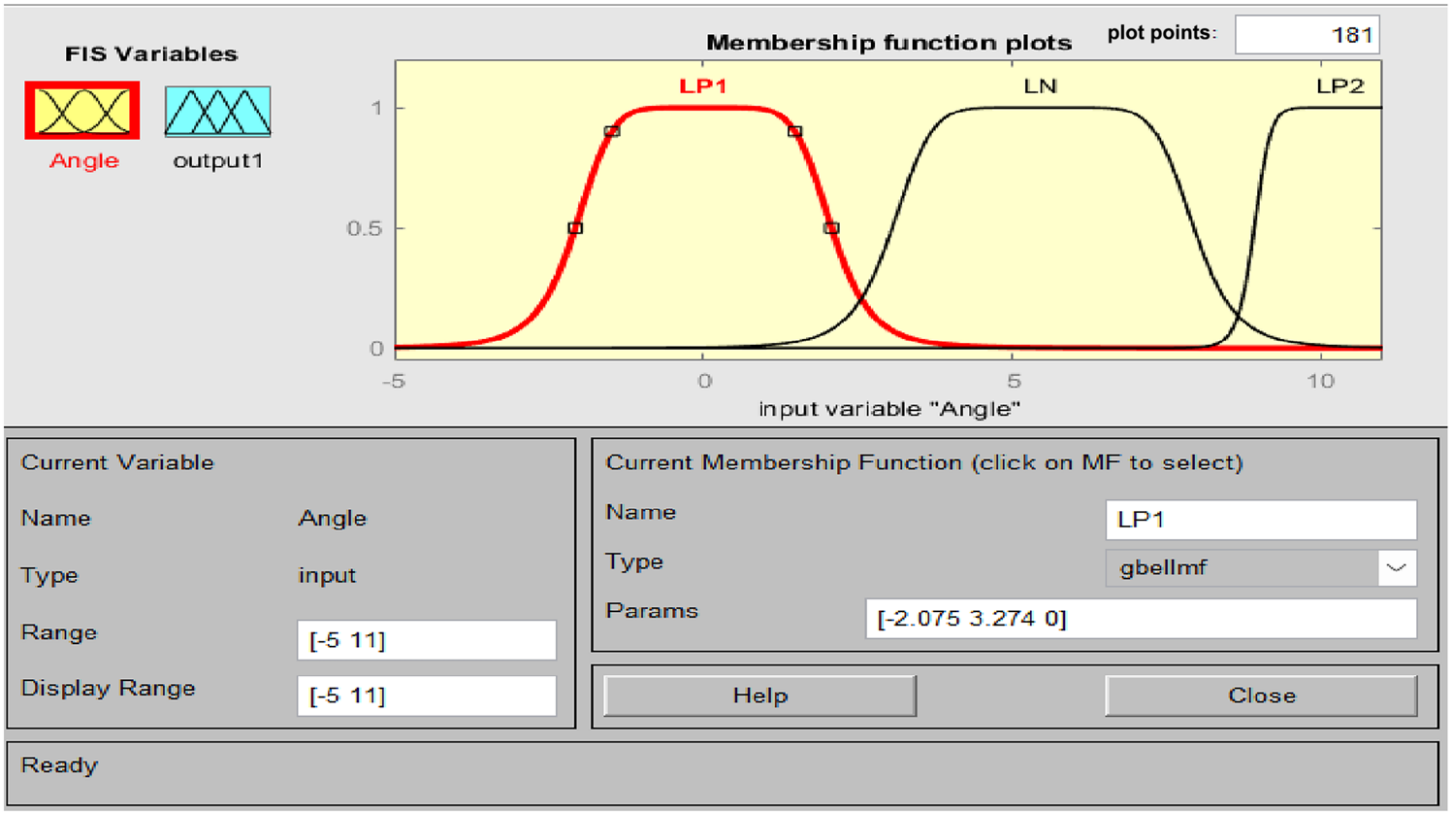
Fuzzy sets of input variable for PFC (gbellmf)^38^.

**Fig. 10:**
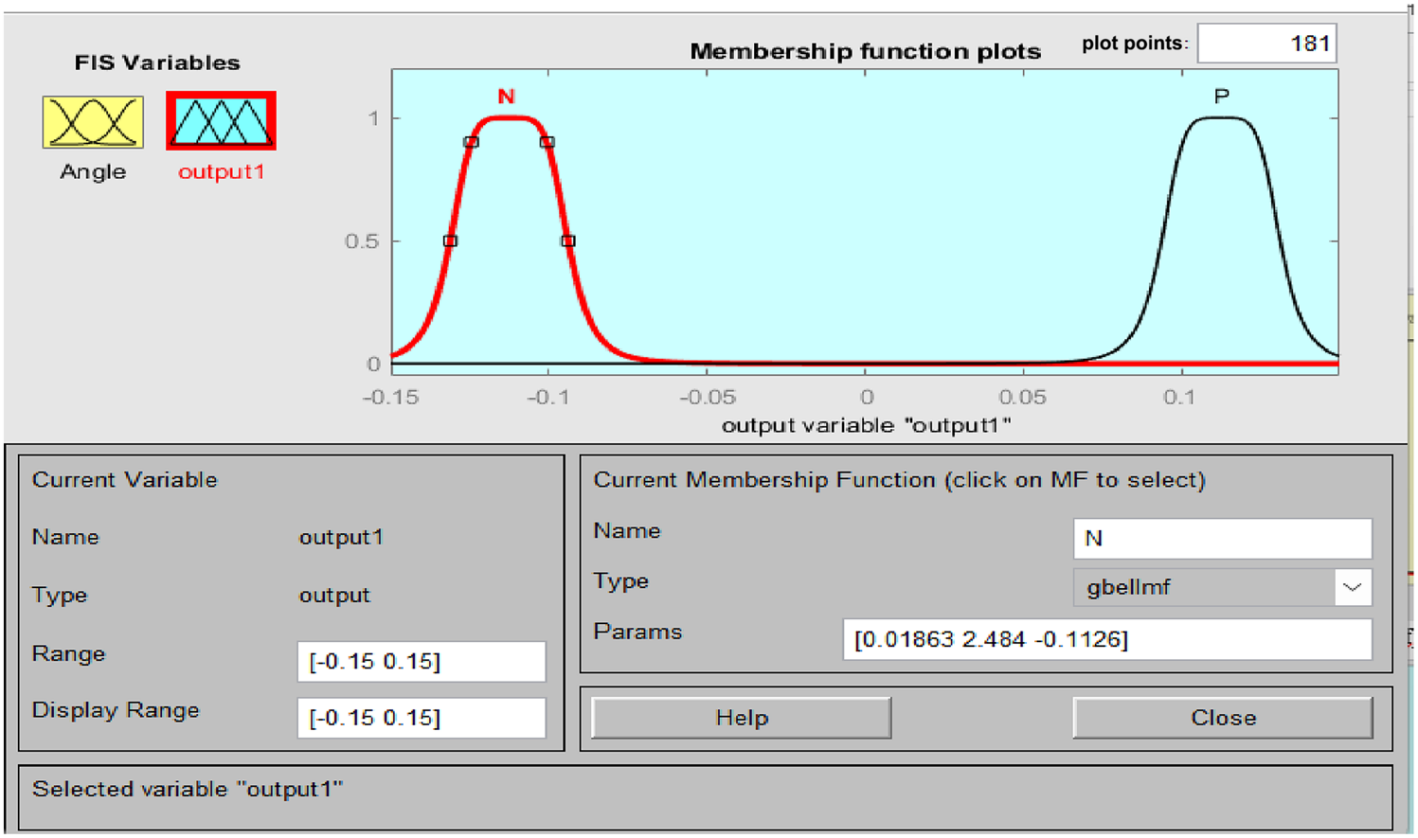
Fuzzy sets of output variable for PFC (gbellmf).

Accurate knowledge is required for the operation of an imprecise controller. Knowledge bases that contain more principles are more intricate, necessitating an increased amount of memory and processing power. Figure 5 illustrates the PFC structure. Using the variance in system frequency (∆F) and its integral (∆F’), the fuzzy controller derives the control action in polar coordinates for PFC. A fuzzy controller with polar quantities (PFC) has only two principles due to its simplicity. PFC can only utilize one input, angle, which is contingent upon the ratio of appropriately scaled inputs. Consequently, two input gains are not necessary for system frequency and integral adjustments. A single parameter is employed to calculate the ∆F or ∆F’. Figure 6's origin “0” represents the optimal equilibrium position. Consequently, vector R should be advanced in that direction by all control attempts. The output angle of the PFC is instantaneously determined by the imprecise input. FLC should be significantly positive for zone A (0°–90°) due to the fact that both ∆F and ∆F’ are positive. A low-positive FLC signal is necessary in Zone B (315°–360°). A low negative control signal from FLC is necessary for Zone C (270°–315°), and so on. Figures 7, 8 illustrate the fuzzy sets for the input and output variables of the PFC, respectively. The input and output membership functions are the dsigmoidal, and triangular. Rule 1 specifies that the output is P if the angle is LP, and Rule 2 declares that the output is N if the angle is LN. There are two straightforward principles that are taken into account when triangular is regarded as an output membership function^17^. A symmetrical shape that is comparable to a bell is known as the generalized bell-shaped membership function (gbellmf). Figures 9, 10 illustrate the fuzzy sets for the input and output variables of the PFC, respectively. The input and output membership functions are gbellmf. When generalized bell-shaped membership function (gbellmf) is considered as input as well output membership function. Most of the time, the range of variation of θ lies in first quadrant. So PFC operates in first quadrant. The first rule states that if the angle is LP1, the output is P. Rule 2: when (angle) equals LP2, (output) equals P; Rule 3 states that if the angle is LN, the output will be N.

**Fig. 11:**
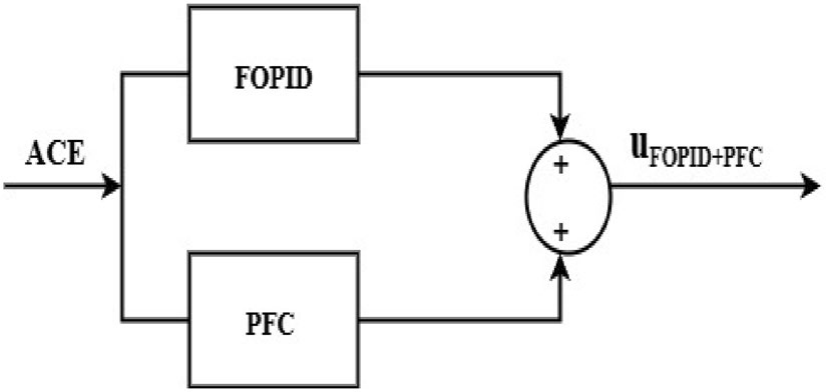
Block diagram of combined FOPID and PFC.

The design of the combined FOPID and PFC aims to advocate for a combination of the FOPID controller and PFC to enhance the dynamic responses of the proposed reformed T-G-S system. This combined controller provides superior capability in terms of flexibility, efficiency, and control, especially in systems that use various power sources. Figure 11 depicts the block diagram of the combined FOPID and polar fuzzy controller (FOPID + PFC). The primary objective of the LFC that has been developed is to minimize the frequency variations (ΔF_1_, ΔF_2_) and to minimize the tie-line power deviation (ΔP_tie_) that occurs as a result of system uncertainties. The combined FOPID and PFC settings are optimized by using an objective function, namely ISTSE, to accomplish the goal because of usage of FO operators boosts the DOF and provides precise solutions that have been used in multiple applications in the domain of control besides this a PFC quickly and with the most negligible computation returns the frequency and tie-line power to their nominal values.

### The combined (1 + FOID) and PFC (gbellmf) controller

To further improve the suggested reformed T-H-G system’s dynamic responses, this study proposes combining the (1 + FOID) controller with the PFC (gbellmf). Particularly in systems that use multiple power sources, a hybrid controller offers greater flexibility, efficiency, and control than a standard controller. The TF of a (1 + FOID) controller is given below:

15 $$u(s) = 1 + Ki\frac{1}{{S^{\lambda } }} + K_{d}S^{\mu }$$

**Fig. 12:**
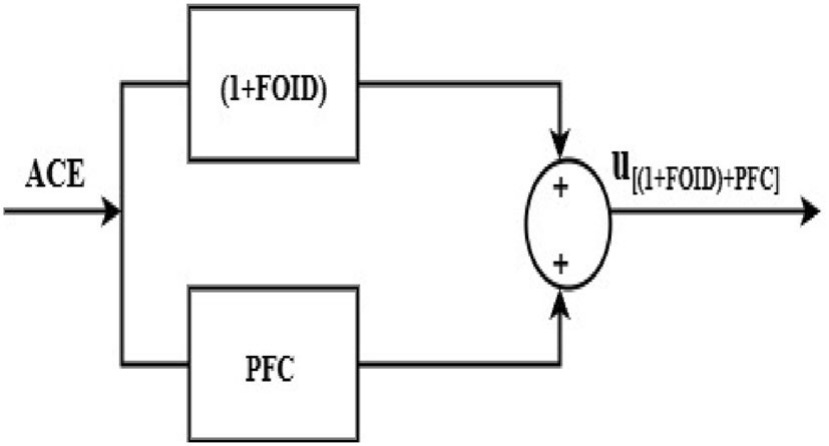
Block representation of (1 + FOID) + PFC.

The block diagram of a combined (1 + FOID) and PFC (gbellmf) controller is shown in Fig. 12. To reduce the ΔF_1_, ΔF_2_, and ΔP_tie_ caused by system uncertainties is the primary goal of the load frequency controller that has been created. Because the use of FO operators increases the DOF and provides precise solutions that have been used in multiple applications in the control domain, to do this, the objective function, ISTSE, is implemented to optimise the parameters of the combined (1 + FOID) and PFC (gbellmf) controller. In addition, a PFC quickly and with the least amount of computation returns to nominal values of the system.

### The ${\text{PII}}^{\lambda }$ DD^µ^ (PIILDDM) and ${\text{PII}}^{\lambda }$D (PIILD) controllers

**Fig. 13:**
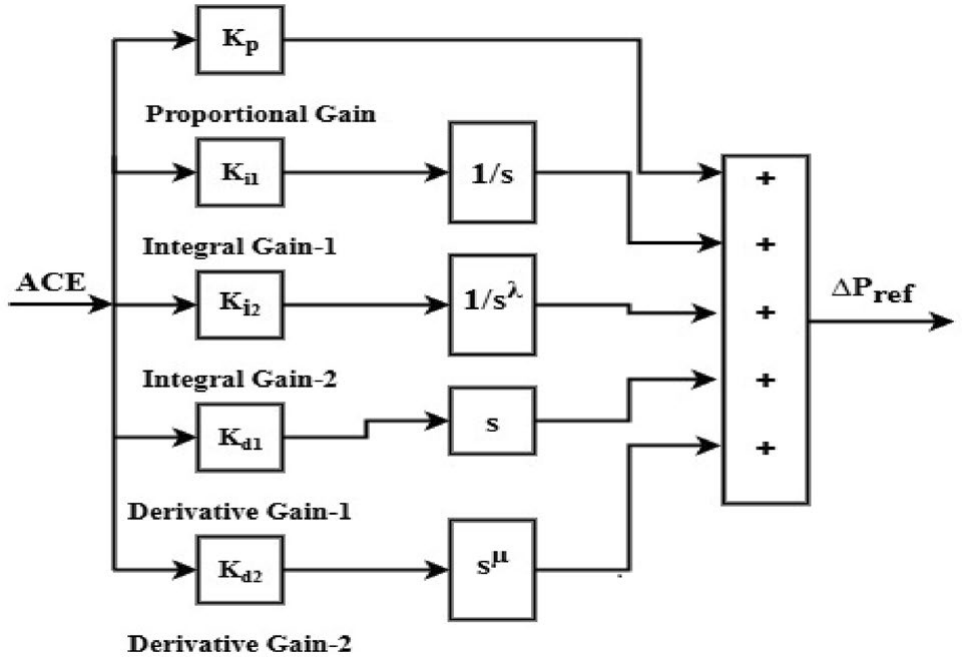
Block diagram of PII^ʎ^ DD^µ^ (PIILDDM) Controller.

Integer order PID and FOPID are combined to create the PII^ʎ^DD^µ^ controller. Using FO operators increases the DOF and yields accurate control domain solutions. Figure 13 displays the PIILDDM controller’s block diagram representation. The following is a mathematical description of it in the s-domain.

16 $$u_{{PIILDDM(S)}} = \left[ {K_{p} + K_{{i1*}} \frac{1}{S} + K_{{i2*}} \frac{1}{{S^{\lambda } }} + K_{{d1*S}} + K_{{d2*S^{\mu } }} } \right]$$

**Fig. 14:**
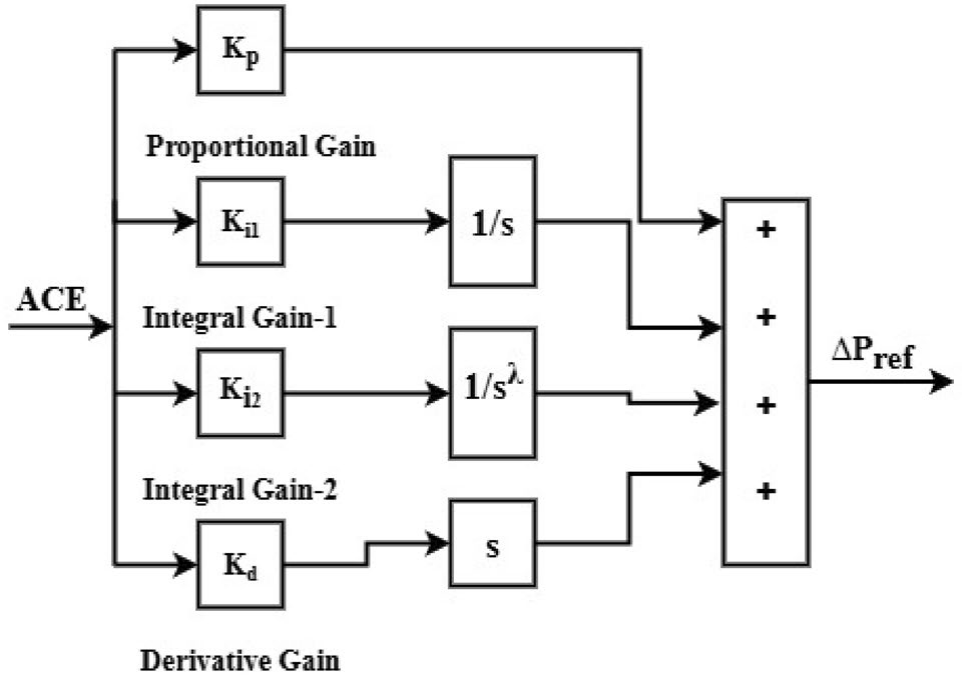
Block diagram of PII^ʎ^D (PIILD) controller.

In the PII^ʎ^D controller, the combination of integer order and FO of the integral controller boosts performance compared to the PID controller. Figure 14 shows a block diagram representation of the PIILD controller. It can be mathematically described in the s-domain as follows.

17 $$u_{{PIILD(S)}} = \left[ {K_{p} + K_{{i1*}} \frac{1}{S} + K_{{i2*}} \frac{1}{{S^{\lambda } }} + K_{{d*S}} } \right]$$

### The PID and PIDD Controllers

In process industries, PID feedback controllers are widely employed. Proportional controllers decrease the time it takes for a system to reach its desired state but do not eliminate any error that remains once the system has stabilized. Integral controls eradicate steady-state error but impair transient response. Derivative controls enhance the speed at which a system responds to changes, increase the reliability of the system and reduce overshooting. The process of designing a PID controller involves three gains namely proportional gains, integral gains, and derivative gains. The equation explains a primary PID controller in the time domain.

18 $$u_{{PID(t)}} = \left[ {K_{p} e(t) + Ki\int {e(t)dt} + K_{d} \frac{{de(t)}}{{dt}}} \right]$$

**Fig. 15:**
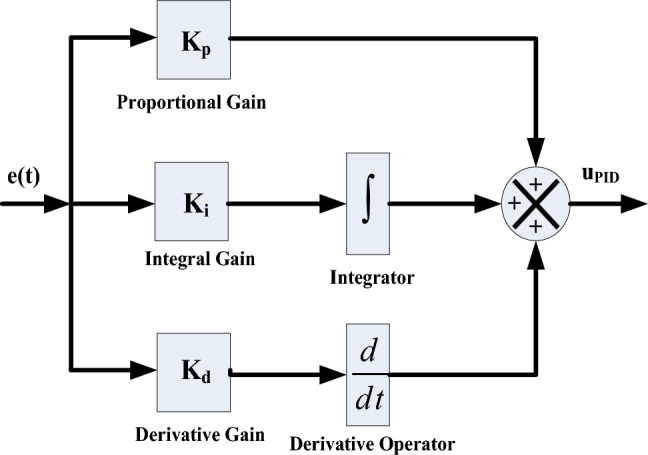
Block diagram of PID.

It can be mathematically described in the s-domain as follows. Figure 15 illustrates its block diagram representation

19 $$u_{{PID(S)}} = \left[ {K_{p} + K_{{i*}} \frac{1}{S} + K_{{d*S}} } \right]$$

The equation explains a PIDD controller in the time domain

20 $$u_{{PIDD(t)}} = \left[ {K_{p} e(t) + K_{i} \int {e(t)dt} + K_{{d1}} \frac{{de(t)}}{{dt}} + K_{{d2}} \frac{{de(t)}}{{dt}}} \right]$$

**Fig. 16:**
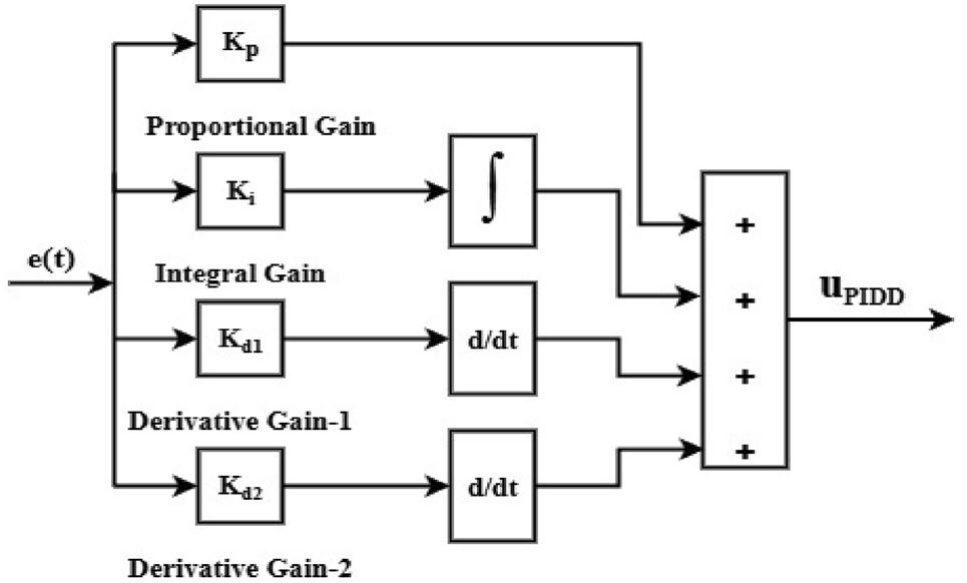
Block diagram of PIDD.

It can be mathematically described in the s-domain as follows. Figure 16 illustrates its block diagram representation

21 $$u_{{PIDD(S)}} = \left[ {K_{p} + K_{{i*}} \frac{1}{S} + K_{{d1*S}} + K_{{d2*S}} } \right]$$

In this article, the ISTSE is utilised as an objective function, which is described below:

22 $$J = \int\limits_{0}^{{t_{{sim}} }} {t^{2} } .\left\{ {\left( {\Delta F{}_{1}} \right)^{2} + \left( {\Delta F_{2} } \right)^{2} + \left( {\Delta P_{{tie}} } \right)^{2} } \right\}dt$$

In this context, t_sim_ stands for the simulation time. Therefore, the formulation of the issue may be defined as follows: minimizing J while adhering to the specific limitations listed below.

$$\begin{gathered} K_{{1,\min }} \le K_{1} \le K_{{1,\max ;}} K_{{2,\min }} \le K_{2} \le K_{{2,\max ;}} K_{{3,\min }} \le K_{3} \le K_{{3,\max ;}} K_{{4,\min }} \le K_{4} \le K_{{4,\max }} \hfill \\ K_{{p,\min }} \le K_{p} \le K_{{p,\max ;}} K_{{i,\min }} \le K_{i} \le K_{{i,\max ;}} K_{{d,\min }} \le K_{d} \le K_{{d,\max ;}} \lambda _{{\min }} \le \lambda \le \lambda _{{\max ;}} \mu _{{\min }} \le \mu \le \mu _{{\max }} \hfill \\ \end{gathered}$$

A situation in which the limits of K_1_, K_2_, K_3_, and K_4_ are set within the range [0, 50]. Additionally, the selection of the bounds of K_p_, K_i_, and K_d_ is done within the range of [0, 30]. Furthermore, the lower and upper limits of ʎ and µ are given within the limits of the range [0, 1]. It is evident in the table that the values that were given for the controller parameters range.

### The OAR for restructured T-H-G system

The system state space structure is stated using the equations shown below.

23 $$\dot{\underline {X} }{\text{ = A}}\,{\text{X}} + {\text{B}}\,{\text{U}} + {\text{F}}_{{\text{d}}} {\text{P}}_{{\text{d}}}$$

24 $${\text{Y}} = {\text{C}}\,{\text{X}}$$

where, $\dot{\underline {X} } =$ derivative of the state vector X is determined by the system matrix A, input distribution matrix B, output distribution matrix C, and disturbance distribution matrix Fd. These matrices are presented in Appendix A and have compatible dimensions. The state, control, load disturbance, and output vectors are denoted by X, U, P_d_, and Y, respectively. Equation (25) in the optimum control theory will be amended by removing the component F_d_ P_d_.

25 $$\dot{\underline {X} }{\text{ = A}}\,{\text{X}} + {\text{B}}\,{\text{U}};{\text{X}}\,\left( 0 \right) = {\text{X}}_{{\text{o}}}$$

Suppose the starting condition is X (0) = Xo. The vectors for X, U, and Pd are given as follows:

#### The EHVAC intertie

State vector:

$$\begin{aligned} \underset{\raise0.3em\hbox{$\smash{\scriptscriptstyle-}$}}{X} \left[ {{\text{25x1}}} \right] = & \left[ {\Delta {\text{F}}_{1} ,{\kern 1pt} \Delta {\text{F}}_{2} ,{\kern 1pt} \Delta {\text{P}}_{{{\text{G1}}}} ,{\kern 1pt} \Delta {\text{P}}_{{{\text{R1}}}} ,{\kern 1pt} \Delta {\text{X}}_{{{\text{E1}}}} ,{\kern 1pt} \Delta {\text{P}}_{{{\text{G2}}}} ,{\kern 1pt} \Delta {\text{X}}_{{{\text{h1}}}} ,{\kern 1pt} \Delta {\text{P}}_{{{\text{RH1}}}} ,} \right. \\ & \Delta {\text{P}}_{{{\text{G3}}}} ,{\kern 1pt} \Delta {\text{P}}_{{{\text{FC1}}}} ,{\kern 1pt} \Delta {\text{X}}_{{{\text{g1}}}} ,{\kern 1pt} \Delta {\text{P}}_{{{\text{VP1}}}} ,{\kern 1pt} \Delta {\text{P}}_{{{\text{G4}}}} ,{\kern 1pt} \Delta {\text{P}}_{{{\text{R2}}}} ,{\kern 1pt} \Delta {\text{X}}_{{{\text{E2}}}} ,{\kern 1pt} \Delta {\text{P}}_{{{\text{G5}}}} ,{\kern 1pt} \Delta {\text{X}}_{{{\text{h2}}}} , \\ & \left. {\Delta {\text{P}}_{{{\text{RH2}}}} ,{\kern 1pt} \Delta {\text{P}}_{{{\text{G6}}}} ,{\kern 1pt} \Delta {\text{P}}_{{{\text{FC2}}}} ,{\kern 1pt} \Delta {\text{X}}_{{{\text{g2}}}} ,{\kern 1pt} \Delta {\text{P}}_{{{\text{VP2}}}} ,{\kern 1pt} \Delta {\text{P}}_{{{\text{tie}}}} ,{\kern 1pt} \int {{\text{ACE}}_{1} {\text{dt}},} {\kern 1pt} \int {{\text{ACE}}_{2} } {\text{dt}}} \right]^{{\text{T}}} \\ \end{aligned}$$

Control vector:

$$\underline {U} \left[ {{\text{2x1}}} \right] = \left[ {\Delta {\text{P}}_{{{\text{c1}}}} ,\;\Delta {\text{P}}_{{{\text{c2}}}} } \right]^{{\text{T}}}$$

Disturbance vector:

$$\underline {P}_{{\underline {d} }} \left[ {{\text{6x1}}} \right] = \left[ {\Delta {\text{P}}_{{{\text{L1}}}} ,\,\Delta {\text{P}}_{{{\text{L2}}}} ,\,\Delta {\text{P}}_{{{\text{L3}}}} ,\,\Delta {\text{P}}_{{{\text{L4}}}} ,\,\Delta {\text{P}}_{{{\text{UC1}}}} ,\,\Delta {\text{P}}_{{{\text{UC2}}}} } \right]^{{\text{T}}}$$

#### The HVDC intertie

State vector:

$$\begin{aligned} \underset{\raise0.3em\hbox{$\smash{\scriptscriptstyle-}$}}{X} \left[ {{\text{25x1}}} \right] = & \left[ {\Delta {\text{F}}_{1} ,{\kern 1pt} \Delta {\text{F}}_{2} ,{\kern 1pt} \Delta {\text{P}}_{{{\text{G1}}}} ,{\kern 1pt} \Delta {\text{P}}_{{{\text{R1}}}} ,{\kern 1pt} \Delta {\text{X}}_{{{\text{E1}}}} ,{\kern 1pt} \Delta {\text{P}}_{{{\text{G2}}}} ,{\kern 1pt} \Delta {\text{X}}_{{{\text{h1}}}} ,{\kern 1pt} \Delta {\text{P}}_{{{\text{RH1}}}} ,} \right. \\ & \Delta {\text{P}}_{{{\text{G3}}}} ,{\kern 1pt} \Delta {\text{P}}_{{{\text{FC1}}}} ,{\kern 1pt} \Delta {\text{X}}_{{{\text{g1}}}} ,{\kern 1pt} \Delta {\text{P}}_{{{\text{VP1}}}} ,{\kern 1pt} \Delta {\text{P}}_{{{\text{G4}}}} ,{\kern 1pt} \Delta {\text{P}}_{{{\text{R2}}}} ,{\kern 1pt} \Delta {\text{X}}_{{{\text{E2}}}} ,{\kern 1pt} \Delta {\text{P}}_{{{\text{G5}}}} ,{\kern 1pt} \Delta {\text{X}}_{{{\text{h2}}}} , \\ & \left. {\Delta {\text{P}}_{{{\text{RH2}}}} ,{\kern 1pt} \Delta {\text{P}}_{{{\text{G6}}}} ,{\kern 1pt} \Delta {\text{P}}_{{{\text{FC2}}}} ,{\kern 1pt} \Delta {\text{X}}_{{{\text{g2}}}} ,{\kern 1pt} \Delta {\text{P}}_{{{\text{VP2}}}} ,{\kern 1pt} \Delta {\text{P}}_{{dc}} ,{\kern 1pt} \int {{\text{ACE}}_{1} {\text{dt}},} {\kern 1pt} \int {{\text{ACE}}_{2} } {\text{dt}}} \right]^{{\text{T}}} \\ \end{aligned}$$

Control vector:

$$\underline {U} \left[ {{\text{2x1}}} \right] = \left[ {\Delta {\text{P}}_{{{\text{c1}}}} ,\;\Delta {\text{P}}_{{{\text{c2}}}} } \right]^{{\text{T}}}$$

Disturbance Vector:

$$\underline {P}_{{\underline {d} }} \left[ {{\text{6x1}}} \right] = \left[ {\Delta {\text{P}}_{{{\text{L1}}}} ,\,\Delta {\text{P}}_{{{\text{L2}}}} ,\,\Delta {\text{P}}_{{{\text{L3}}}} ,\,\Delta {\text{P}}_{{{\text{L4}}}} ,\,\Delta {\text{P}}_{{{\text{UC1}}}} ,\,\Delta {\text{P}}_{{{\text{UC2}}}} } \right]^{{\text{T}}}$$

#### Taking into account ∆P_dc_ as another control variable in parallel interties

State vector:

$$\begin{aligned} \underset{\raise0.3em\hbox{$\smash{\scriptscriptstyle-}$}}{X} \left[ {{\text{26x1}}} \right] = & \left[ {\Delta {\text{F}}_{1} ,{\kern 1pt} \Delta {\text{F}}_{2} ,{\kern 1pt} \Delta {\text{P}}_{{{\text{G1}}}} ,{\kern 1pt} \Delta {\text{P}}_{{{\text{R1}}}} ,{\kern 1pt} \Delta {\text{X}}_{{{\text{E1}}}} ,{\kern 1pt} \Delta {\text{P}}_{{{\text{G2}}}} ,{\kern 1pt} \Delta {\text{X}}_{{{\text{h1}}}} ,{\kern 1pt} \Delta {\text{P}}_{{{\text{RH1}}}} ,} \right. \\ & \Delta {\text{P}}_{{{\text{G3}}}} ,{\kern 1pt} \Delta {\text{P}}_{{{\text{FC1}}}} ,{\kern 1pt} \Delta {\text{X}}_{{{\text{g1}}}} ,{\kern 1pt} \Delta {\text{P}}_{{{\text{VP1}}}} ,{\kern 1pt} \Delta {\text{P}}_{{{\text{G4}}}} ,{\kern 1pt} \Delta {\text{P}}_{{{\text{R2}}}} ,{\kern 1pt} \Delta {\text{X}}_{{{\text{E2}}}} ,{\kern 1pt} \Delta {\text{P}}_{{{\text{G5}}}} ,{\kern 1pt} \Delta {\text{X}}_{{{\text{h2}}}} , \\ & \left. {\Delta {\text{P}}_{{{\text{RH2}}}} ,{\kern 1pt} \Delta {\text{P}}_{{{\text{G6}}}} ,{\kern 1pt} \Delta {\text{P}}_{{{\text{FC2}}}} ,{\kern 1pt} \Delta {\text{X}}_{{{\text{g2}}}} ,{\kern 1pt} \Delta {\text{P}}_{{{\text{VP2}}}} ,{\kern 1pt} \Delta {\text{P}}_{{tie}} ,{\kern 1pt} \Delta {\text{P}}_{{dc}} ,{\kern 1pt} \int {{\text{ACE}}_{1} {\text{dt}},} {\kern 1pt} \int {{\text{ACE}}_{2} } {\text{dt}}} \right]^{{\text{T}}} \\ \end{aligned}$$

 Control vector :

$$\underline {U} \left[ {{\text{3x1}}} \right] = \left[ {\Delta {\text{P}}_{{{\text{c1}}}} ,\;\Delta {\text{P}}_{{{\text{c2}}}} ,\;\Delta {\text{P}}_{{{\text{dc}}}} } \right]^{{\text{T}}}$$

Disturbance vector:

$$\underline {P}_{{\underline {d} }} \left[ {{\text{6x1}}} \right] = \left[ {\Delta {\text{P}}_{{{\text{L1}}}} ,\,\Delta {\text{P}}_{{{\text{L2}}}} ,\,\Delta {\text{P}}_{{{\text{L3}}}} ,\,\Delta {\text{P}}_{{{\text{L4}}}} ,\,\Delta {\text{P}}_{{{\text{UC1}}}} ,\,\Delta {\text{P}}_{{{\text{UC2}}}} } \right]^{{\text{T}}}$$

The design methodology of the OAR is described in^31^.

### The Cuckoo Search optimization algorithm (CSOA)

The gains must be fine-tuned in order to achieve the system’s desired efficiency. There is no well-defined approach in the literature for determining the appropriate gains of new controllers like FOPID + PFC and PIILDDM. This approach is utilized to discover the tuned gains of all the controllers proposed in this study. Yang and Deb suggested using CSOA, a biologically based meta-heuristic optimization algorithm^32^. This algorithm has broad capabilities to efficiently solve multidimensional problems and a robust, searchable capability, like other traditional bio-inspired algorithms such as GA, DE, and PSO algorithms. Diversification and intensification dominate cuckoo research optimization algorithms over different well-known algorithms such as GA and PSO.

**Fig. 17:**
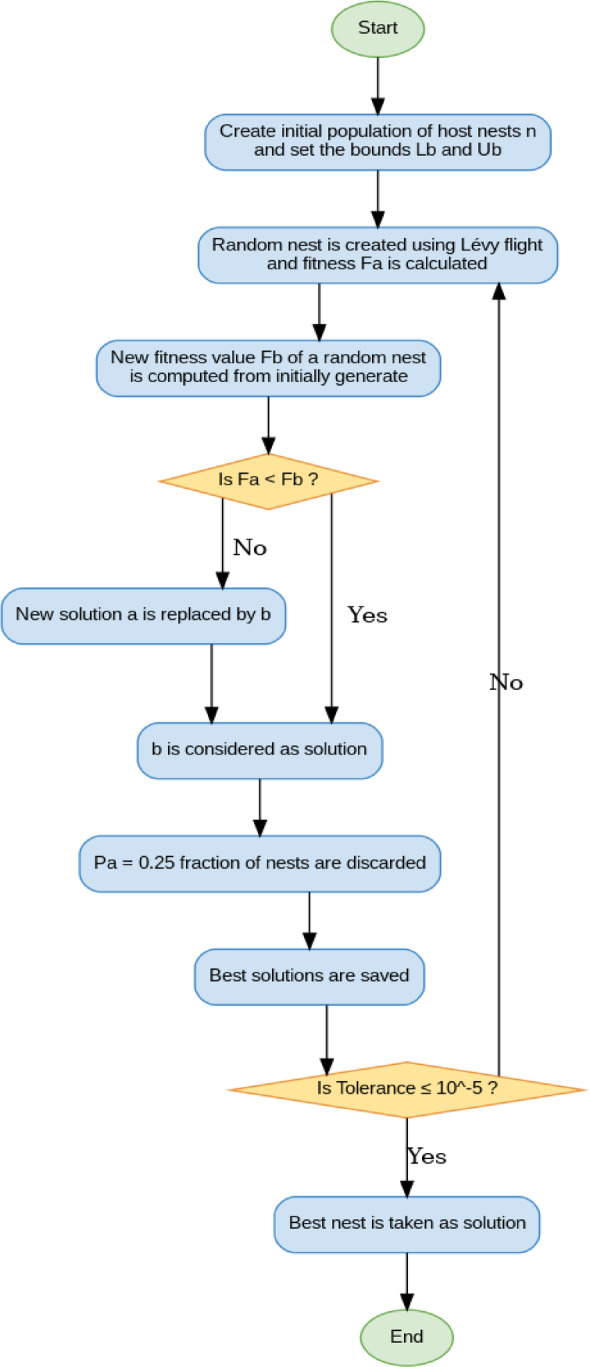
Flow chart of CSOA.

The Cuckoo bird is famous for its pleasant tone and wonderful reproductive strategies. Cuckoo birds lay their eggs in shared nests in certain habitats. They sometimes eliminate other birds’ eggs from shared nests to increase their own eggs’ hatchability. If a host bird discovers that the eggs are not its own, it can either remove the foreign eggs from the nest or abandon the nest and start a fresh one elsewhere. Female cuckoos have certain unique features similar to the color and design of the eggs laid by a few carefully selected host birds. Their eggs are less likely to be abandoned while using this process, which increases efficiency. Female cuckoos often learn when other birds lay their eggs, and parasitic cuckoos sometimes discover a nest where the host bird has only laid eggs. Cuckoo eggs hatch faster than host eggs, and the cuckoo evicts the host eggs until the first cuckoo egg hatches. This behaviour raises the host bird’s food supply for the cuckoo’s chick. The Lévy flight principle is incorporated into the CSOA technique, which improves its searching capabilities^33^. It is a form of flight behaviour. Several animals abruptly change 90 degrees from their traditional flight paths, resulting in an erratic scale-free search pattern in the Lévy flight style. It has been shown that when such behaviour is applied to optimum search, the findings display promise. Figure 17 depicts the flow chart of the CSOA process. Table 1 lists the various CSOA parameters used to maximize the controller’s gains. The authors benchmarked CSOA against PSO, GWO, and HHO by utilizing the same assessment budgets, parameter tweaking methods, and seeds across the duration of distinct AGC scenarios. These scenarios included uncertainty and renewable variability. By reducing the effort required by the actuator, CSOA regularly achieved the lowest median target, indicating a 7–15% improvement. In addition to this, it converged to near-optimal solutions 30–40% faster, and it displayed superior resilience by successfully fulfilling constraints with excellent success rate.

**Table 1:** The parameters considered for tuning of controllers using CSOA.

Design parameter	Value
Number of Nests	25
Tolerance	1.0E-9
Step size	1.5
Probability of alien eggs	0.25
Maximum number of iterations	100

The weighted total of the IACCO and the ISTSE has been used as the objective function.

26 $$IACCO=\int \left|\left(u\left(t\right)-u\left(t-1\right)\right)\right|dt$$

27 $$\text{OBF}=({w}_{1}*ISTSE) + ({w}_{2} * IACCO)$$

The values for ${w}_{1}$ and ${w}_{2}$ are 0.999 and 0.001, respectively.

### The SOA

**Fig. 18:**
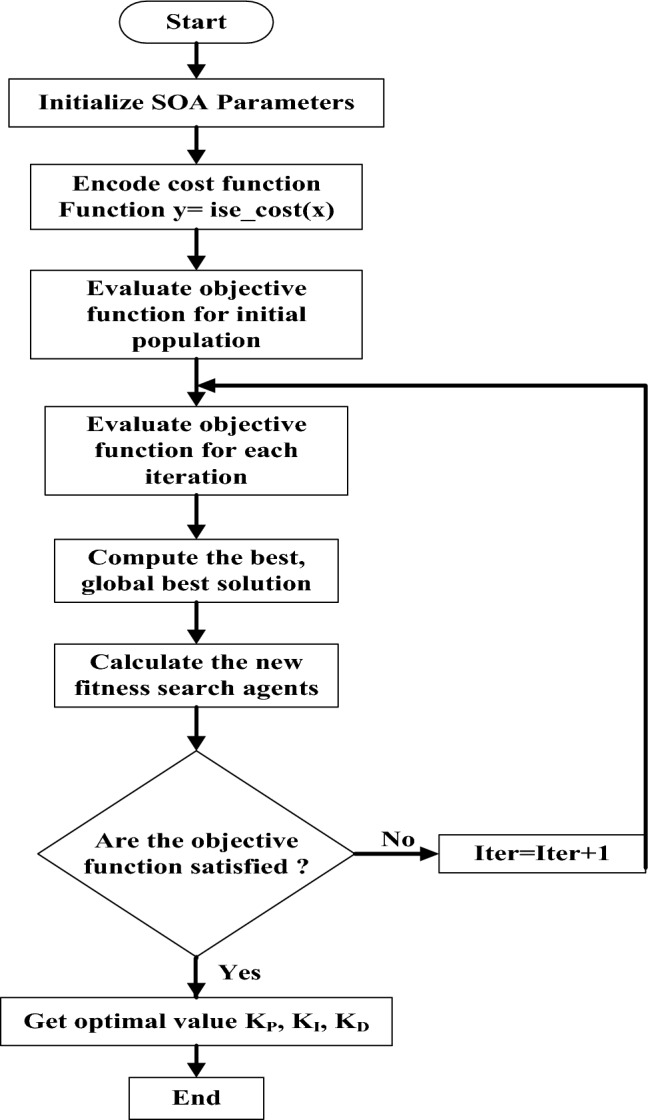
The flow chart of SOA.

The SOA is an innovative metaheuristic optimisation tool inspired by human skill growth and augmentation. Honest individuals who are constantly developing and learning participate in population-based SOA. Members of a SOA can solve an optimization problem. You may see the SOA flowchart in Fig. 18. These members’ search space placements determine the value of the problem decision variables. Assignments of SOA members are initially made at random. The population of SOAs may be represented by a matrix X (Eq. 28).

28 $$X = \left[ \begin{gathered} X_{1} \hfill \\ \; \vdots \hfill \\ X_{i} \hfill \\ \; \vdots \hfill \\ X_{N} \hfill \\ \end{gathered} \right] = \left[ \begin{gathered} x_{{1,1}} \cdots x_{{1,\,d}} \cdots x_{{1,\,m}} \hfill \\ \; \vdots \;\;\; \ddots \;\; \vdots \;\;\; \ddots \;\; \vdots \hfill \\ x_{{i,\,1}} \cdots x_{{i,\,d}} \cdots x_{{i,\,m}} \hfill \\ \; \vdots \;\;\; \ddots \;\; \vdots \;\;\; \ddots \;\; \vdots \hfill \\ x_{{N,\,1}} \cdots x_{{N,\,d}} \cdots x_{{N,\,m}} \hfill \\ \end{gathered} \right]$$

To seek out the desired function’s value, the issue variable is presented to each participant. Consequently, according to Eq. (29), a vector is a value derived from the ith candidate solution, and F is a type of vector that contains every value of the desired factor as follows:

29 $$F = \left[ \begin{gathered} F_{1} \hfill \\ \; \vdots \hfill \\ F_{i} \hfill \\ \; \vdots \hfill \\ F_{N} \hfill \\ \end{gathered} \right]_{{N \times 1}} = \left[ \begin{gathered} F(X_{1} ) \hfill \\ \;\;\;\; \vdots \hfill \\ F(X_{i} ) \hfill \\ \;\;\;\; \vdots \hfill \\ F(X_{N} ) \hfill \\ \end{gathered} \right]_{{N \times 1}}$$

Updates on SOA populations cover both exploration and exploitation. The exploring stage mimics picking up a talent from a professional. You’ll mimic the way users’ skills improve via exploitation. The update process in SOA design is divided into two stages: exploitation and exploration. Exploration looks for solutions on a global scale, whereas exploitation looks locally. During the inquiry phase, SOA members did not follow the best person’s instructions but followed each other’s. This improves the algorithm’s ability to explore and locate the ideal location by enabling it to search the search space more effectively. The algorithm converges on more plausible solutions during the exploitation phase with local searches close to each population member.

A community expert first teaches a skill to SOA participants. A person’s objective function is closely correlated with their contribution to the population. A member of the SOA is considered an expert. Higher objective function value members are part of the “team of experts” for an SOA participant. At random, one of these individuals will serve as the mentor for the individual. For this reason, the expert chosen to guide the SOA member might not be the best option. The best option is an expert set member who does not rotate and is applicable to every SOA member. The population learns the algorithm’s global search and exploration skills and is directed to different search spaces. Experts deal with this. It might be acceptable if the target function is increased by the new position projected for each population member.

Equations may define the update’s first phase by the notions presented.

30 $$X_{i} ^{{P1}} \vdots X_{{i,d}}^{{P1}} = x_{{i,d}} + r\,X\,(E_{{i,d}} - 1\;X\;x_{{i,d}} ),E_{i} = X$$

31 $$\begin{gathered} Where\;F_{k} \prec F_{i} \;\;and\;k\;is\;randomly\;selected\;from\;\left\{ {1,2,...N} \right\},k \ne i \hfill \\ Xi = \left\{ {\begin{array}{*{20}l} {X_{i}^{{p1}} } \hfill & {,\,F_{i}^{{p1}} \prec F_{i} } \hfill \\ {X_{i} } \hfill & {,\,else} \hfill \\ \end{array} } \right\} \hfill \\ \end{gathered}$$

The newly estimated status of the ith candidate solution is represented by X_i_^P1^ based on the first phase, and so forth. In the second stage, everyone develops their skills on their own to improve. In order to maximise the value of its goal function, every participant searches for better circumstances nearby, which makes SOA more exploitative. This demonstrates expertise and encourages exploitation.

In the next phase, everyone works independently to develop their skills. The newly estimated position is acceptable if the target function is raised, just like in the previous stage. SOA updating properties are mathematically described via equations.

32 $$X_{i}^{{p2}} \vdots X_{{i,d}}^{{p2}} \; = \left\{ {\begin{array}{*{20}l} {x_{{i,d}} \; + \frac{{1 - 2r}}{t}X\;x_{{i,d}} \;,} \hfill & {r \prec 0.5} \hfill \\ {x_{{i,d}} \; + \frac{{lb_{{i,d}} (ub_{j} - lb_{j} )}}{t}X\;x_{{i,d}} ,} \hfill & {else} \hfill \\ \end{array} } \right\}$$

33 $$X_{i} = \left\{ {\begin{array}{*{20}l} {X_{i}^{{p1}} ,} \hfill & {F_{i}^{{p1}} \prec Fi} \hfill \\ {X_{i} ,} \hfill & {else} \hfill \\ \end{array} } \right\}$$

The most current status computation of the ith candidate solution is reflected in X_i_^P2^. The initial evaluation of the objective function and the initialization of the population are equivalent to O (Nm), in which m is the total number of issue variables and N is the size of the overall population. The updated member’s location about the objective function is assessed at each iteration and phase of the two-stage SOA member update process. In the reconfiguration process, the computational challenge of SOA is O (2NmT). Thus, the sum of the SOA’s complexity is O (Nm (1 + 2 T))^34,35^.

### Genetic algorithm (GA)

**Fig. 19:**
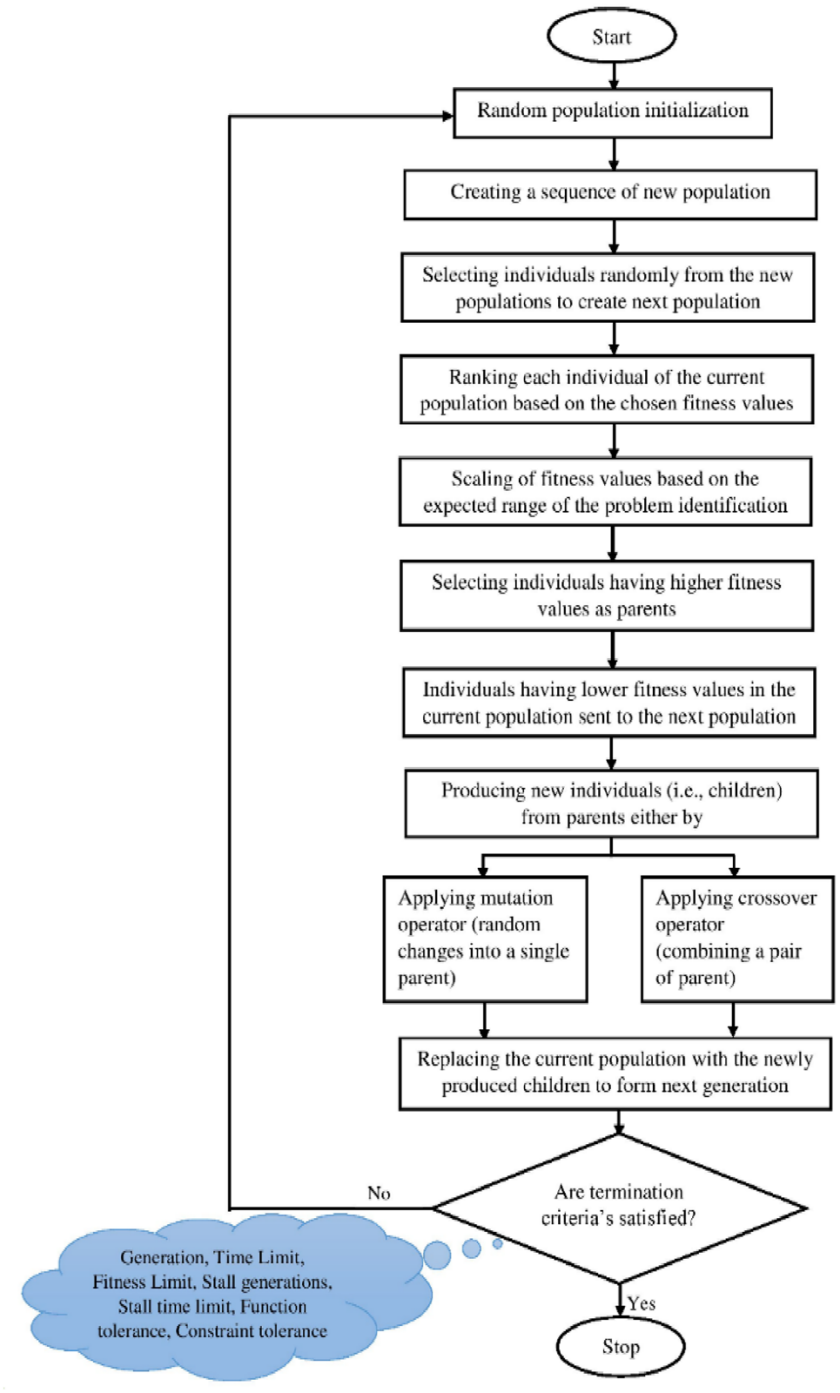
The framework of the GA flow chart.

This algorithm employs the Darwinian principle of evolution, which symbolises the motto “survival of the fittest.” The GA schematic is displayed in Fig. 19. In terms of random probability distributive optimization, it is a member of the evolutionary algorithm class. Bit strings that store GA solution variables are called chromosomes or solution vectors. These chromosomes replicate Natural evolution, created by crossing, reproduction, and mutation across generations. An objective function is necessary for optimization. GA markers of note include crossover percent and mutation fraction. The mutation rate in the population that is not part of the elite is determined by the use of mutation criteria. The quantity of non-elite participants who progressed through the crossover is indicated by the crossover guidelines. Select the conditions for crossover and mutation carefully^36^.

## The hybrid ESS

The energy storage systems (ESS) are frequently utilized in order to upgrade the realization of the system^15^. When generation surpasses demand, these act as backup devices, storing excess power and replenishing the grid with electricity when demand exceeds supply. This keeps the systems operating securely and balances the supply and demand sides. As a result, it ensures that changes in system frequency fall within reasonable limits^19^. Battery energy storage systems (BESS) are expensive. The EV’s battery can increase the system’s frequency stability to get around this problem. Researchers have used RFB in conjunction with SMES and UC in various settings. In^20^, the SMES + RFB, FES + RFB, and UC + RFB combinations were used. In this study, the EV and UC are coupled to create a hybrid ESS to increase the system’s frequency stability. Instead of small batteries, it is similar to large-scale energy storage systems. The EV and UC are discussed separately. The hybrid ESS is presented in Fig. 22.

### Electric vehicle (EV)

The aggregated Electric Vehicle (EV) fleet is represented by an equivalent BESS model. The state-of-charge (SoC) dynamics is expressed as:

34 $$\frac{{dSoC_{{EV}} (t)}}{{dt}} = - \frac{{\eta _{c} P_{{ch}} (t)}}{{E{}_{{nom}}}} + \frac{{P_{{dis}} (t)}}{{\eta _{d} E_{{nom}} }}$$

where P_ch_ and P_dis_ denote charging and discharging power, E_nom_ is the nominal energy capacity, and $\eta c$, $\eta d$ are the charge/discharge efficiencies. The SoC is limited to [SoC ^min^, SoC^max^] and charging and discharging power is constrained by [P^min^, P^max^].

EVs are connected probabilistically, modeled via an availability factor A (t) derived from stochastic plug-in/plug-out behavior. The effective power support is then

35 $$P_{{EV}} (t) = A(t).(P_{{ch}} (t) - P_{{dis}} (t))$$

**Fig. 20:**
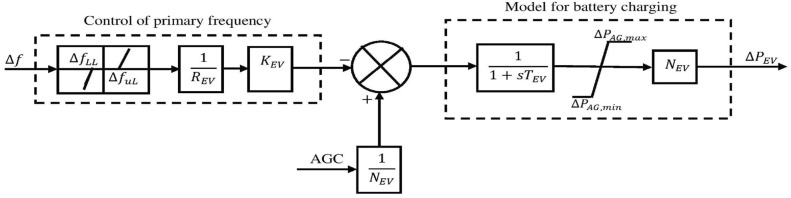
Structure of EV.

It contributes to AGC via a participation factor α_EV_, which dynamically adjusts based on SoC and fleet availability. The $\Delta P_{{EV}}$ denotes the incremental generation change of an EV^31^. The structure of the EV is illustrated in Fig. 20, which also demonstrates the maximum and lowest power outputs of EV fleets. It is computed as follows:

36 $$\Delta P_{{AG,\max }} = + \left( {\frac{1}{{N_{{EV}} }}} \right)\Delta P_{{EV}}$$

37 $$\Delta P_{{AG,\min }} = - \left( {\frac{1}{{N_{{EV}} }}} \right)\Delta P_{{EV}}$$

The EV’s State of Charge (SOC) can be used to calculate the K_EV_ value.

38 $$K_{{EV}} = K_{{\max }} \left\{ {1 - \left( {\frac{{SOC - SOC_{{low(high)}} }}{{SOC_{{\max (\min )}} - SOC_{{low(high)}} }}} \right)^{{^{n} }} } \right\}$$

The symbol N_EV_ indicates how many connected EVs there are. The TF of EV is described by the following equation:

39 $$G_{{EV}} = \left\{ {\frac{{K_{{EV}} }}{{1 + sT_{{EV}} }}} \right\}$$

The gain and time constant of EVs are indicated as the K_EV_ and T_EV_, respectively.

### Ultra capacitor (ESU)

**Fig. 21:**
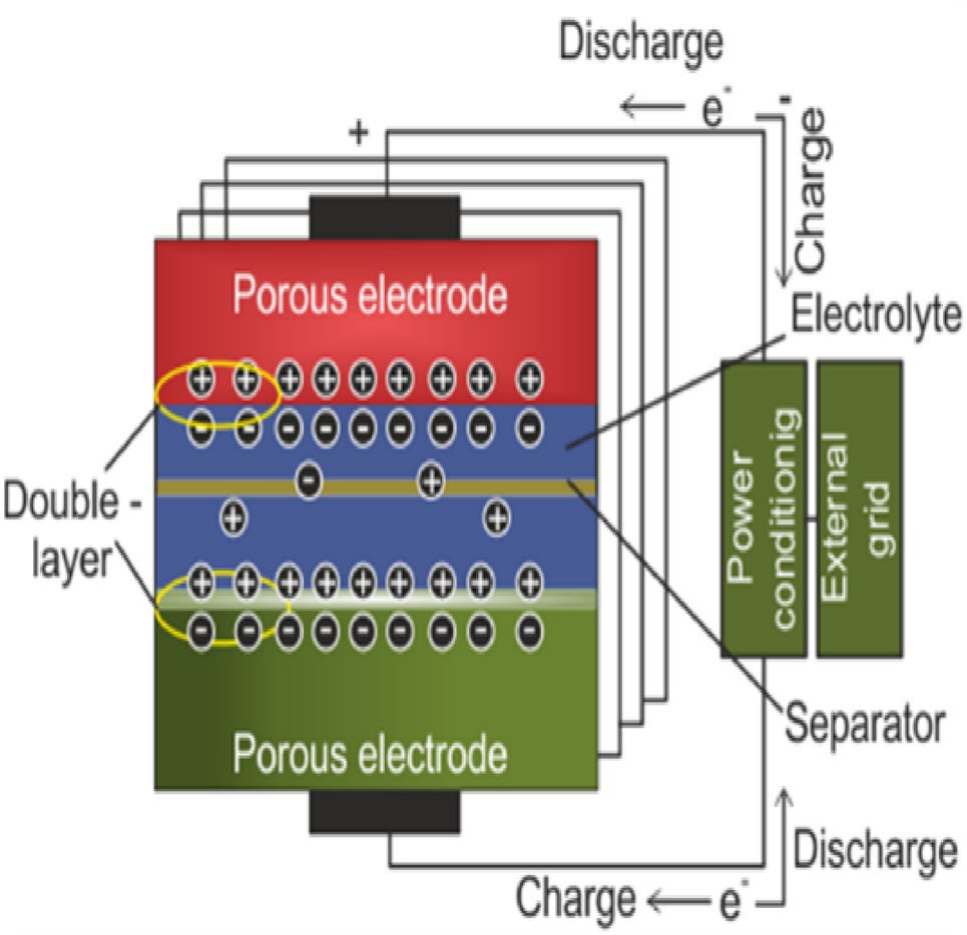
Ultra-capacitor (UC) in frequency management.

Ultra-capacitor (UC) is a super capacitor. Its surface area is more significant than that of capacitors. The UCs have 100–1000 times the capacitance of electrolytic capacitors. UC has a higher power density than batteries. Power varies from 1000 to 5000 W/kg, whereas specific energy is 1 to 10 Wh/kg. It charges and discharges quicker than batteries. Therefore, UC is dependable and AGC-friendly^31^. An ultra-capacitor (UC) serves the purpose of frequency control, as shown in Fig. 21. The frequency fluctuation of the control zone will serve as the input to the UC, and its transfer function is as follows:

40 $$\Delta P_{{UC}} (s) = \left\{ {\frac{{K_{{UC}} }}{{1 + sT_{{UC}} }}} \right\}\Delta F(s)$$

The hybrid ESS unit is modeled as an additional control input in the AGC loop. During disturbances, the AGC signal is decomposed into fast and slow components. Fast components are routed to the UC, while slow components are dispatched to the EV fleet. This decomposition improves overall frequency stability while minimizing wear on each storage device. The schematic diagram is as follows:

Show AGC → signal decomposition → UC control loop (fast) → EV control loop (slow) → hybrid power injection.

## Simulation outcomes and discussion

### The performance evaluation of combined FOPID + PFC for T-G-S system

**Fig. 22:**
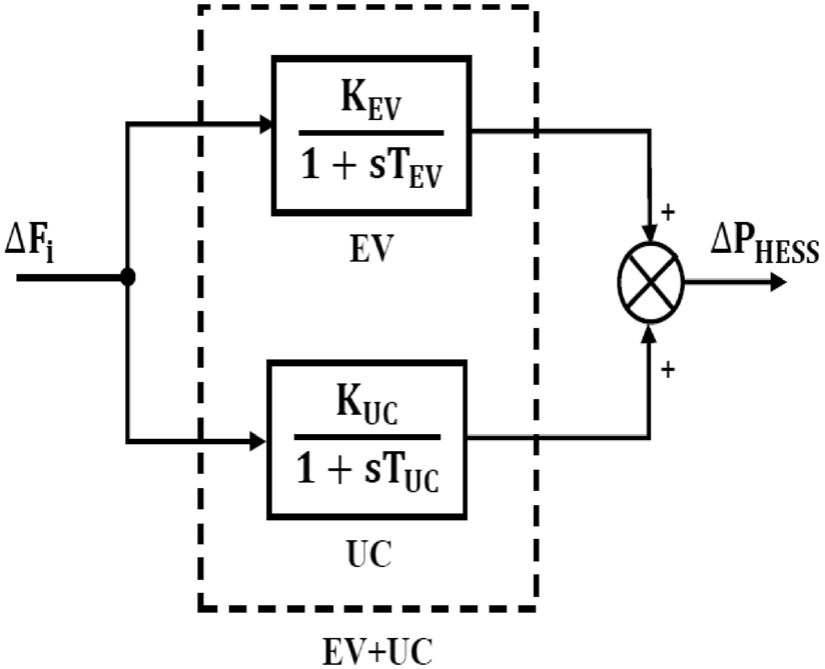
TF of hybrid ESS (EV + UC).

**Fig. 23:**
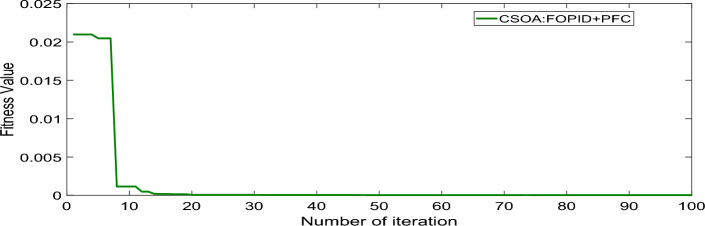
Convergence characteristics with CSOA: FOPID + PFC.

The system was simulated with the help of MATLAB/SIMULINK. The GA, and CSOA are written in m files. The parameters considered for tuning of controllers using CSOA are shown in Table 1. The factors that influence area involvement are as follows: apf_1_ = 0.6; apf_2_ = apf_3_ = 0.2. This section examines how the restructured T-G-S system performs in respect to certain uncertainties (Fig. 22). When compared to each controller, the hybrid FOPID + PFC controller that has been developed obtains a lower fitness value and a better dynamic response, particularly when modified using CSOA. In addition, the influence of the EV and UC in each region is investigated, and a sensitivity analysis is provided in order to demonstrate the resilience of the proposed controller in a variety of different situations. The CSOA and GA algorithms are used to compare the standard PID, PIDD, and FOPID controllers and the suggested FOPID + PFC controller for each and every case. The ISTSE objective function is used in order to compare the dynamic reactions. The fitness curve using CSOA-FOPID + PFC is shown in Fig. 23. The best ISTSE values using CSOA: FOPID + PFC, CSOA: FOPID + PFC with EV and UC are 3.11 × 10^–5^, and 1.39 × 10^–5^ respectively. Tables 2 and 3 provide, respectively, the optimal gains of the controllers in Area 1 and 2.

**Table 2:** Area 1 controllers optimal gains (T-G-S).

Sl. No	Controller	Area-1 subjected to 10% SLP							
		K_1_	K_2_	K_p1_	K_i1_	K_d1_	K_d11_	ʎ_1_	µ_1_
1	GA:PID	…	…	4.9983	4.9993	1.5462	…	…	…
2	CSOA:PID	…	…	3.012	3.004	2.998	…	…	…
3	CSOA:PIDD	…	…	10.001	10.011	2.6012	0.013	…	…
4	CSOA:FOPID	…	…	29.998	8.7544	29.999	…	− 0.99	1
5	CSOA:FOPID + PFC	12.6848	28.9854	29.9763	8.3228	29.999	…	− 0.98	0.991
6	CSOA:FOPID + PFC with EV and ESU	12.8486	28.4589	29.3679	8.8223	29.97	…	− 0.97	0.993

**Table 3:** Area 2 controllers optimal gains (T-G-S).

Sl. No	Controller	Area-2 subjected to 10% SLP							
		K_3_	K_4_	K_p2_	K_i2_	K_d2_	K_d22_	ʎ_2_	µ_2_
1	GA:PID	…	…	4.9969	3.7001	2.8817	…	….	…
2	CSOA:PID	….	…	0.011	0.7595	0.012		….	…
3	CSOA:PIDD	…	…	9.997	9.999	3.5215	4.199	……	…
4	CSOA:FOPID	…	…	22.5133	17.5362	29.97	…	− 0.658	0.997
5	CSOA:FOPID + PFC	12.6848	28.9854	12.6848	29.6402	29.98	….	− 0.657	0.992
6	CSOA:FOPID + PFC with EV and ESU	12.8468	28.4589	12.8486	29.2046	29.79	…	− 0.658	0.989

#### Poolco transaction (PT)

Under this arrangement, each GENCO is allowed to contribute in AGC according to their apfs, and each DISCO has contract with the GENCOs of same zone. As per the transaction policy, area 1 DISCOs cannot buy electricity from Area 2 GENCOs. All DISCOs and GENCOs have their contract as per DPM as given in (45). Eqs. (41) to (44) may be used to articulate the power generation by various GENCOs and the designated value of ΔP_tie_ for the systems.

41 $$\Delta P_{{Gni}} = \alpha _{{i1}} \Delta P_{{L1}} + \alpha _{{i2}} \Delta P_{{L2}} + \alpha _{{i3}} \Delta P_{{L3}} + \alpha _{{i4}} \Delta P_{{L4}} + apf_{i} \Delta P_{{Li,UC'}} ;i = 1,4$$

42 $$\Delta P_{{Ggi}} = \alpha _{{i1}} \Delta P_{{L1}} + \alpha _{{i2}} \Delta P_{{L2}} + \alpha _{{i3}} \Delta P_{{L3}} + \alpha _{{i4}} \Delta P_{{L4}} + apf_{i} \Delta P_{{Li,UC'}} ;i = 2,5$$

43 $$\Delta P_{{Gsi}} = \alpha _{{i1}} \Delta P_{{L1}} + \alpha _{{i2}} \Delta P_{{L2}} + \alpha _{{i3}} \Delta P_{{L3}} + \alpha _{{i4}} \Delta P_{{L4}} + apf_{i} \Delta P_{{Li,UC'}} ;i = 3,6$$

44 $$\begin{aligned} \Delta P_{{tie}}^{{Scheduled}} = & (\alpha _{{13}} + \alpha _{{23}} + \alpha _{{33}} )\Delta P_{{L3}} + (\alpha _{{14}} + \alpha _{{24}} + \alpha _{{34}} )\Delta P_{{L4}} \\ & - (\alpha _{{41}} + \alpha _{{51}} + \alpha _{{61}} )\Delta P_{{L1}} - (\alpha _{{42}} + \alpha _{{52}} + \alpha _{{62}} )\Delta P_{{L2}} \\ \end{aligned}$$

45 $${\text{DPM}} = \left( {\begin{array}{*{20}c} {0.3333} & {0.3333} & 0 & 0 \\ {0.3333} & {0.3333} & 0 & 0 \\ {0.3333} & {0.3333} & 0 & 0 \\ 0 & 0 & 0 & 0 \\ 0 & 0 & 0 & 0 \\ 0 & 0 & 0 & 0 \\ \end{array} } \right)$$

46 $${\text{DPM}} = \left( {\begin{array}{*{20}c} {0.2} & {0.1} & {0.3} & 0 \\ {0.2} & {0.2} & {0.1} & {0.1666} \\ {0.1} & {0.3} & {0.1} & {0.1666} \\ {0.2} & {0.1} & {0.1} & {0.3336} \\ {0.2} & {0.2} & {0.2} & {0.1666} \\ {0.1} & {0.1} & {0.2} & {0.1666} \\ \end{array} } \right)$$

**Fig. 24:**
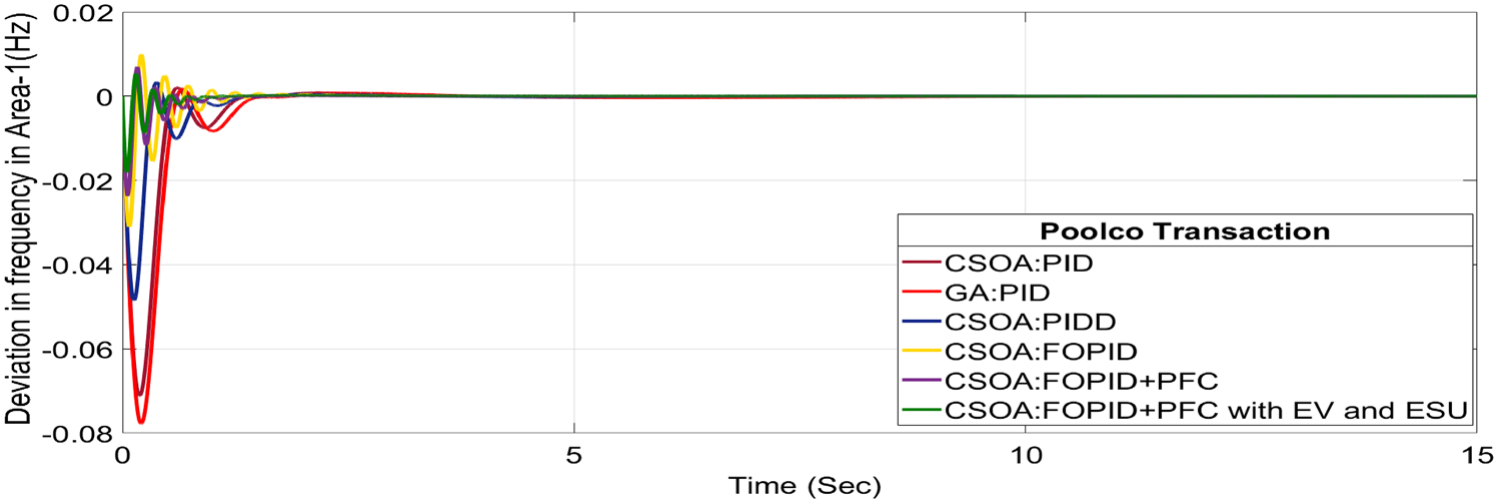
∆F_1_ reactions for PT.

**Fig. 25:**
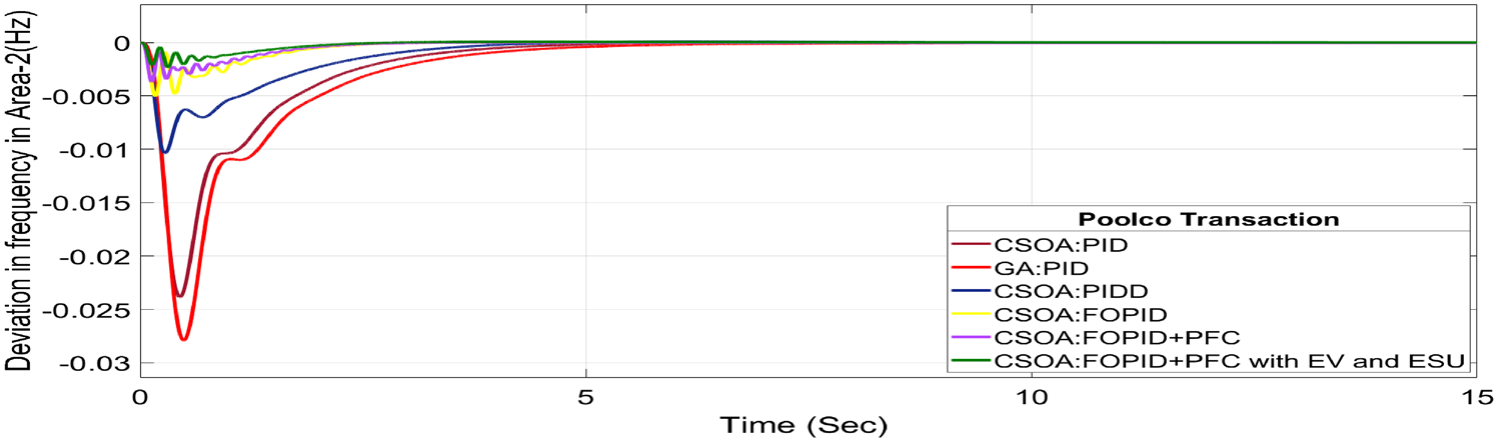
∆F_2_ reactions for PT.

**Fig. 26:**
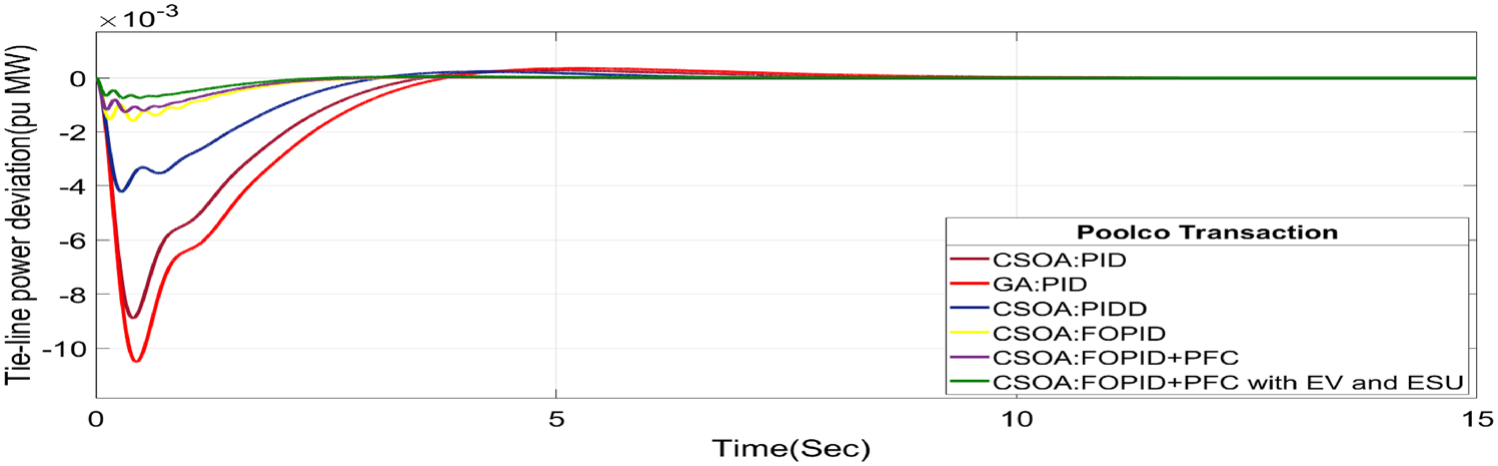
∆P_tie_ reactions for PT.

The rest of the DPM elements are zero since area 2, DISCOs do not need electricity from area 1 or 2 GENCOs. Figures 24, 25 and 26 illustrate dynamic reactions for the proposed system subjected to 10% SLP in each area under various control schemes, it can be concluded that EV and UC units boost the dynamic response of the system by rapidly settling the oscillations due to SLP. The convergence characteristics with hybrid CSOA based FOPID and polar fuzzy control scheme is shown in Fig. 5.1. It has best ISTSE value of 1.39 × 10^–5^. The dynamic reactions parameters of CSOA based FOPID and PFC with hybrid ESS for multiple power exchanges, as Table 6 illustrates. As per Table 6, the results reveal that the CSOA based FOPID and PFC with hybrid ESS produces the least amount of O_sh_ (ΔF_1_ = 0.0053 Hz, ΔF_2_ = 0 Hz, ΔP_tie_ = 0 p.u. MW), U_S_ (ΔF_1_ = − 0.0179 Hz, ΔF_2_ = − 0.0023 Hz, ΔP_tie_ = − 0.0007 pu MW), and rapidly get at zero in minimum T_S_ (ΔF_1_ = 0.878 s, ΔF_2_ = 2.228 s, ΔP_tie_ = 1.827 s) as compared to other controllers. Hence the dynamic reactions obtained from CSOA based FOPID and PFC with hybrid ESS for the proposed system is found superior as compared to CSOA-FOPID + PFC without hybrid ESS, CSOA- PI^ʎ^D^µ^, CSOA-PIDD, CSOA-PID, and GA-PID controllers.

#### Bilateral transaction (BT)

**Fig. 27:**
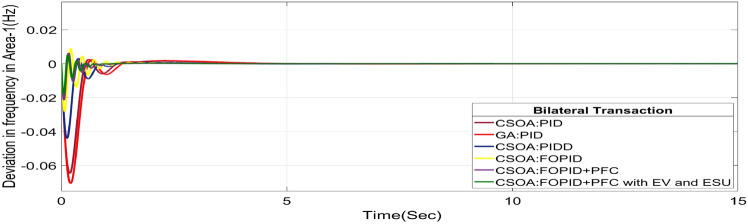
∆F_1_ reactions for BT.

**Fig. 28:**
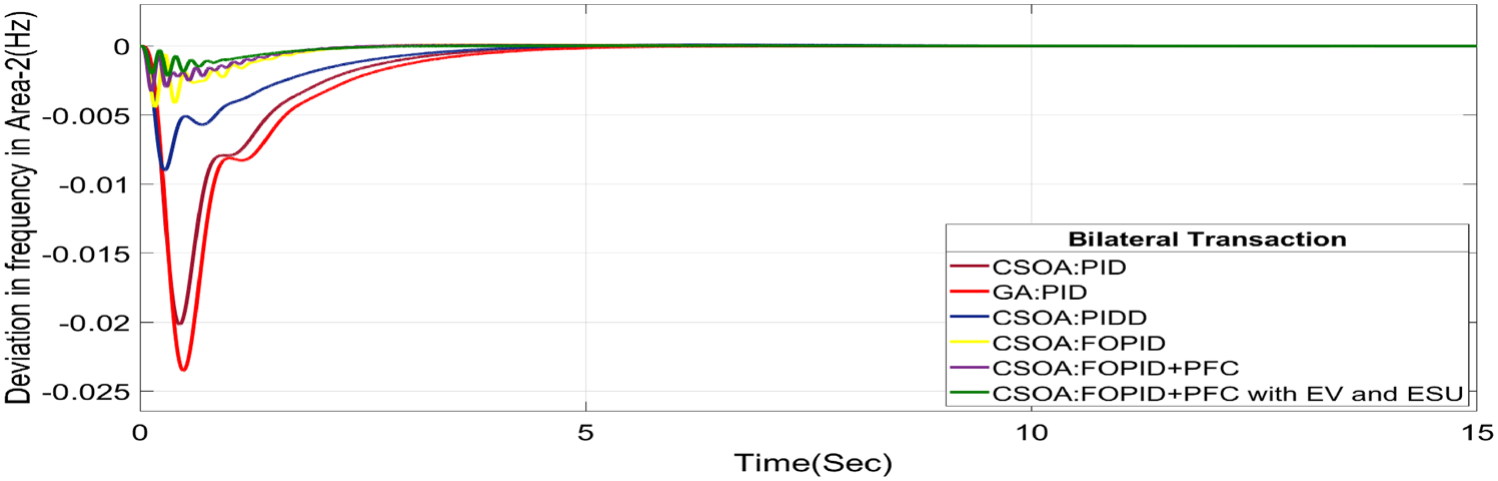
∆F_2_ reactions for BT.

**Fig. 29:**
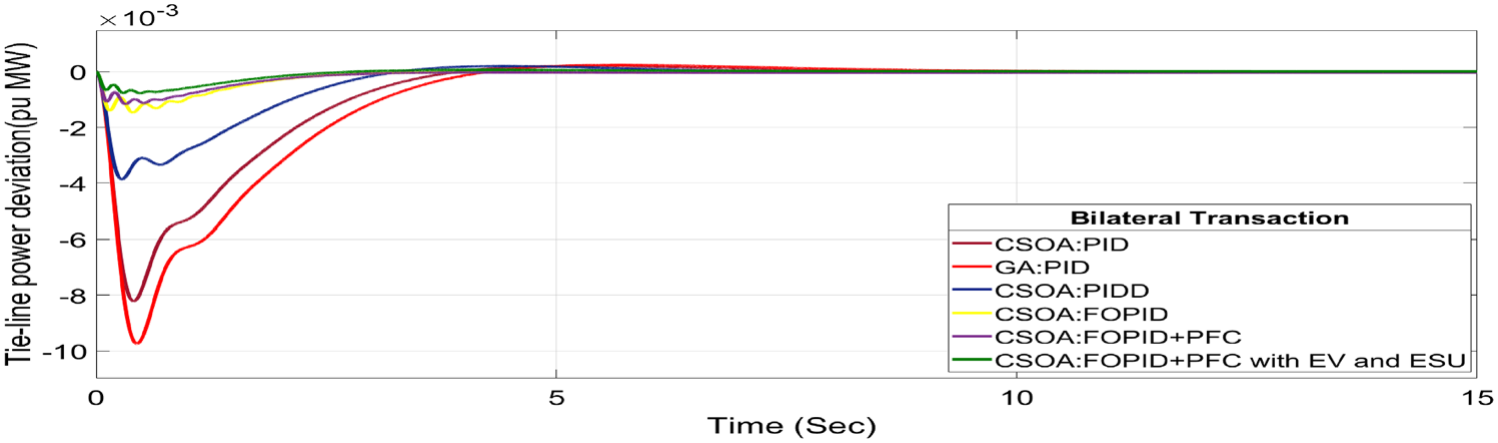
∆P_tie_ reactions for BT.

The BT includes the contract of transactions between a GENCO and a DISCO in the same area and any other location. According to Eq. (46), each DISCO and GENCO in the system constitutes its own contract as the DPM i.e. this agreement allows energy distribution businesses (DISCOs) in a certain area to negotiate with GENCOs from any region, whether they are nearby or not. Both parties accept contract terms. DISCO-4 does not need power from GENCO1. Hence the element of DPM is 0. Each GENCO assigns pu MW to each apf as described above. The simulated responses for proposed system are depicted in Figs. 27, 28 and 29. These responses are shown for a variety of control strategies for BT. The steady-state value obtained from the Eq. (42) is 0 p.u. MW, which agrees with the findings of the simulation. As per Table 6, the results reveal that the CSOA based FOPID and PFC with EV and ESU produces the least amount of O_sh_ (ΔF_1_ = 0.0052 Hz, ΔF_2_ = 0 Hz, ΔP_tie_ = 0 p.u. MW), U_S_ (ΔF_1_ = − 0.0187 Hz, ΔF_2_ = − 0.0021 Hz, ΔP_tie_ = − 0.0007 pu MW), and rapidly get at zero in minimum T_S_ (ΔF_1_ = 1.037 s, ΔF_2_ = 2.006 s, ΔP_tie_ = 1.945 s) as compared to other controllers. The dynamic reactions of hybrid CSOA based FOPID and PFC scheme with ESUs is found superior as compared to other control schemes i.e. CSOA-FOPID + PFC without ESUs, CSOA- PI^ʎ^D^µ^, CSOA-PIDD, CSOA-PID, and GA-PID controllers.

#### Contract violation (CV)

**Fig. 30:**
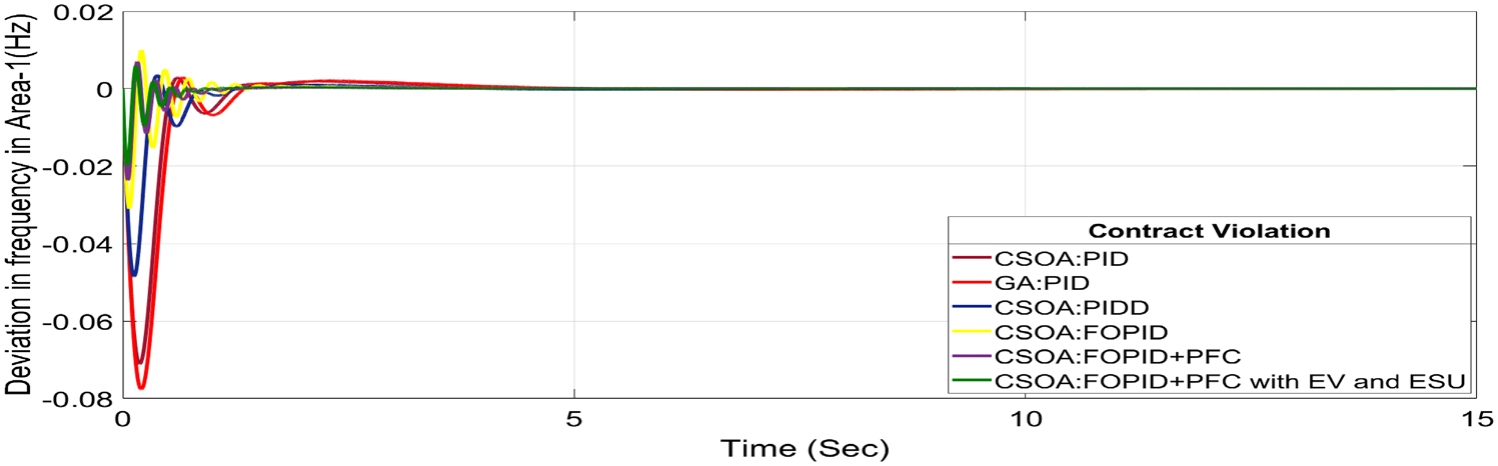
∆F_1_ reactions for CV.

**Fig. 31:**
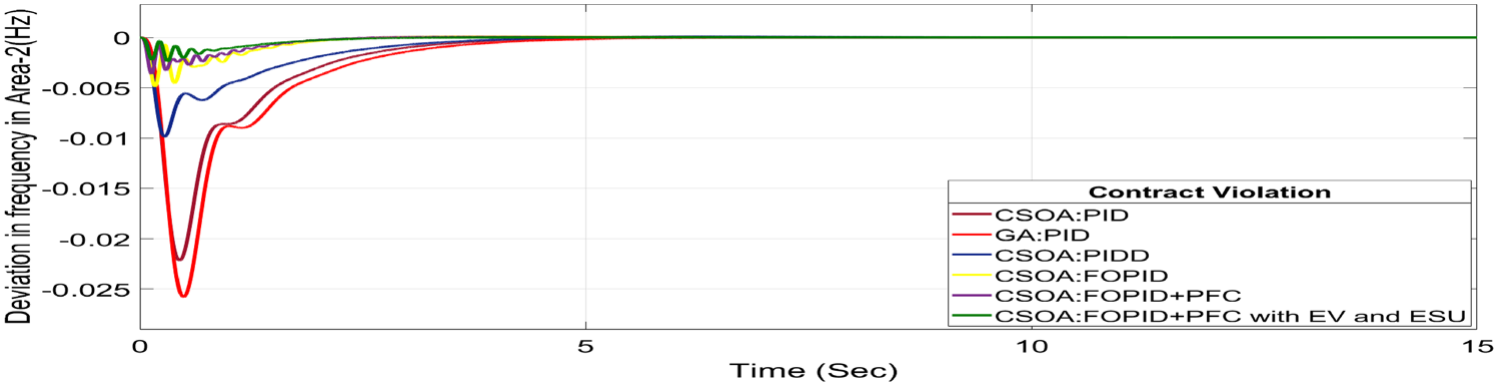
∆F_2_ reactions for CV.

**Fig. 32:**
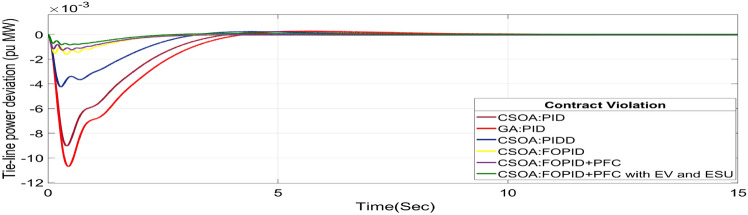
∆P_tie_ reactions for CV.

A DISCO may sometimes violate the terms of the contract arrangement when things are terrible. In this scenario, GENCOs could need to provide the DISCO with more power than what is specified in the contract. The GENCOs belonging to each area shall fulfil the DISCO’s request for un-contracted extra power. Consider a contact violation scenario where DISCO-1 requested 0.0015 p.u. MW of un-contracted electricity from the local GENCOs and DISCO-2 requested 0.0015 p.u. MW of un-contracted electricity from the local GENCOs. Due to the increased need for electricity, area 1’s total load request has raised to 0.003 pu MW, i.e. new power requests in area 1 indicate 0.003 pu MW. Figures 30, 31 and 32 show dynamic curves for CV. Frequency variations are greater in zones and tie-line power deviation responses have become worse when DISCOs break the conditions of the energy contract. As per Table 4, the results reveal that the CSOA based FOPID and PFC with EV and ESU produces the least amount of O_sh_ (ΔF_1_ = 0.0059 Hz, ΔF_2_ = 0 Hz, ΔP_tie_ = 0 pu MW), U_S_ (ΔF_1_ = − 0.0195 Hz, ΔF_2_ = − 0.0024 Hz, ΔP_tie_ = − 0.0008 pu MW), and rapidly get at zero in minimum T_S_ (ΔF_1_ = 1.037 s, ΔF_2_ = 2.006 s, ΔP_tie_ = 1.945 s) as compared to other controllers. The dynamic response of hybrid CSOA based FOPID and polar fuzzy control scheme with EV and ESU is found superior as compared to other control schemes.

**Table 4:** System dynamic specs with CSOA: FOPID + PFC with EV and ESU.

Controller	Parameter	Overshoot (O_sh_)	Undershoot (U_sh_)	Settling Time (T_s_)	Overshoot (O_sh_)	Undershoot (U_sh_)	Settling Time (T_s_)	Overshoot (Os_h_)	Undershoot (U_sh_)	Settling Time (T_s_)
		PT			BT			CV		
GA:PID	∆ F_1_	0.0015	− 0.0703	3.695	0.0024	− 0.0705	4.975	0.0027	− 0.0771	5.025
	∆ F_2_	0	− 0.0253	6.174	0	− 0.0235	5.038	0	− 0.0257	5.218
	∆P_tie_	0.0003	− 0.0095	10.573	0.0002	− 0.0097	7.687	0.0002	− 0.0107	7.795
CSOA:PID	∆ F_1_	0.0019	− 0.0645	3.557	0.0024	− 0.0646	4.43	0.0027	− 0.071	4.539
	∆ F_2_	0	− 0.0216	5.008	0	− 0.0202	4.53	0	− 0.0221	4.59
	∆P_tie_	0.0002	− 0.0081	6.505	0.0002	− 0.0082	6.574	0.0002	− 0.009	6.308
CSOA:PIDD	∆ F_1_	0.0029	− 0.0437	2.951	0.0029	− 0.0437	3.357	0.0034	− 0.0482	3.775
	∆ F_2_	0	− 0.0094	4.081	0	− 0.0089	4.161	0	− 0.0097	4.379
	∆P_tie_	0.0002	− 0.0038	5.097	0.0002	− 0.0038	5.422	0.0002	− 0.0042	5.747
CSOA:FOPID	∆ F_1_	0.0089	− 0.0282	1.414	0.0089	− 0.0282	3.12	0.0099	− 0.0304	3.269
	∆ F_2_	0	− 0.0045	2.517	0	− 0.0044	2.257	0	− 0.0048	2.327
	∆P_tie_	0	− 0.0014	2.142	0	− 0.0014	2.497	0	− 0.0016	2.556
CSOA:FOPID + PFC	∆ F_1_	0.006	− 0.0211	1.235	0.0063	− 0.0211	1.275	0.0069	− 0.0232	1.543
	∆ F_2_	0	− 0.0033	2.427	0	− 0.0032	2.128	0	− 0.0035	2.148
	∆P_tie_	0	− 0.0011	2.063	0	− 0.0011	2.349	0	− 0.0012	2.388
CSOA:FOPID + PFC with EV and ESU	∆ F_1_	0.0053	− 0.0179	0.878	0.0052	− 0.0187	1.037	0.0059	− 0.0195	1.126
	∆ F_2_	0	− 0.0023	2.228	0	− 0.0021	2.006	0	− 0.0024	2.018
	∆P_tie_	0	− 0.0007	1.827	0	− 0.0007	1.945	0	− 0.0008	2.191

#### Sensitivity analysis of combined FOPID + PFC

##### Considering RLD

**Fig. 33:**
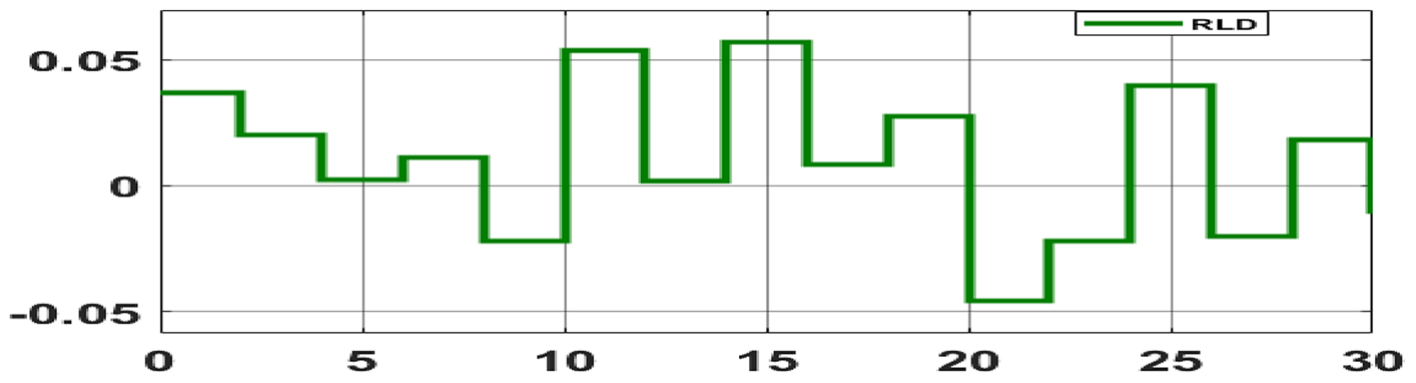
Random load demand (RLD).

**Fig. 34:**
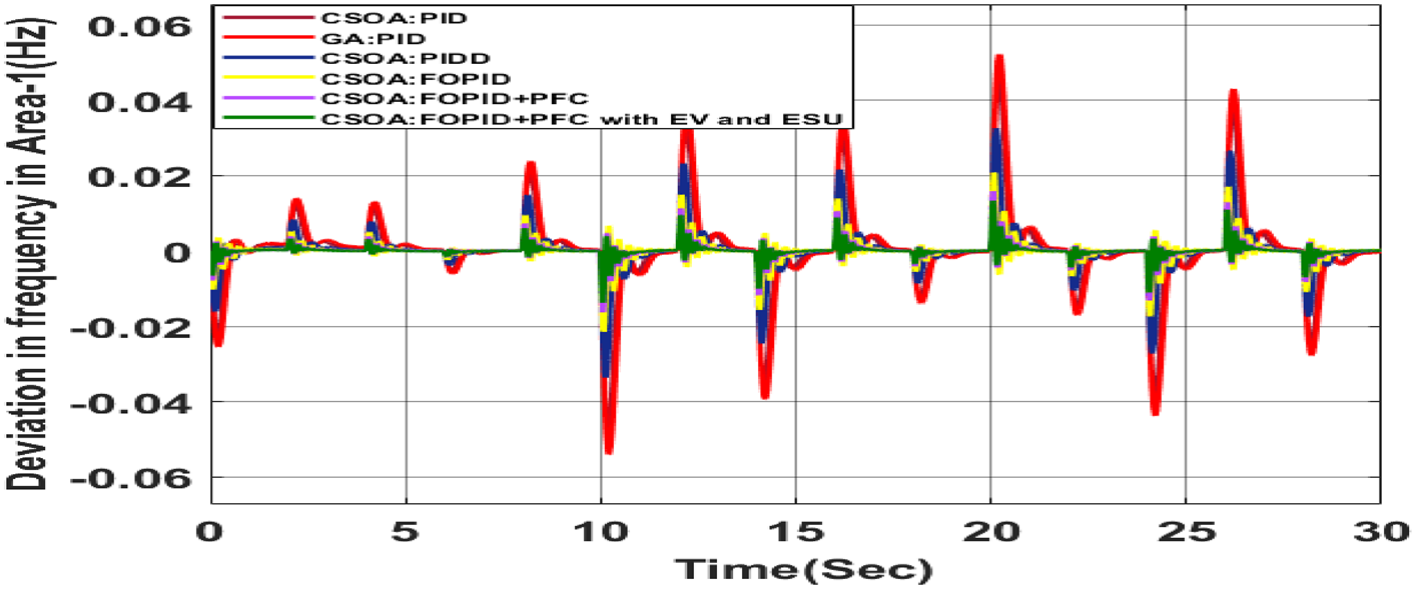
∆F_1_ reactions for CV with RLD.

**Fig. 35:**
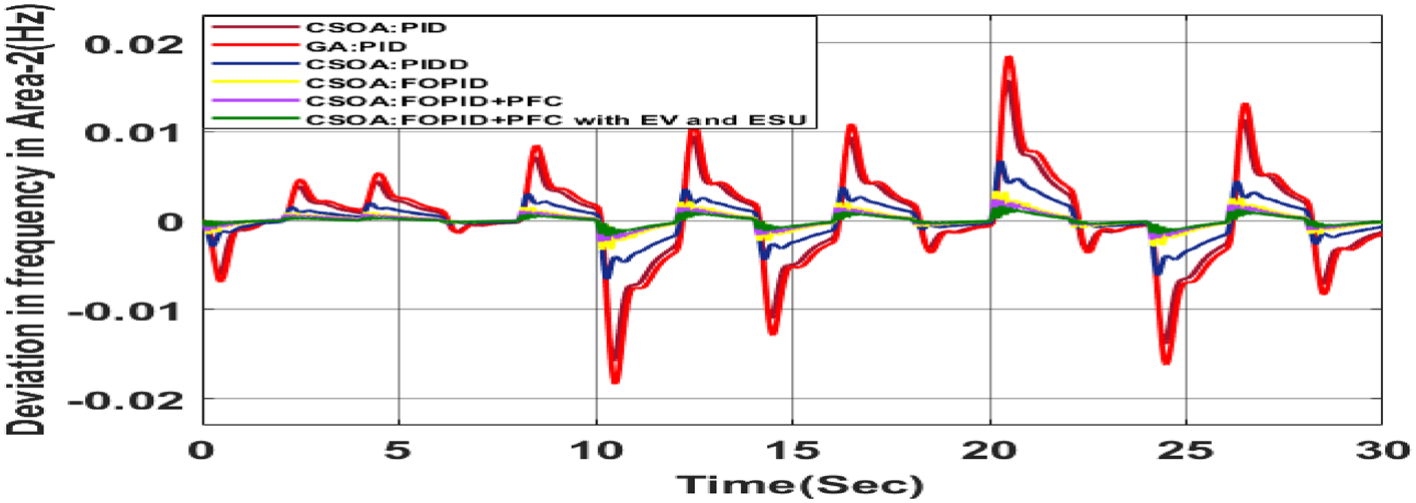
∆F_2_ reactions for CV with RLD.

**Fig. 36:**
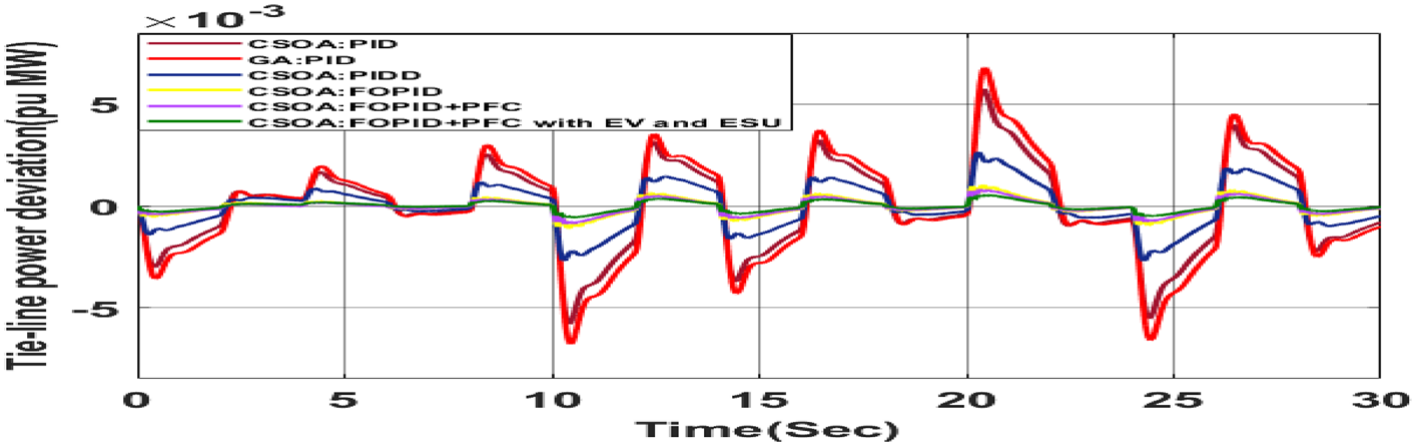
∆P_tie_ reactions for CV with RLD.

The random load demand (RLD) is considered for 30 s as shown in Fig. 33.The performance of proposed hybrid FOPID + PFC with EV and ESU is carried out for CV as shown in Figs. 34, 35 and 36 against FOPID + PFC, FOPID, PIDD, PID controllers after obtaining parameters via CSOA and GA-PID controller. The observation of the dynamics reveals that with the proposed CSOA based hybrid FOPID + PFC, the overshoots (O_sh_) and undershoots (U_S_) and time of settling are minimum as compared to other controllers as mentioned above which shows superiority of hybrid FOPID + PFC with EV + ESU. The observed dynamics indicate that the controller can manage the previously specified real-world scenario.

##### Considering parameter uncertainty

**Fig. 37:**
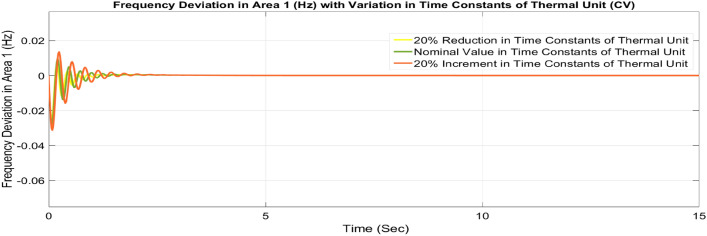
∆F_1_ reactions for CV.

**Fig. 38:**
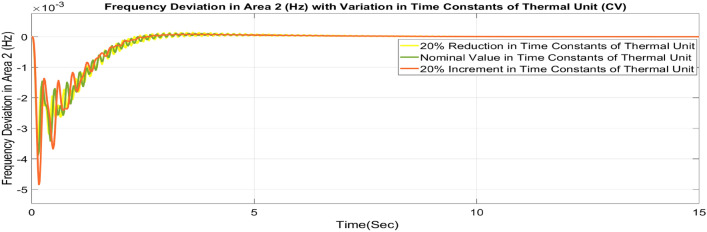
∆F_2_ reactions for CV.

**Fig. 39:**
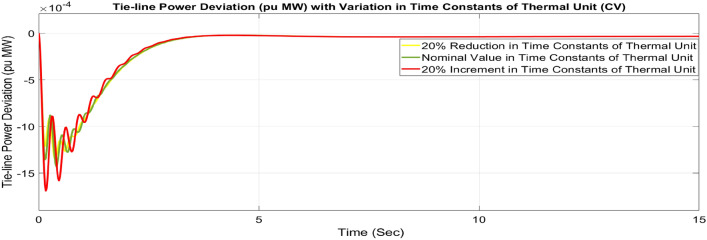
∆P_tie_ reactions for CV.

**Fig. 40:**
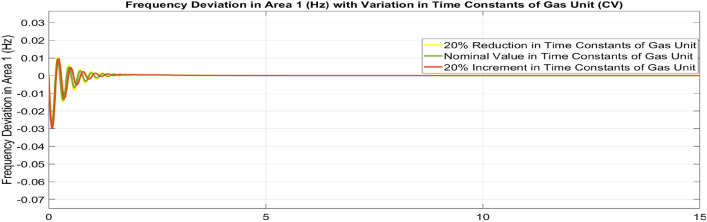
∆F_1_ reactions for CV.

**Fig. 41:**
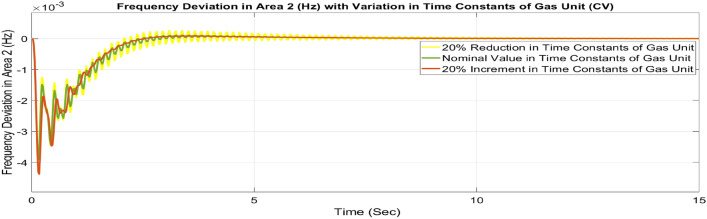
∆F_2_ reactions for CV.

**Fig. 42:**
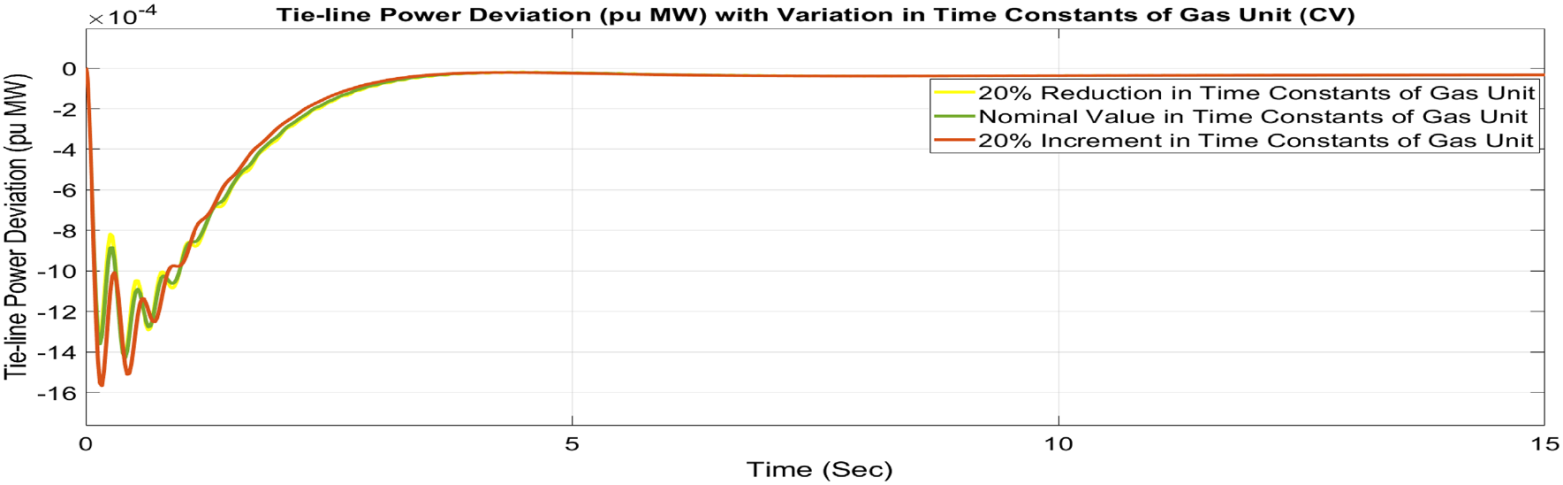
∆P_tie_ reactions for CV.

**Fig. 43:**
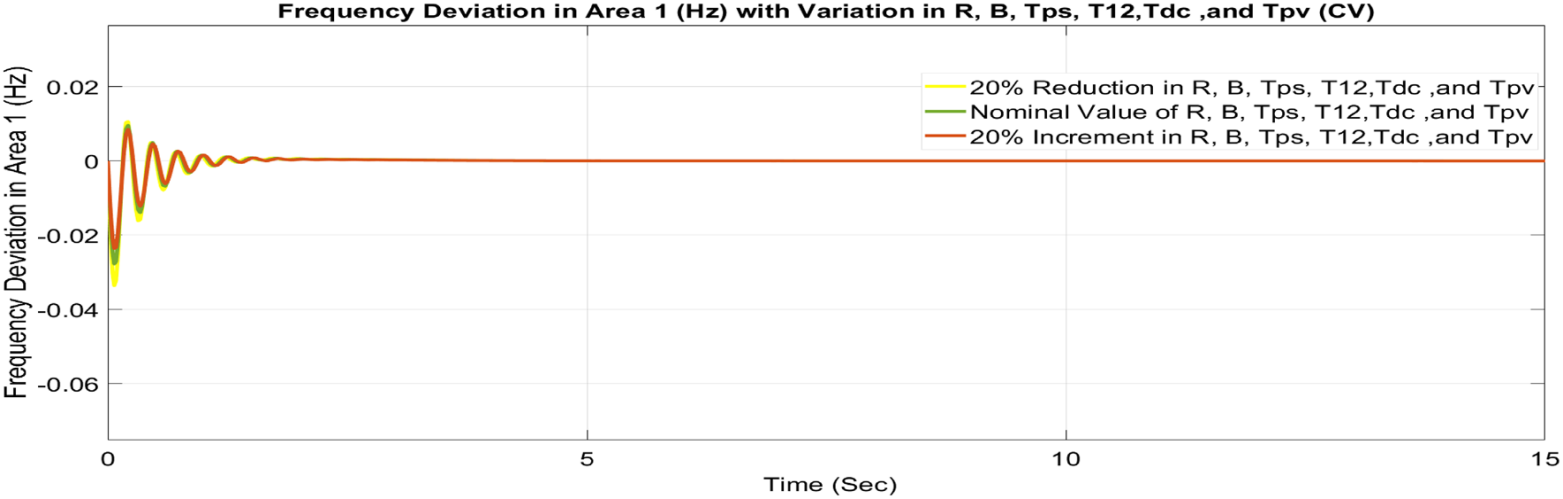
∆F_1_ reactions for CV.

**Fig. 44:**
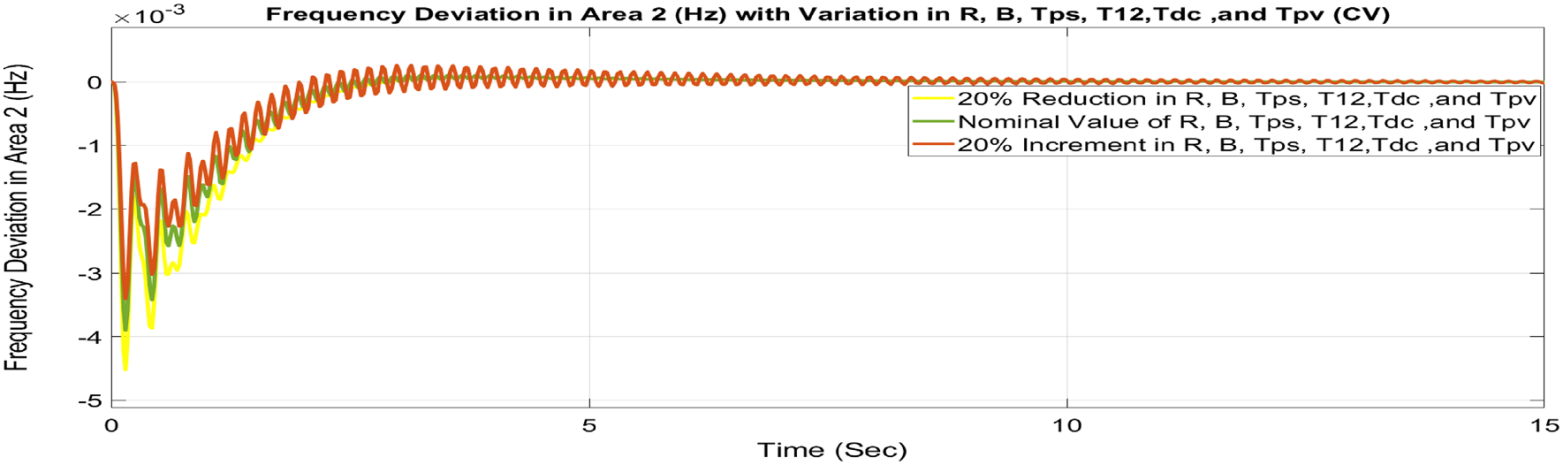
∆F_2_ reactions for CV.

**Fig. 45:**
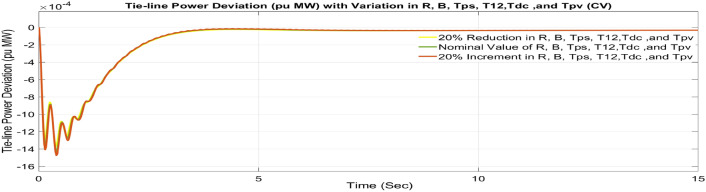
∆P_tie_ reactions for CV.

Proposed CSOA based FOPID + PFC is designed using nominal value of configuration settings. However, these are not static in real-world scenarios. There may be some variations in the system parameter values due to operating conditions, aging effect, and errors in the measurement or due to reduction of the mathematical model’s assumptions. Thus in presence of system parameters variations, the system’s dynamic ability can decline and become subpar. In view of this, a decrease and increase in basic parameters of the system by 20% as shown in Table 5. The system parameters namely T_g_, and T_t_ are varied simultaneously by 20% in the wake of 10% SLD in each control zone. Figures 37, 38 and 39 display the system’s dynamic reactions for ∆F_1_, ∆F_2_, and ∆P_tie_. Figures 40, 41 and 42 display the system’s dynamic reactions to changes in the time constant of the gas unit, as seen in ∆F_1_, ∆F_2_, and ∆P_tie_. Another parameters, namely R, B, T_ps_, T_12_,T_dc_, and T_PV_ are varied simultaneously by 20% in the wake of 10% SLD in each control zone. The dynamic responses of ∆F_1_, ∆F_2_, and ∆P_tie_ of the system are shown in Figs. 43, 44 and 45. It is found that CSOA based FOPID + PFC is quite robust to meet the AGC objectives.

**Table 5:** The variations in system parameters.

Sl. No	Parameter	Nominal Value	20% Reduct-ion	20% Increment
1	T_g_	0.08	0.064	0.096
2	T_t_	0.3	0.24	0.36
3	Xg	0.6	0.48	0.72
4	Yg	1	0.8	1.2
5	b_g_,c_g_	0.05,1	0.04,0.8	0.06,1.2
6	T_F_	0.23	0.184	0.276
7	T_CR_	0.01	0.008	0.012
8	T_CD_	0.2	0.16	0.24
9	1/R_11_, 1/R_12_, 1/R_13_	0.417, 0.417,0.417	0.334, 0.334,0.334	0.5004, 0.5004,0.5004
10	1/R_21_, 1/R_22_, 1/R_23_	0.417, 0.417,0.417	0.334, 0.334,0.334	0.5004, 0.5004,0.5004
11	B_1_,B_2_	0.425,0.425	0.34, 0.34	0.51,0.51
12	T_ps_	20	16	24
13	2πT_12_	0.544	0.4353	0.6528
14	T_dc_	0.2	0.16	0.24
15	T_PV_	1.8	1.44	2.16

##### Considering CTD

**Fig. 46:**
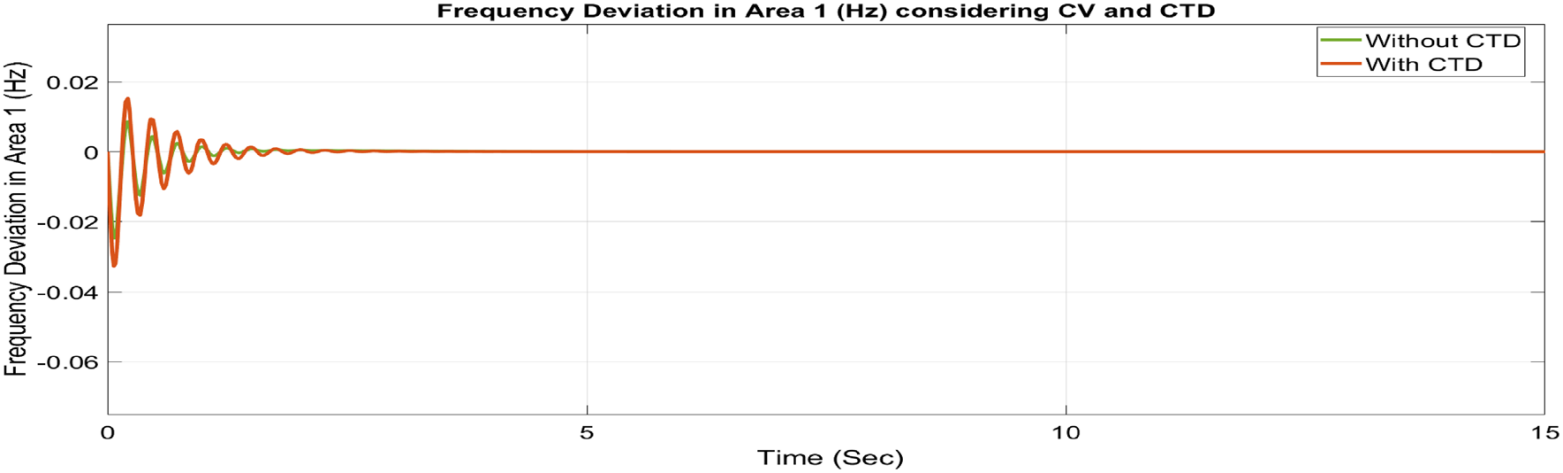
∆F_1_ reactions for CV and CTD.

**Fig. 47:**
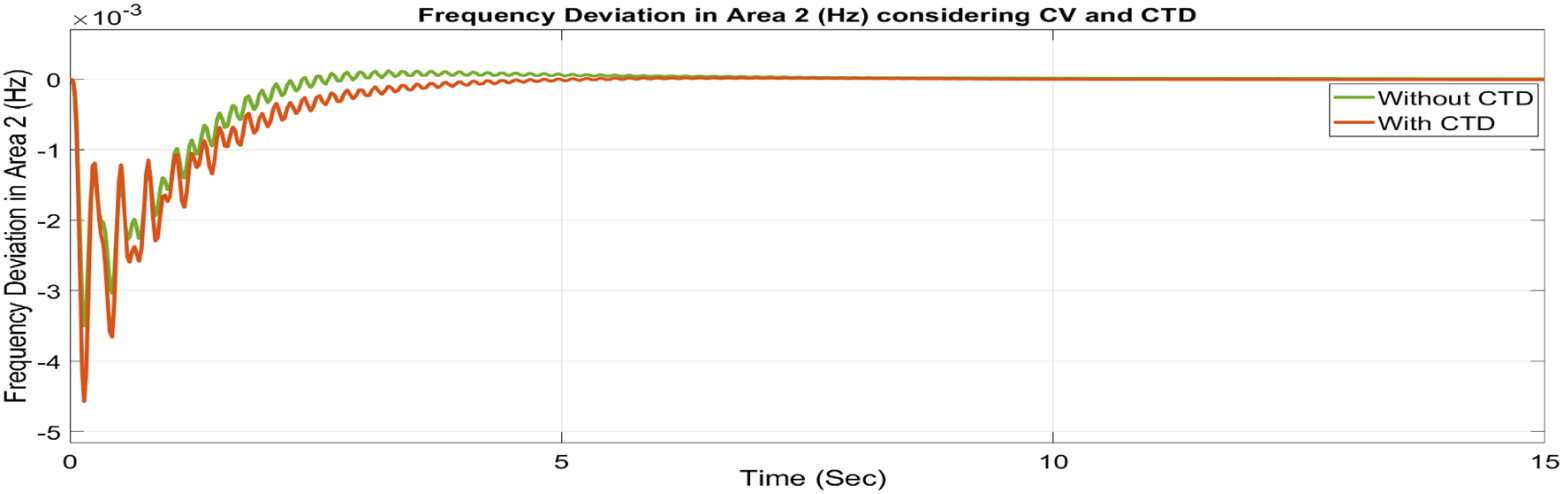
∆F_2_ reactions for CV and CTD.

**Fig. 48:**
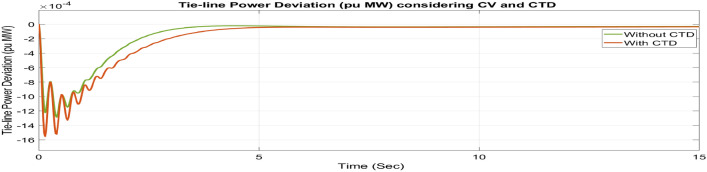
∆P_tie_ reactions for CV and CTD.

Many phase measuring units (PMUs) are installed in contemporary power systems to facilitate communication between different centers and areas. Transmission and generating systems often send a large number of signals to control centers, which then forward those signals to the generating stations. Signal transmission and reception between these centers and stations may affect the stability of the system. Since the secondary controller, which is the brain behind AGC, develops the command control signal using the ACE signal as input, these CTDs may cause delays in the input signals to the controllers, resulting in delays in the production of the command control signal. Therefore, a larger mismatch between generation and demand may result from delays in adjusting the generator’s operational set points. It might affect the system’s stability. Therefore, to avoid system instability, CTDs must be considered. Thus, as seen in Figures 46, 47 and 48, the impact of CTDs on the reorganized T-G-S system is considered here. The Taylor series in this research represents the transport delay, which is the communication delay, e^–sτd^ ^37^.

#### Feasibility of combined FOPID + PFC for restructured N-G-S system

**Fig. 49:**
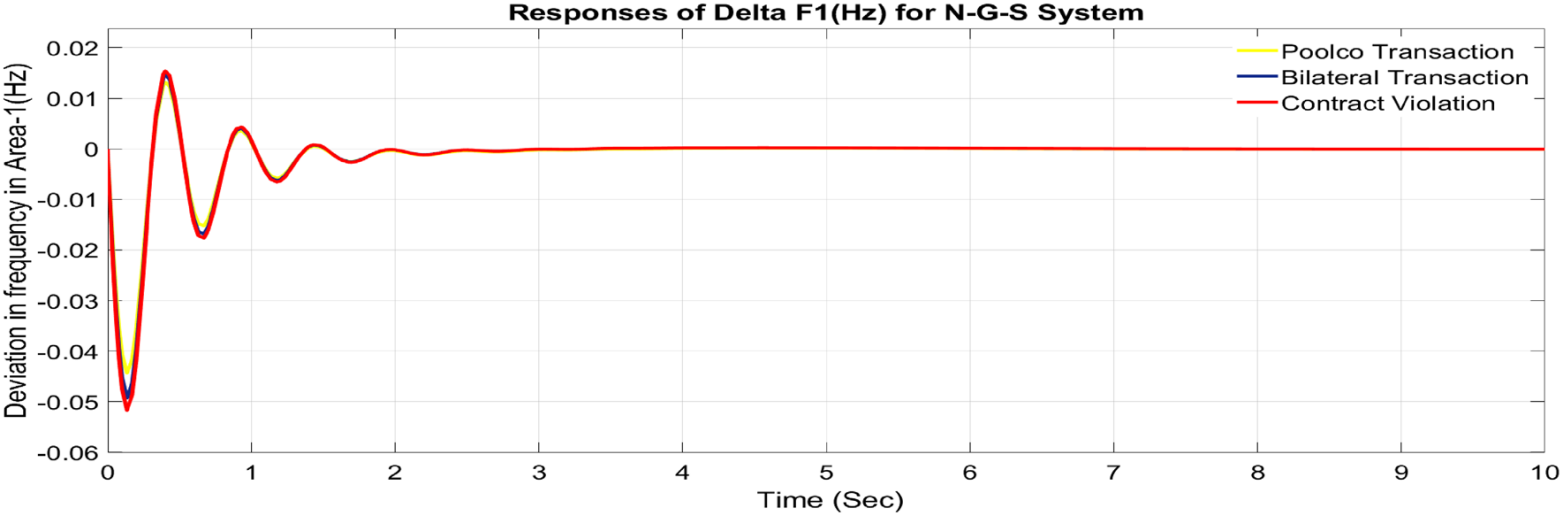
∆F_1_ reactions for N-G-S system.

**Fig. 50:**
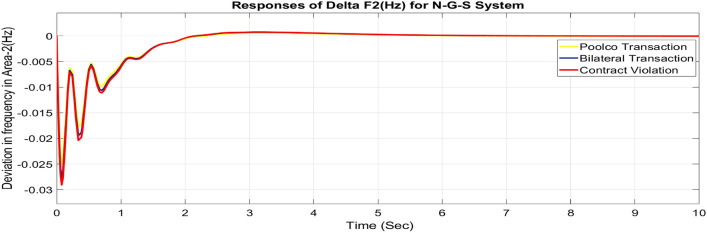
∆F_2_ reactions for N-G-S system.

**Fig. 51:**
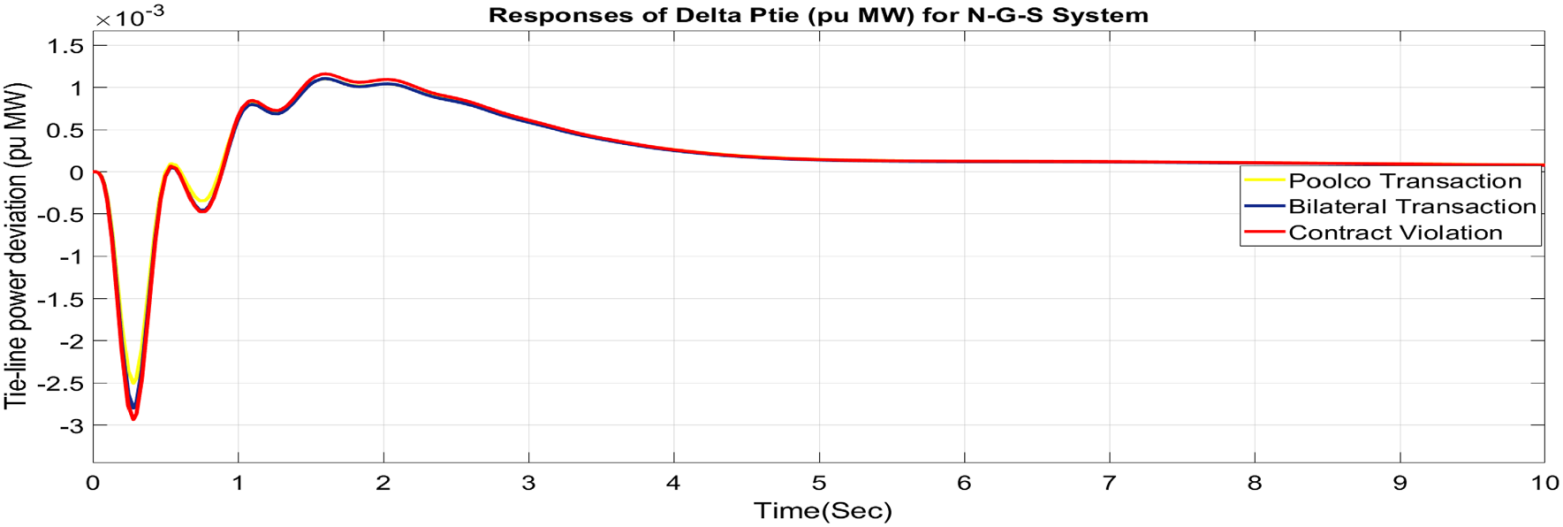
∆P_tie_ reactions for N-G-S system.

Utilizing the same optimized parameters used for the restructured T-G-S system, this is done to assess whether the proposed controller, a combination of FOPID and PFC, is practical for the restructured N-G-S system. The dynamic reactions that occur for ΔF_1_, ΔF_2_, and ΔP_tie_ when they are exposed to varied power transactions are shown graphically in Figs. 49, 50 and 51. Changes in frequency are more noticeable in zones where DISCOs breach the conditions of the energy contract, and the tie-line power deviation responses have become more significant as a result of these violations. Nevertheless, the proposed controller is capable of achieving the objectives of the AGC.

### The performance of CSOA based PII^ʎ^DD^µ^ controller with hybrid ESS

**Fig. 52:**
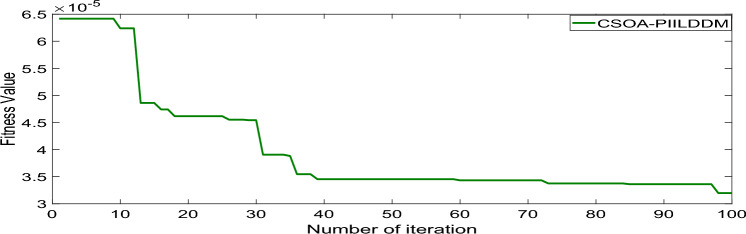
Convergence characteristics with CSOA: PII^ʎ^DD^µ^.

**Fig. 53:**
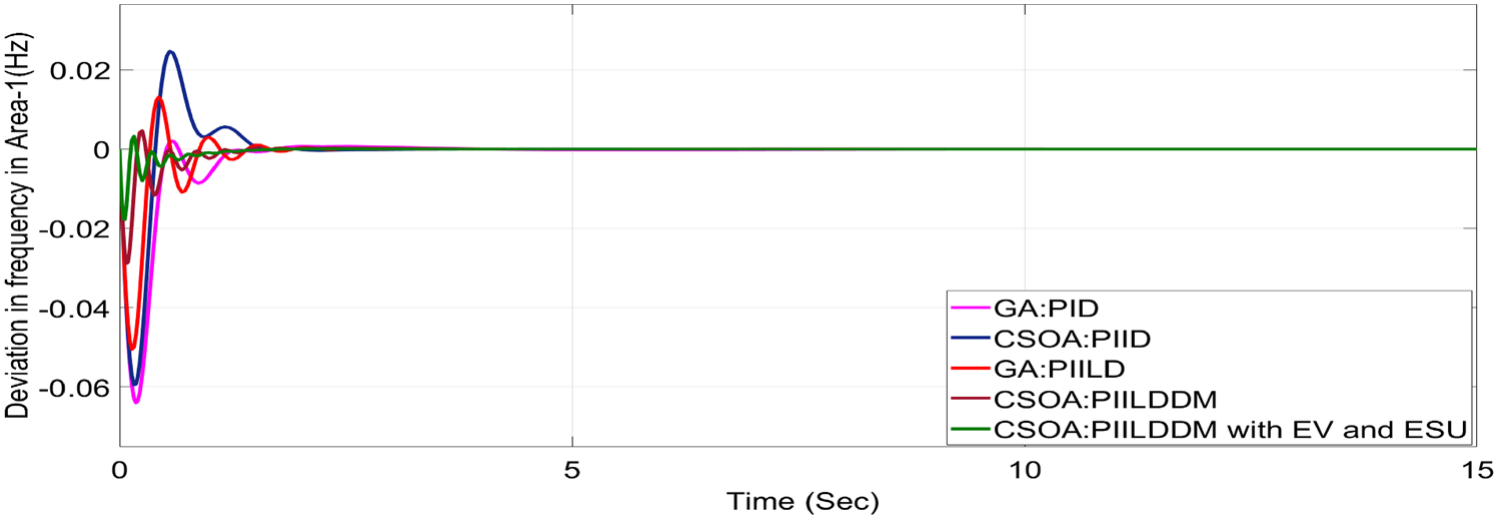
∆F_1_ reactions for PT.

**Fig. 54:**
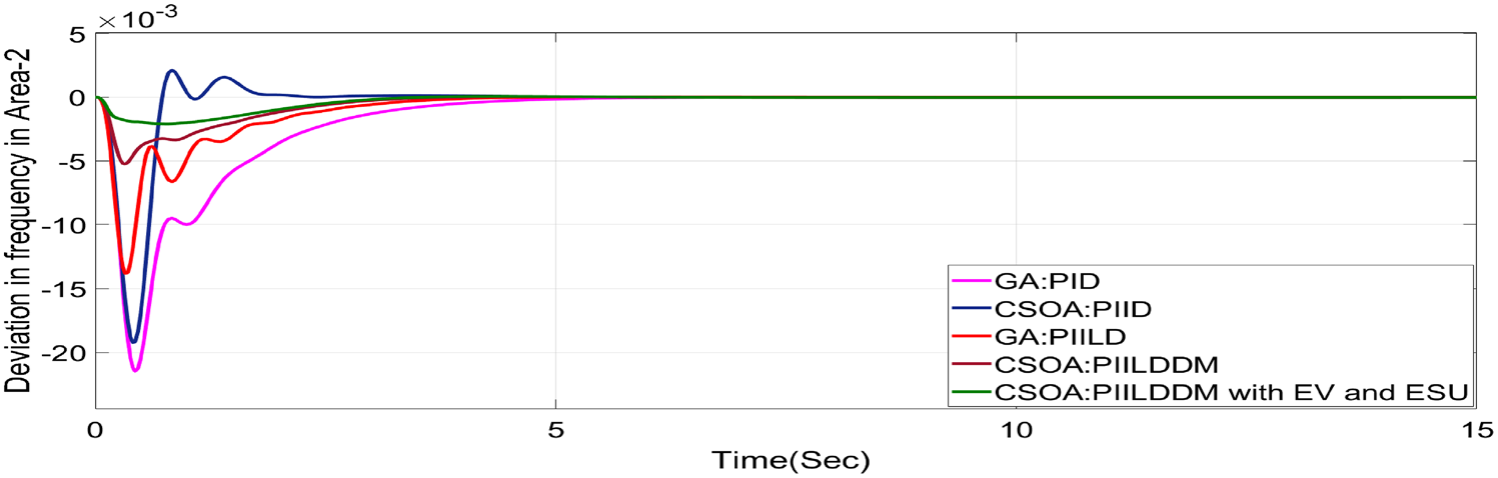
∆F_2_ reactions for PT.

**Fig. 55:**
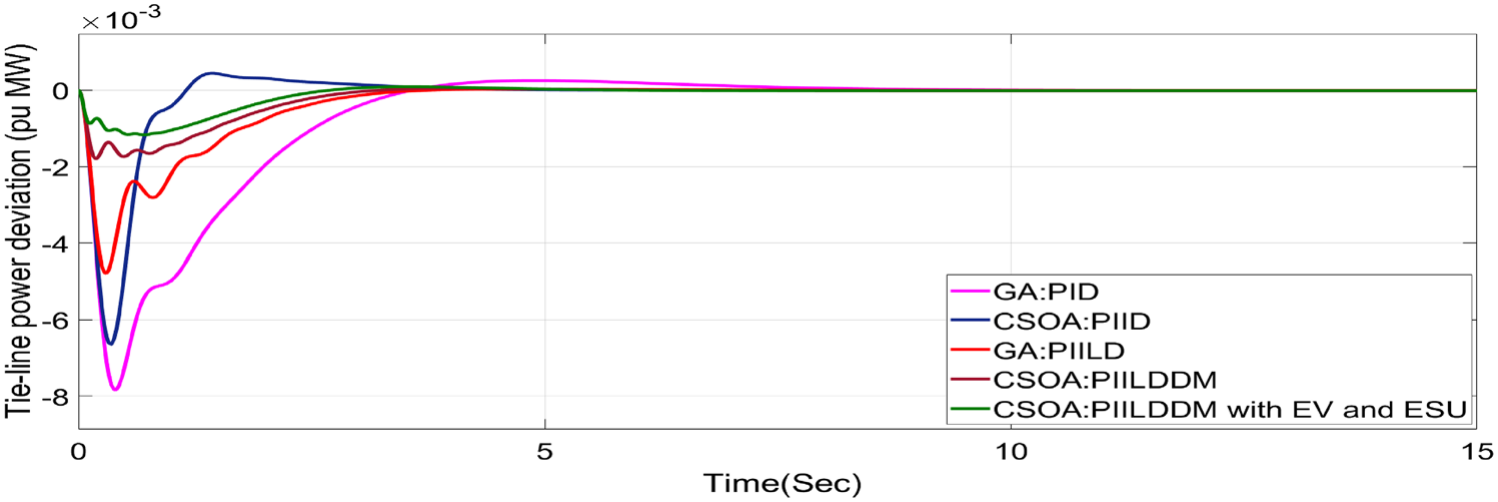
∆P_tie_ reactions for PT.

**Fig. 56:**
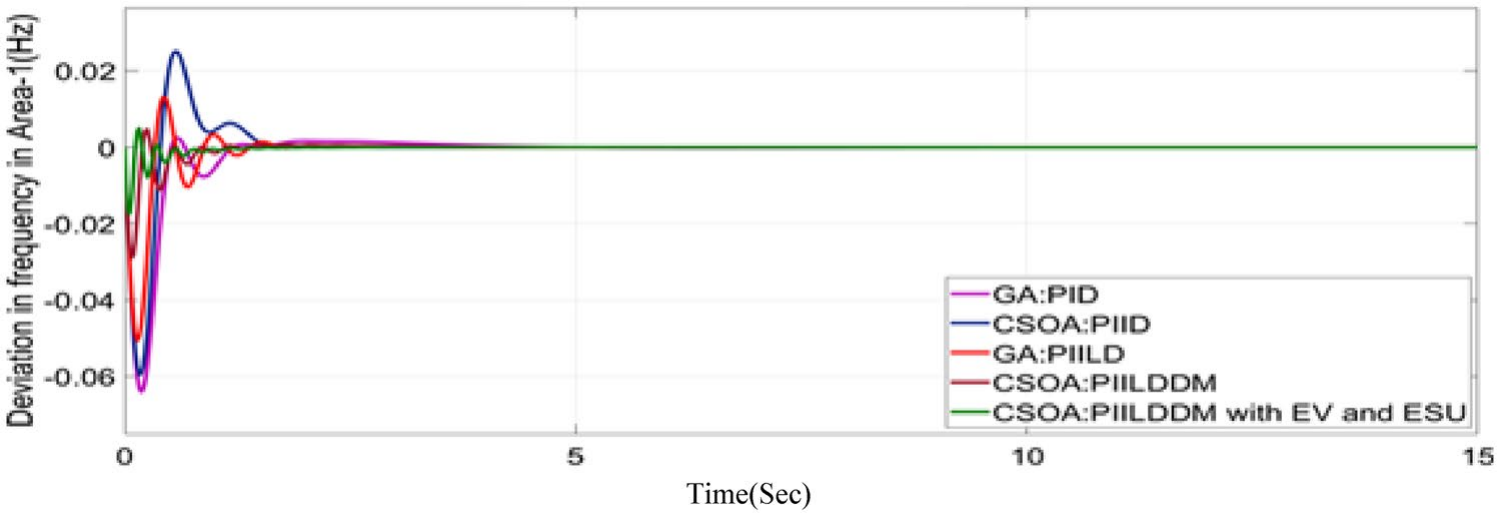
∆F_1_ reactions for BT.

**Fig. 57:**
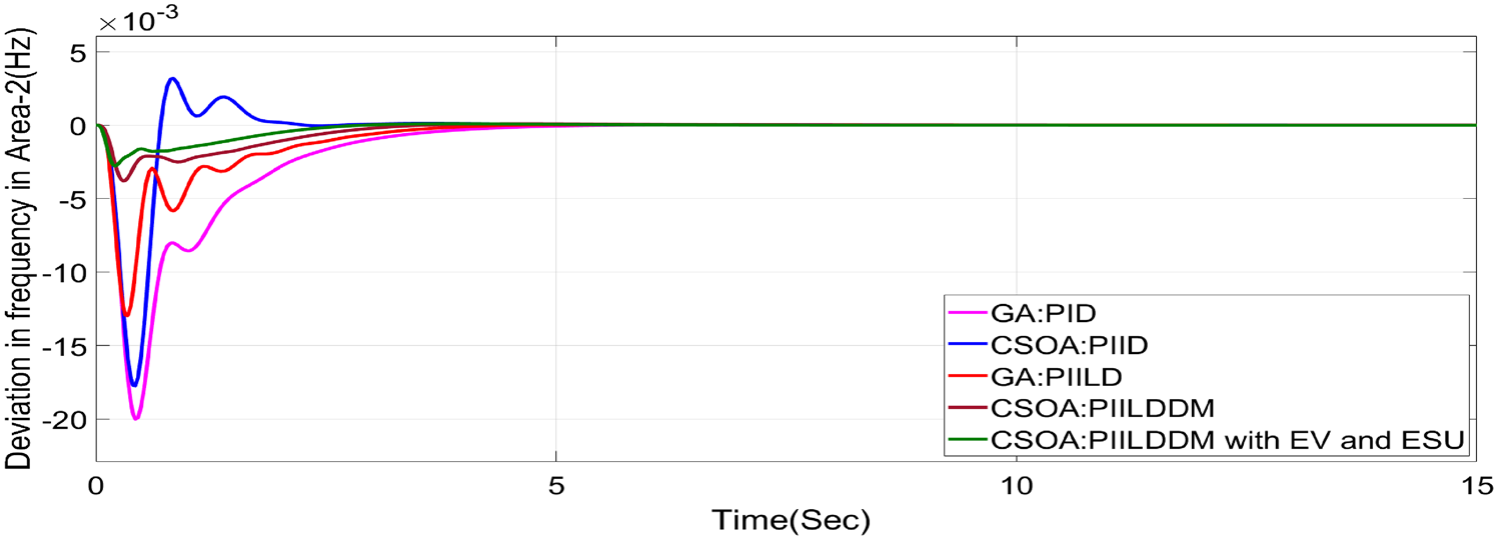
∆F_2_ reactions for BT.

**Fig. 58:**
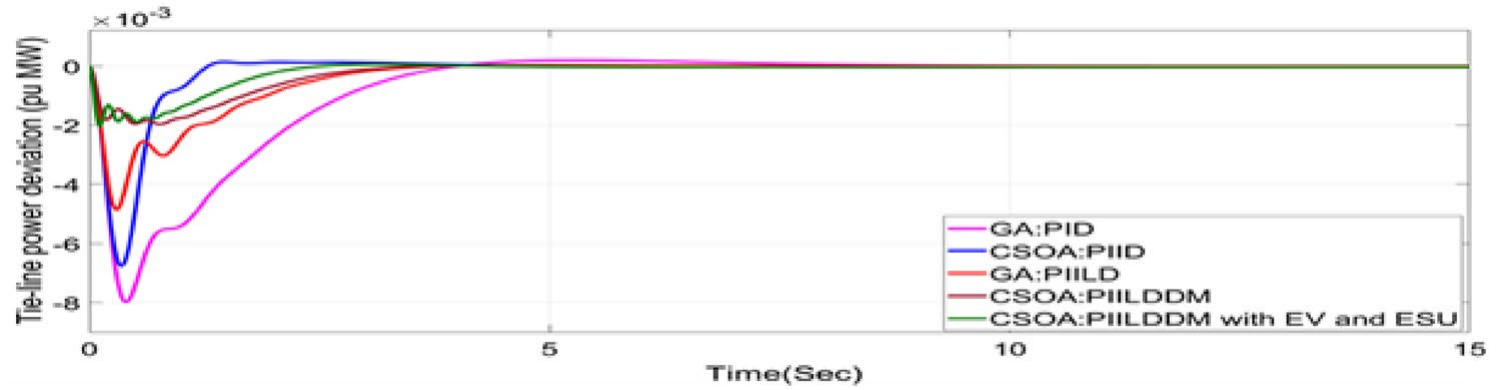
∆P_tie_ reactions for BT.

**Fig. 59:**
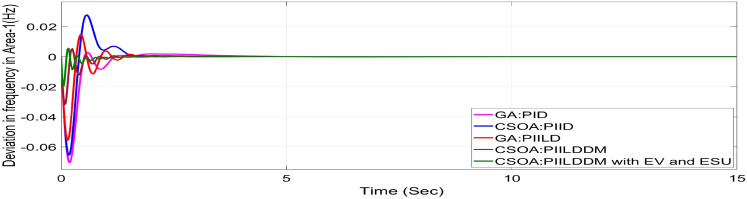
∆F_1_ reactions for CV.

**Fig. 60:**
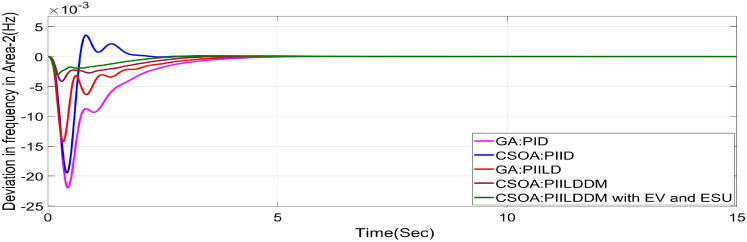
∆F_2_ reactions for CV.

**Fig. 61:**
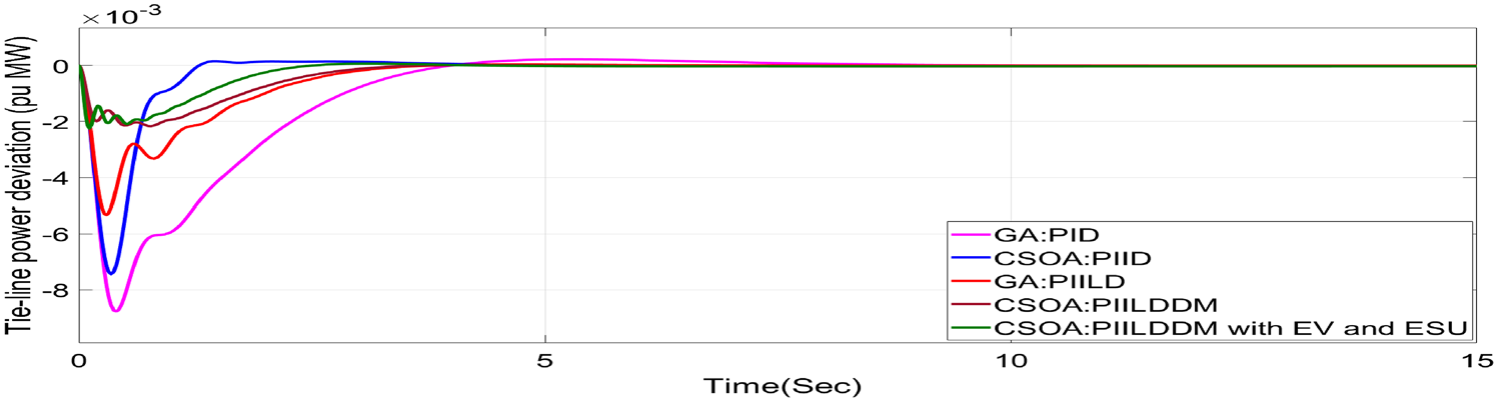
∆P_tie_ reactions for CV.

**Fig. 62:**
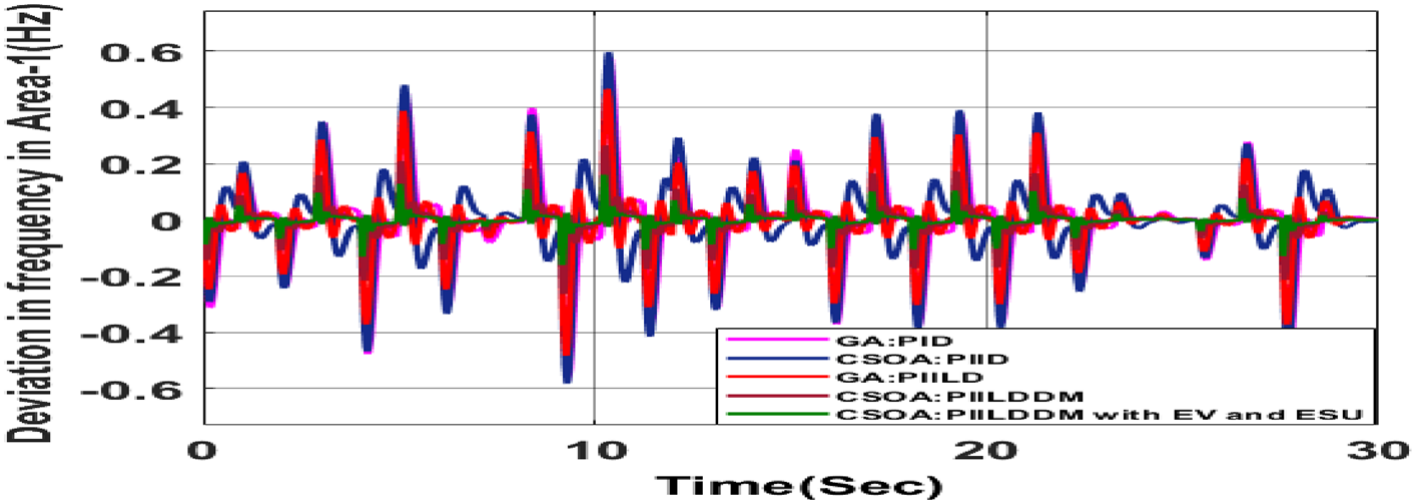
∆F_1_ reactions for CV with RLD.

**Fig. 63:**
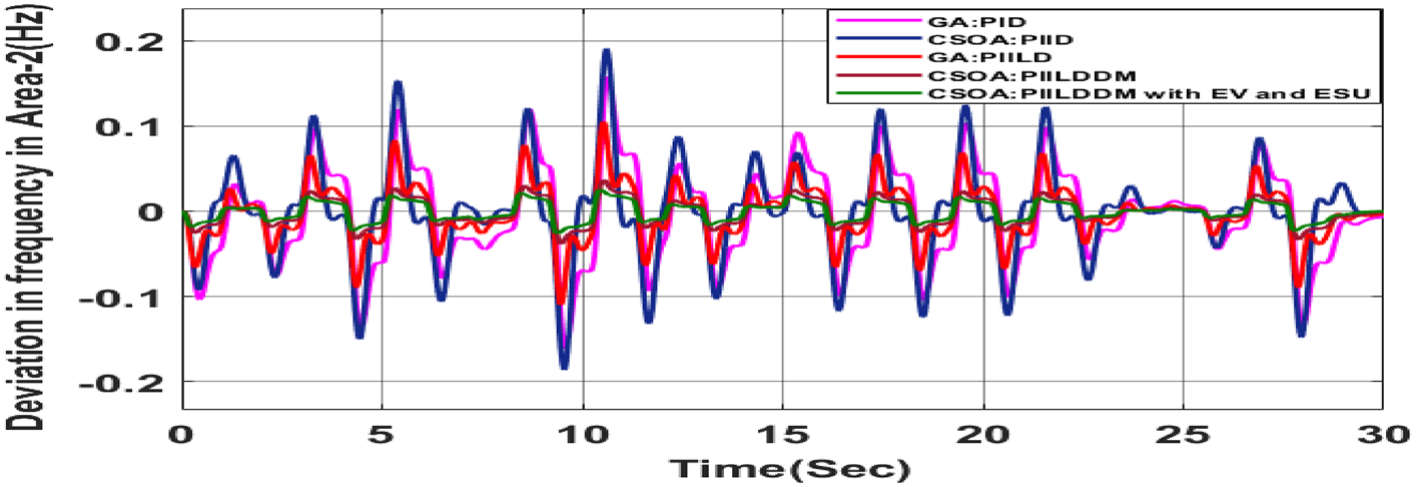
∆F_2_ reactions for CV with RLD.

**Fig. 64:**
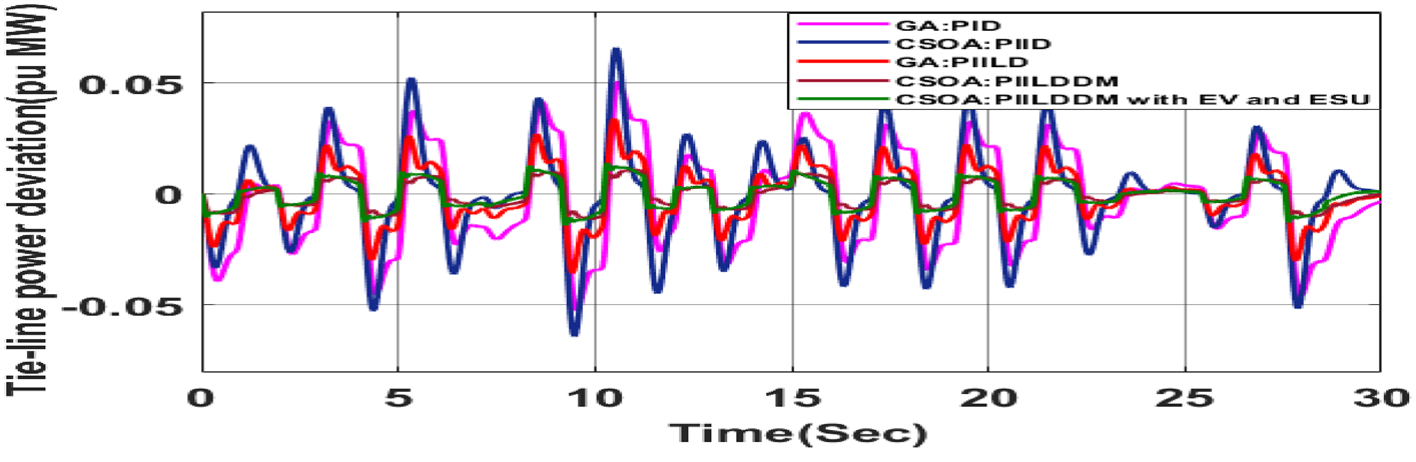
∆P_tie_ reactions for CV with RLD.

In this study, CSOA-PII^ʎ^DD^µ^ controller with hybrid ESS is tested for the restructured T-G-S system. The convergence characteristics for the same controller is shown in Fig. 52. It’s best ISTSE value is 3.16 × 10^–5^. The controller’s optimal gains are shown in Tables 6, 7. The Figs. 53, 54, 55, 56, 57, 58, 59, 60 and 61 display the dynamic reactions for numerous power transactions. It has confirmed that CSOA: PII^ʎ^DD^µ^ controller with hybrid ESS is effective. The further study investigates the CSOA- PII^ʎ^DD^µ^ controller’s resilience to load demand uncertainty for CV by imposing considerable random load demand for 30 s. Figures 62, 63 and 64 display the dynamic reactions for reorganised T-G-S system. The simulation study shows that overall performance utilizing the CSOA-PII^ʎ^DD^µ^ with EV + ESU control scheme is better in terms of dynamic specifications as compared to control schemes like CSOA-PII^ʎ^DD^µ^, CSOA-PII^ʎ^D, CSOA-PID, and GA-PID.

**Table 6:** Area 1 controllers optimal gains (T-G-S).

Sl. No	Controller	Area-1 subjected to 10% SLP						
		K_p1_	K_i1_	K_i11_	K_d1_	K_d11_	ʎ_1_	µ_1_
1	GA:PID	4.9983	4.9993	….	1.5462	…	…	…
2	CSOA:PID	3.012	3.004	….	2.998	…	…	…
3	CSOA:PIILD	10.013	9.9437	1.4951	2.222	…	− 0.1	…
4	CSOA:PIILDDM	10.012	9.9192	9.4167	6.2415	10.011	− 0.95	0.1
5	CSOA:PIILDDM with EV and ESU	10.111	9.9192	9.4167	6.2415	10.141	− 0.96	0.1

**Table 7:** Area 2 controllers optimal gains (T-G-S).

Sl. No	Controller	Area-2 subjected to 10% SLP						
		K_p2_	K_i2_	K_i22_	K_d2_	K_d22_	ʎ_2_	µ_2_
1	GA:PID	4.9969	3.7001	…	2.8817	…	….	…
2	CSOA:PID	0.01	0.7595	…	0.01	…	….	…
3	CSOA:PIILD	0.01	10.001	10.301	4.5708	…	− 0.1	…
4	CSOA:PIILDDM	5.3119	0.074	9.9446	1.8943	0.521	− 0.91	0.523
5	CSOA:PIILDDM with EV and ESU	5.3119	0.074	9.9446	1.8943	0.521	− 0.657	0.992

The dynamic responses of CSOA based FOPID + PFC with hybrid ESS is compared with CSOA-PII^ʎ^DD^µ^ controller considering hybrid ESS as shown in Tables 4 and 8. From the comparison it is clear that the improvement in frequency responses for area 1 and area 2 is 15.59% and 21.83%, respectively, and 27.16% improvement in the tie-line power deviation under the contract violation for the proposed T-G-S system.

**Table8:** System dynamic specs with CSOA:PIILDDM with EV and ESU.

Controller	Parameter	Overshoot (O_sh_)	Undershoot (U_sh_)	Settling Time (T_s_)	O_sh_	U_sh_	T_s_	Os_h_	U_sh_	T_s_
		**PT**			**BT**			**CV**		
GA:PID	∆ F_1_	0.002	− 0.0640	3.646	0.0025	− 0.0636	4.529	0.0027	− 0.0637	4.767
	∆ F_2_	0	− 0.0214	5.284	0	− 0.02	4.476	0	− 0.0197	4.535
	∆P_tie_	0.0002	− 0.0078	7.01	0.0002	− 0.007	6.873	0.00019	− 0.0079	9.215
CSOA:PID	∆ F_1_	0.0244	− 0.0594	2.773	0.025	− 0.0595	2.088	0.0252	− 0.05934	2.346
	∆ F_2_	0.0021	− 0.0192	3.058	0.0031	− 0.0177	2.063	0.0034	− 0.0174	3.738
	∆P_tie_	0.0004	− 0.0066	3.326	0.0001	− 0.006	3.356	0.00011	− 0.0067	4.806
CSOA:PIILD	∆ F_1_	0.0126	− 0.0503	2.178	0.0131	− 0.0503	2.082	0.0129	− 0.0504	2.049
	∆ F_2_	0	− 0.0127	3.964	0	− 0.0129	2.694	0	− 0.0127	3.688
	∆P_tie_	0	− 0.0047	3.081	0	− 0.004	3.169	0	− 0.0048	3.62
CSOA:PIILDDM	∆ F_1_	0.0044	− 0.0287	1.442	0.0044	− 0.0287	1.969	0.0046	− 0.0287	2.009
	∆ F_2_	0	− 0.0052	3.314	0	− 0.0038	2.438	0	− 0.0034	3.127
	∆P_tie_	0	− 0.0017	2.738	0	− 0.0019	2.856	0	− 0.0019	3.463
CSOA:PIILDDM with EV and ESU	∆ F_1_	0.0029	− 0.0174	1.424	0.0047	− 0.0174	1.364	0.0047	− 0.0174	1.334
	∆ F_2_	0	− 0.0021	3.127	0	− 0.0027	2.388	0	− 0.0026	2.556
	∆P_tie_	0	− 0.0011	2.317	0	− 0.002	2.171	0	− 0.0020	3.008

### The performance of CSOA: (1 + FOID) + PFC (gbellmf) controller

**Fig. 65:**
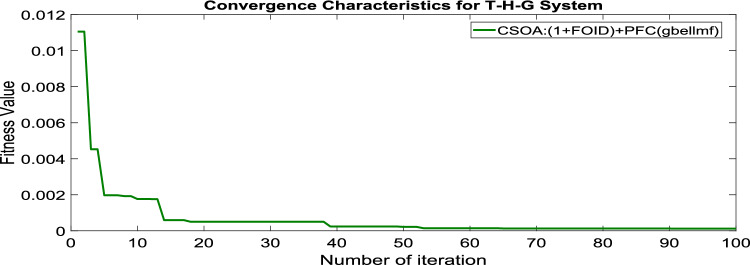
Convergence characteristics with CSOA: (1 + FOID) + PFC (gbellmf).

MATLAB/SIMULINK was used to simulate the system, GA, SOA, and CSOA are written in m files. For SOA, search agents (n) should be 6, population size (100), issue dimension (6) and maximum iterations (N) should be 100. The parameters considered for tuning of controllers using CSOA are shown in Table 9. The factors that influence area involvement are as follows: apf_1_ = 0.6; apf_2_ = apf_3_ = 0.2. This section examines how the restructured T-H-G system performs in respect to certain uncertainties, including 3% SLP in the both zones. The convergence characteristics for T-H-G system with CSOA: hybrid (1 + I^λ^D^µ^) and PFC (gbellmf) is shown in Fig. 65. The best ISTSE value is 1.1785e-04.The effectiveness of CSOA: hybrid (1 + I^λ^D^µ^) and PFC (gbellmf) is investigated for reorganized thermal-hydro-gas system, considering numerous electricity transactions. Its dynamic reactions are compared with other controllers such as GA: 1 + PFC (gbellmf), SOA: FOPID, SOA: PID, GA: PID, and OARs with various interconnections that include ∆P_dc_ in conjunction with the turbine controller as an extra control element.

**Table 9:** Area 1 controllers optimal gains(T-H-G).

Sl. No	Controller	Area-1 subjected to 3% SLP						
		K_p1_	K_i1_	K_d1_	ʎ_1_	µ_1_	K1	K2
1	GA:PID	0.788	0.987	0.948	**……**	**……**	**……**	**……**
2	SOA:PID	1.2918	1.7649	1.6837	**……**	**……**	**……**	**……**
3	SOA:FOPID	3.010	2.72891	2.7717	− 0.89	0.99	……	……
4	GA:1 + PFC(gbellmf)	**……**	**……**	**……**	**……**	**……**	0.0107	40.1416
5	CSOA:(1 + FOID) + PFC(gbellmf)	**……**	4.0763	1.7983	− 0.8523	0.9801	8.2793	37.9427

#### Poolco transaction (PT)

**Fig. 66:**
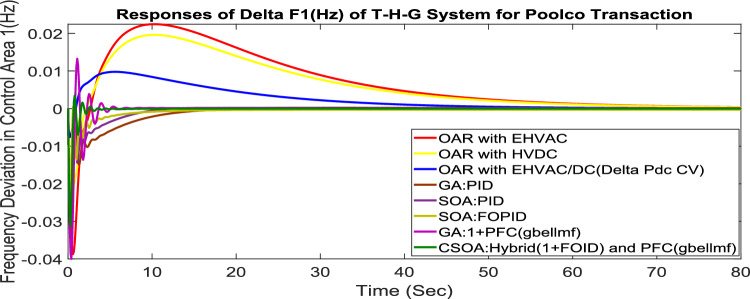
∆F_1_ reactions for PT.

**Fig. 67:**
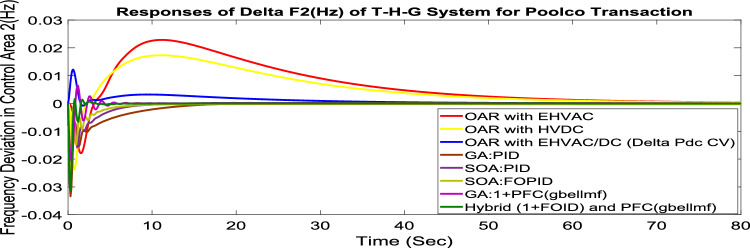
∆F_2_ reactions for PT.

**Fig. 68:**
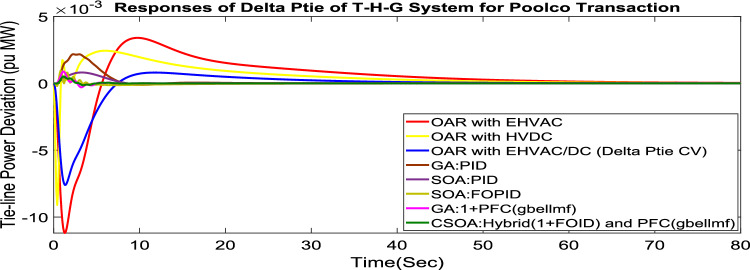
∆P_tie_ reactions for PT.

Under this arrangement, each GENCO is allowed to contribute in AGC according to their apfs, and each DISCO has contract with the GENCOs of same zone. As per the transaction policy, area 1 DISCOs cannot buy electricity from the second area GENCOs. All DISCOs and GENCOs have their contract as per DPM as given in (43). Due to area 2 DISCOs’ independence from area 1 or 2 GENCOs, the other DPM elements are nil. The optimal gains area 1 and area 2 controllers are depicted in Tables 8 and 9 respectively. The optimal gains with OARs considering various interconnections that include ∆P_dc_ as an additional control variable with the turbine controller are shown in Table 10. The computed value of delta P_tie_ is 0 pu MW, which matches the simulated result for the system with an EHVAC tie line, an HVDC tie line, and Parallel EHVAC/HVDC tie lines. Figures 66, 67 and 68 illustrate dynamic reactions for the proposed system subjected to 3% SLP in each area under various control schemes. It can be concluded that CSOA: hybrid (1 + I^λ^D^µ^) and PFC (gbellmf) rapidly settling the oscillations due to SLP. Hence the dynamic reactions obtained from CSOA: hybrid (1 + I^λ^D^µ^) and PFC (gbellmf) for the proposed system is found superior in terms of the ISTSE, maximum undershoot, peak overshoot, and settling time, as compared to GA: 1 + PFC (gbellmf), SOA: FOPID, SOA: PID, GA: PID, and OARs with various interconnections that include ∆P_dc_ along with the turbine actuator as another control element.

**Table 10:** Area 2 controllers optimal gains(T-H-G).

Sl. No	Controller	Area-2 subjected to 3% SLP						
		K_p2_	K_i2_	K_d2_	ʎ_2_	µ_2_	K3	K4
1	GA:PID	0.962	0.993	0.995	**……**	**……**	**……**	**……**
2	SOA:PID	1.2770	1.5695	1.6846	……	……	……	……
3	SOA:FOPID	2.8902	3.001	2.9989	− 0.97	0.999	……	……
4	GA:1 + PFC(gbellmf)	**……**	**……**	**……**	**……**	**……**	0.0142	42.9512
5	CSOA:(1 + FOID) + PFC(gbellmf)	**……**	3.4973	1.9411	− 0.7955	0.9712	8.4011	47.6518

#### Bilateral transaction (BT)

**Fig. 69:**
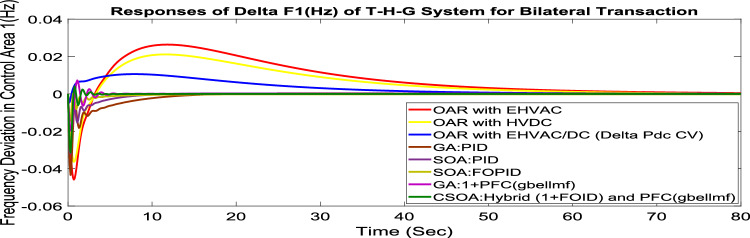
∆F_1_ reactions for BT.

**Fig. 70:**
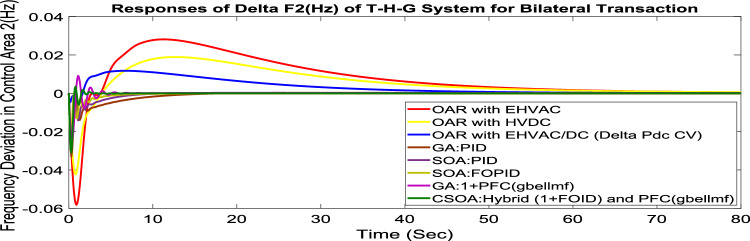
∆F_2_ reactions for BT.

**Fig. 71:**
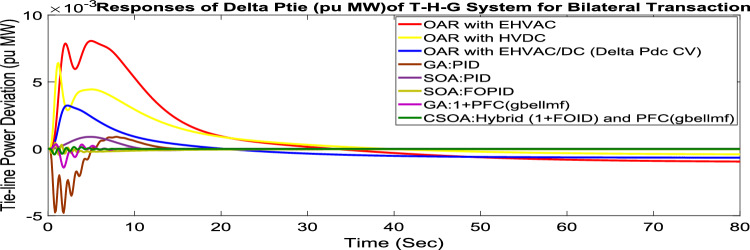
∆P_tie_ reactions for BT.

The BT includes the contract of transactions between a GENCO and a DISCO in the same area and any other location. According to Eq. (44), each DISCO and GENCO in the system constitutes its own contract as the DPM i.e. this agreement allows energy distribution businesses in a certain sector to negotiate with any zone’s GENCOs , whether they are nearby or not. Both parties accept contract terms. DISCO-4 does not need power from GENCO1. Hence the DPM contract participation factor is 0. Each GENCO assigns pu MW to each apf as described earlier. The optimal gains area 1 and area 2 controllers are depicted in Table 8 and 9 respectively. The optimal gains with OARs considering various interconnections that include ∆P_dc_ as an additional control variable with the turbine controller are shown in Table 10. The scheduled value of delta P _tie_ is described by Eq. (36). The computed value of delta P_tie_ are − 0.0009503 pu MW,− 0.0004099 pu MW, and − 0.0006614 pu MW at 80 s which matches the simulated result for the system with an EHVAC tie line, an HVDC tie line, and Parallel EHVAC/HVDC tie lines. For other controllers, delta P_tie_ are 0pu MW. Figures 69, 70 and 71 illustrate dynamic reactions for the proposed system subjected to 3% SLP in each area under various control schemes. It can be concluded that CSOA: hybrid (1 + I^λ^D^µ^) and PFC (gbellmf) rapidly settling the oscillations due to SLP. Hence the dynamic reactions obtained from CSOA: hybrid (1 + I^λ^D^µ^) and PFC (gbellmf) for the proposed system is found superior in terms of the ISTSE, maximum undershoot, peak overshoot, and settling time, as compared to GA: 1 + PFC (gbellmf), SOA: FOPID, SOA: PID, GA: PID, and OARs with various interconnections that include ∆P_dc_ as an another control element.

#### Contract violation (CV)

**Fig. 72:**
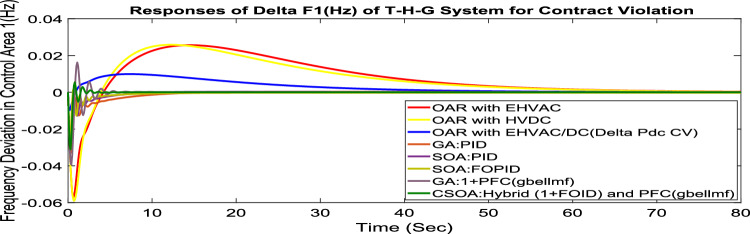
∆F_1_ reactions for CV.

**Fig. 73:**
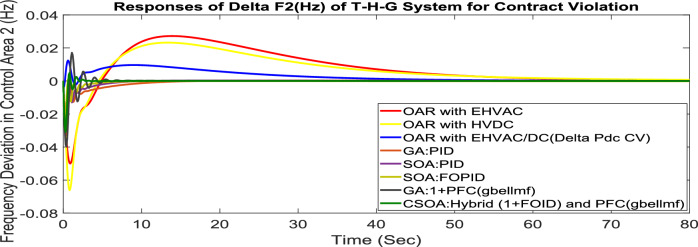
∆F_2_ reactions for CV.

**Fig. 74:**
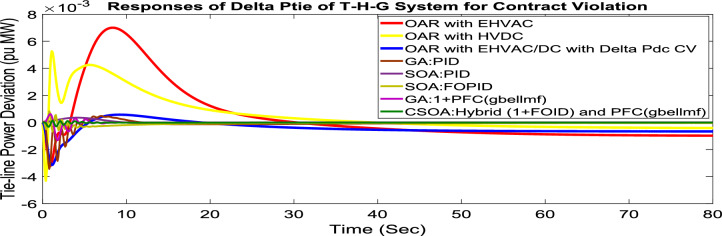
∆P_tie_ reactions for CV.

A DISCO may sometimes violate the terms of the contract arrangement when things are terrible. In this scenario, GENCOs could need to provide the DISCO with more power than what is specified in the contract. The GENCOs belonging to each area shall fulfill the DISCO’s request for un-contracted extra power. Consider a contact violation scenario where DISCO-1 requested 0.005 p.u. MW of un-contracted electricity from the local GENCOs and DISCO-3 requested 0.005 p.u. MW of un-contracted electricity from the local GENCOs. Due to the increased need for electricity, area 1’s total load request has raised to 0.015 p.u. MW, while new power requests in the second sector indicate 0.015 pu MW. The optimal gains with OARs considering various interconnections that include ∆P_dc_ as an additional control variable with the turbine controller are shown in Table 10. The scheduled value of delta P _tie_ is described by Eq. (36). The computed value of delta P_tie_ are − 0.000967 pu MW,− 0.0005117 pu MW, and − 0.000665 pu MW at 80 s which matches the simulated result for the system with an EHVAC tie line, an HVDC tie line, and Parallel EHVAC/HVDC tie lines. For other controllers, delta P_tie_ are 0pu MW. Figures 72, 73 and 74 illustrate dynamic reactions for the proposed system subjected to 3% SLP in each area under various control schemes. It can be concluded that CSOA: hybrid (1 + I^λ^D^µ^) and PFC (gbellmf) rapidly settling the oscillations due to SLP. Hence the dynamic reactions obtained from CSOA: hybrid (1 + I^λ^D^µ^) and PFC (gbellmf) for the proposed system is found superior in terms of the ISTSE, maximum undershoot, peak overshoot, and settling time, as compared to GA: 1 + PFC (gbellmf), SOA: FOPID, SOA: PID, GA: PID, and OARs with various interconnections that include additional ∆P_dc_ (Table 11).

**Table 11:** The optimal feedback gain matrices for the T-H-G system with OARs.

EHVAC	0.4837	0.6864	21.6671	− 5.0190	0.5680	3.0981	6.3466	− 6.7353	1.5505	1.8577	1.6270	0.1133	0.8221
	− 0.1140	0.0212	0.5410	0.7636	− 1.1746	0.3668	0.1824	0.2563	− 0.1281	− 4.0062	− 0.5362	− 0.8441	
	0.0571	0.7845	6.7963	− 1.7844	0.0628	0.0455	0.1396	− 1.6534	0.0058	− 0.0025	− 0.1191	− 0.0008	5.2744
	− 0.8793	0.4206	1.9232	3.9186	− 11.7290	1.1103	1.2476	1.7111	− 0.6912	1.1423	− 0.8441	0.5362	
HVDC	0.1053	0.9278	19.7452	− 4.7585	0.5043	1.8192	4.0971	− 5.2050	0.8419	1.1802	1.2534	0.1028	2.3259
	− 0.3020	0.0785	1.5987	2.8199	− 1.7564	0.9940	0.9129	0.6861	− 0.2740	0.2190	− 0.6671	− 0.745	
	0.3858	0.5892	6.9812	− 1.6856	0.0899	0.8378	1.5640	− 2.4210	0.4366	0.4034	0.1454	0.0054	4.3986
	− 0.7583	0.3910	1.2994	2.6401	− 11.5254	0.7754	0.8335	1.3764	− 0.5572	0.0228	− 0.745	0.6671	
Parallel EHVAC/DC tie− lines (Delta Pdc as CV)	− 0.1808	− 0.2562	11.5030	− 2.6766	0.4158	1.2492	2.8461	− 2.4561	0.5894	0.9137	1.1280	0.0981	− 1.5989
	0.2374	− 0.0549	− 1.0523	− 2.2053	2.1438	− 0.5608	− 0.6878	− 0.6597	0.2808	− 2.7708	− 0.0012	− 0.8563	
	− 0.2654	0.2064	5.9918	− 1.7148	0.0348	− 0.8364	− 1.6536	− 0.7165	− 0.418	− 0.4255	− 0.4494	− 0.0069	2.7251
	− 0.4001	0.3636	0.7150	1.4750	− 6.0482	0.4527	0.5414	0.7912	− 0.2581	0.9172	− 0.9991	0.0235	
	1.4454	0.5795	4.7327	− 1.0964	0.0652	0.3413	− 0.5042	− 0.7619	0.4311	− 0.1440	− 0.9833	− 0.0292	1.3399
	− 0.3287	− 0.0236	0.4207	0.7562	− 3.6673	0.2298	0.1602	0.2429	− 0.1612	0.1217	0.0435	0.516	

The proposed AGC regulator with a hybrid ESS enhances frequency control quality, which in turn reduces penalties associated with contract deviations in restructured markets. This is one of the reasons why the suggested regulator is economically viable. By synchronizing the dynamics of rapid and slow storage, the framework reduces the amount of wear and tear on traditional governors and BESS, which ultimately results in a longer asset lifespan and decreased operational and maintenance expenses. In addition, improved regulatory performance enables utilities and GENCOs to participate in ancillary service markets (frequency regulation, spinning reserve), presenting a potential income stream for these entities.

The complexity of the implementation: An additional supervisory optimization loop (CSOA-tuned regulator) and a hybrid ESS control block have been added to the AGC layer, which is the only component of the control structure that has not been modified. The tuning based on CSOA is carried out offline, meaning the online AGC operation only requires executing the tuned regulator equations. This does not result in an increase in the amount of computing load at the control center.

Real-World Deployment Considerations: The technique may be incorporated in stages: the existing AGC can be left in its current state while the ESS control layer is introduced to share the work of regulation. There is an explicit inclusion of practical limitations, such as SoC limits, ramp-rate restrictions, governor dead-band, and GRC, which brings the findings closer to where they would be in the field.

## Risks to the validity of the findings

Although the proposed AGC framework with hybrid ESS demonstrates improved dynamic performance in MATLAB/Simulink, it is important to recognize potential challenges in validating simulation results against real-world conditions. These challenges can arise from both internal modelling aspects and external system uncertainties:

### Internal challenges

Model accuracy: Any simplification or inaccuracy in the turbine-governor, hybrid ESS, or market participation models may lead to deviations from real system behavior.

Solver precision: The accuracy of numerical solvers (e.g., ode45, ode23tb) and integration step size directly affects the fidelity of dynamic responses.

Input data updates: Outdated or approximated parameters (e.g., governor time constants, market demand profiles) may reduce reliability. Periodic updating of input datasets using real utility data is essential for maintaining simulation validity.

### External challenges

Market and regulatory dynamics: Deregulated environments are influenced by evolving pricing strategies, bilateral contracts, and market rules. If these are not modelled accurately, the control strategy may not reflect practical outcomes.

Operational processes and equipment constraints: Communication bandwidth limitations, grid topology changes, or unforeseen equipment failures can alter system dynamics compared to simulations.

Data availability: Validation is constrained by limited access to real-world operational data (both historical and real-time). Without adequate datasets, benchmarking simulation results against actual system behavior remains difficult.

### Mitigation strategies

To improve realism and reduce the simulation–reality gap: We have selected validated system models from literature and ensured parameter consistency with standard AGC test systems. Solver settings were carefully chosen for accuracy/stability trade-offs. Constraints such as governor dead-band, GRC, and ESS SoC limits were included to approximate real hardware behaviour.

## Conclusion

In this research work, the optimal AGC regulators like FOPID + PFC with hybrid ESS, PII^ʎ^DD^µ^ with hybrid ESS, and combined (1 + FOID) and polar fuzzy controllers are proposed for restructured power systems. The tuning the controller’s parameters is accomplished through CSOA. Based on the findings of the sensitivity analysis, it has been determined that the CSOA-based FOPID + PFC is sufficiently resilient and robust under random load demand, parameter uncertainties, and communication time delay. In comparison to the CSOA-optimized PII^ʎ^DD^µ^ controller, a CSOA-optimized FOPID + PFC utilizing hybrid ESS for the proposed system can improve frequency responses for Area-1 and Area-2 by 15.59% and 21.83%, respectively, and 27.16% improvement in the tie-line power deviation under contract violation.

Another study reveals the efficacy of a CSOA-based combined (1 + FOID) controller and PFC for restructured T-H-G power system considering numerous electricity transactions. This unique AGC regulator was found superior in terms of the ISTSE, maximum undershoots, peak overshoot, and settling time, as compared to other controllers such as GA: 1 + PFC (gbellmf), SOA: FOPID, SOA: PID, GA: PID, and OARs with EHVAC/ DC links that include ∆P_dc_ as an additional control variable with the turbine controller. The comparative study reveals that the outcomes are strong and encouraging in achieving AGC’s goals.

Therefore, in terms of frequency stability, tie-line power regulation, and control effort, these suggested AGC regulators performed better than traditional methods. In the hybrid ESS, batteries balanced long-term dynamics while the ESU decreased short-term oscillations. In restructured situations, enhanced AGC performance permits auxiliary service market participation and reduces deviation penalties. By reducing the wear on generators and storage units, the hybrid ESS prolongs equipment life and lowers operating costs. By ensuring contract compliance and robust tie-line power control in the face of uncertainty and interruptions, the proposed method enhances the interaction between GENCOs and DISCos. Despite parameter swings, random load demand, and communication time delays, robustness assessments demonstrate that the ideal AGC regulator can function in real-time, given the existing infrastructure. Pilot testing and HIL validation are necessary for field applicability. Future studies will extend the proposed method to hardware-in-the-loop (HIL) or RTDS real-time implementation, cyber-security-aware AGC, and multi-objective frameworks.

## Supplementary Information

Supplementary Information.

## Data Availability

The datasets used and/or analysed during the current study available from the first author on reasonable request.

## Abbreviations

ACE: Area control error
ACO: Ant colony optimization
AEO: Artificial ecosystem optimization
AGC: Automatic generation control
BESS: Battery energy storage system
COC-FOD: Cascade optimal controller-fractional order derivative
CSOA: Cuckoo search optimization algorithm
CTD: Communication time delay
DPM: Disco participation matrix
DISCOs: Distribution companies
ESS: Energy storage system
ESU: Energy storage unit
EV: Electric vehicle
FF: Fitness function
FLC: Fuzzy logic controller
FOPID: Fractional-order PID
FOOA: Fifth-order oustaloup’s approximation
GA: Genetic algorithm
GENCOs: Generation companies
GNA: Global neighborhood algorithm
GOA: Grasshopper optimization algorithm
GRC: Generation rate constraint
GWO: Grey wolf optimization
HES: Hydrogen energy storage
IACCO: Integral of absolute change in controller output
IOPID: Integer order PID
ISOs: Independent system operators
ISTSE: Integral of squared time multiplied by squared error
N-G-S: Nuclear gas solar
PFC: Polar fuzzy controller
PID: Proportional integral derivative
RES: Renewable energy sources
RFB: Redox flow batteries
RPS: Restructured power system
RLD: Random load demand
SCA: Sine cosine algorithm
SOA: Skill optimization algorithm
T-G-S: Thermal gas solar
T-H-G: Thermal hydro gas
TLBO: Teaching learning-based optimization
TD: Tilt-derivative
TF: Transfer function
TGS: Thermal-gas-solar
THG: Thermal-hydro-gas
TID: Tilt-integral- derivative
TRANSCOs: Transmission companies
UC: Ultra-capacitor

## References

[1] Sharma, D. Load frequency control: a literature review. *Int. J. Sci. Technol. Res.* **9**, 6421–6437 (2020).

[2] Mishra, R. N., Chaturvedi, D. K. & Kumar, P. Recent Philosophies of AGC Techniques in Deregulated Power Environment. *J. Inst. Eng. India Ser. B*10.1007/s40031-020-00463-8 (2020).

[3] Singh, B., Slowik, A. & Bishnoi, S. K. Review on soft computing-based controllers for frequency regulation of diverse traditional, hybrid, and future power systems. *Energies* **2023**, 16. 10.3390/en16041917 (1917).

[4] Oustaloup, A. From fractality to non-integer derivation through recursivity, a property common to these two concepts: A fundamental idea from a new process control strategy. *In Proceedings of the 12th IMACS World Congress*, (Paris, France, 18–22 July 1998). [Google Scholar]

[5] Jain, S. & Hote, Y. Design of fractional PID for Load frequency control via int ernal model control and Big bang Big crunch optimization. *IFAC-Pap.* **51**, 610–615 (2018).

[6] Kumar, N., Tyagi, B. & Kumar, V. Application of fractional order PID controller for AGC under deregulated environment. *Int. J. Autom. Comput.* **15**, 84–93 (2018).

[7] Guha, D., Roy, P. K. & Banerjee, S. Grasshopper optimization algorithm scaled fractional order PI-D controller applied to reduced order model of load frequency control system. *Int. J. Model. Simul.* **40**, 217–242 (2020).

[8] Kumar, K. V. & Ganesh, V. Design of fractional order proportional integral controller for load frequency control of multi-area power system under deregulated environment. *Int. J. Power Energy Convers.* **11**, 223–247 (2020).

[9] Saxena, S. Load frequency control strategy via fractional-order controller and reduced-order modeling. *Int. J. Electr. Power Energy Syst.* **104**, 603–614 (2019).

[10] Debbarma, S. & Dutta, A. Utilizing Electric Vehicles for LFC in Restructured Power Systems Using Fractional Order Controller. *IEEE Trans. Smart Grid* **8**, 2554–2564 (2017).

[11] Nithilasaravanan, K., Thakwani, N., Mishra, P., Kumar, V. & Rana, K. P. S. Efficient control of integrated power system using self-tuned fractional-order fuzzy PID controller. *Neural Comput. Appl.* **31**, 4137–4155 (2019).

[12] Wang, H. et al. Design of a fractional order frequency PID controller for an islanded Microgrid: a multi-objective extremal optimization method. *Energies* **10**, 1502 (2017).

[13] Al-Mayyahi, A., Aldair, A. A. & Chatwin, C. Control of a 3-RRR Planar Parallel Robot Using Fractional Order PID Controller. *Int. J. Autom. Comput.* **17**, 822–836 (2020).

[14] Zhang, J. et al. Workspace analysis and motion control strategy of robotic mine anchor drilling truck manipulator based on the WOA-FOPID algorithm. *Front. Earth Sci.* **10**, 1253 (2022).

[15] Ataşlar-Ayyıldız, B. Robust Trajectory Tracking Control for Serial Robotic Manipulators Using Fractional Order-Based PTID Controller. *Fractal Fract* **7**, 250. 10.3390/fractalfract7030250 (2023).

[16] Gnaneshwar, K., Padhy, P.K. Robust design of tilted integral derivative controller for non-integer order processes with time delay.* IETE J. Res*. (2021)

[17] Bhuyan, M., Das, D. C., Barik, A. K.; Sahoo, S. C. Performance assessment of novel solar thermal-based dual hybrid microgrid system using CBOA optimized cascaded PI-TID controller. *IETE J. Res*. (2022)

[18] Sharma, M., Prakash, S., Saxena, S. & Dhundhara, S. Optimal fractional-order tilted-integral-derivative controller for frequency stabilization in hybrid power system using salp swarm algorithm. *Electr. Power Compon. Syst.* **48**, 1912–1931 (2021).

[19] Sharma, M., Prakash, S., Saxena, S. Robust load frequency control using fractional-order TID-PD approach via salp swarm algorithm. *IETE J. Res.* (2021)

[20] Lu, C., Tang, R., Chen, Y. Q. & Li, C. Robust tilt-integral-derivative controller synthesis for first-order plus time delay and higher-order systems. *Int. J. Robust Nonlinear Control* **33**, 1566–1592 (2023).

[21] Mohamed, E. A. et al. An Optimized Hybrid Fractional Order Controller for Frequency Regulation in Multi-Area Power Systems. *IEEE Access* **8**, 213899–213915 (2020).

[22] Ahmed, E. M., Mohamed, E. A., Elmelegi, A., Aly, M. & Elbaksawi, O. Optimum modified fractional order controller for future electric vehicles and renewable energy-based interconnected power systems. *IEEE Access* **9**, 29993–30010 (2021).

[23] Choudhary, R., Rai, J. N. & Arya, Y. Cascade FOPI-FOPTID controller with energy storage devices for AGC performance advancement of electric power systems. *Sustain. Energy Technol. Assess.* **53**, 102671 (2022).

[24] Yanmaz, K., Mengi, O. O., Sahin, E. Advanced STATCOM Control with the Optimized FOPTID-MPC Controller. *IETE J. Res.* (2022)

[25] Umrao. R., Chaturvedi D. K. (2010). Load frequency control using polar fuzzy controller, *TENCON 2010 - IEEE Region 10 Conference,* 557–562, (Fukuoka, Japan, 2010), 10.1109/TENCON.2010.5686740.

[26] Chaturvedi, D. K., Umrao, R. & Malik, O. P. Adaptive polar fuzzy logic-based load frequency controller. *Electr Power Energy Syst* **66**, 154–159 (2015).

[27] Lotfy, M. E., Senjyu, T., Abdel-Fattah Farahat, M., Abdel-Gawad, A. F. & Matayoshi, H. A PFC Scheme for Hybrid Power System Using V2G Technique. *Energies* **10**(8), 1083 (2017).

[28] Mishra, R. N., Kumar, N., Zuhaib, M. (2023), AGC of 2-Area Restructured Power System using Polar Fuzzy Controller and Energy Storage Units, *2nd International Conference for Innovation in Technology (INOCON)*, pp. 1-6, (Bangalore, India, 2023), 10.1109/INOCON57975.2023.10101346

[29] Mishra, R. N. & Kumar, N. An energy storage system with SOA-based FONPID controller for AGC study in restructured power systems. *Electr Eng*10.1007/s00202-024-02795-w (2024).

[30] Rangi, S., Jain, S. & Arya, Y. Utilization and performance comparison of several HESSs with cascade optimal-FOD controller for multi-area multi-source power system under deregulated environment. *J. Energy Storage* **94**, 112469. 10.1016/j.est.2024.112469 (2024).

[31] Mishra, R. N., Kumar, N. & Chaturvedi, D. K. Design and analysis of optimal AGC regulator for multi-area power systems with TCPS and energy storage unit in deregulated environment. *Opt. Cont. Appl. Meth. (Wiley)* **45**(4), 1456–1502 (2024).

[32] Yang, X.-S. & Deb, S. Multiobjective cuckoo search for design optimization’. *Comput. Oper. Res.* **40**(6), 1616–1624 (2013).

[33] Yang, X., Deb, S. Cuckoo Search via Lévy flights’, In *2009 World Congress on Nature & Biologically Inspired Computing (NaBIC)*, 10.1109/NABIC.2009.5393690.2009.

[34] Givi, H. (2022). Skill Optimization Algorithm (SOA) (https://www.mathworks.com/matlabcentral/fileexchange/110675-skill-optimization-algorithm-soa), MATLAB Central File Exchange.

[35] Givi, H. & Hubalovska, M. Skill optimization algorithm: a new human-based metaheuristic technique. *Comput. Mater. Continua* **74**(1), 179–202 (2023).

[36] Tabassum. N., Jahan, E., Goswami, N., Rahman Zishan M. S. Analysis of Genetic Algorithm-Optimized PID Controllers for a Two-Area Automatic Generation Control System, *10th IEEE ICPS,* pp. 1-6 (Cox’s Bazar, Bangladesh, 2023), 10.1109/ICPS60393.2023.10428703

[37] Kalyan, C. H. N. S. & Rao, G. S. Impact of communication time delays on combined LFC and AVR of a multi-area hybrid system with IPFC-RFBs coordinated control strategy. *Prot. Control. Mod. Power Syst.* **6**, 7. 10.1186/s41601-021-00185-z (2021).

[38] Reddy, P. V. S. Fuzzy Logic Based on Belief and Disbelief Membership Functions. *Fuzzy Inf. Eng.* **9**(4), 405–422. 10.1016/j.fiae.2017.12.001 (2017).

