# Supplementary material for: Optimal AGC regulators for power systems under restructured configuration with hybrid ESS
Source: Sci Rep. 2025 Oct 14;15:35926. doi: 10.1038/s41598-025-19710-3 (PMC12521574; doi:10.1038/s41598-025-19710-3)
Supplement: Supplementary file 1 — Supplementary Information. [file 41598_2025_19710_MOESM1_ESM.docx]

**Appendix A**

With a turbine controller and ∆P_dc_ as another control element, the state domain model patterns for the two-area restructured T-H-G system with parallel interties are, in brief, as follows:

1. Elements of the system matrix that are not zero [26x26]:

,,,,,,, ,,,,,,,,,,,,,,,,,,,

,,,,,,,,,,,,,,,,,,,,,,,,,,,,,,,,,,,,,,,,,,,,,,,,,,

2.Non-zero elements of control matrix [26x3]:

,,,,,,

,,,, ,,,, ,,,,

3.Non-zero elements of disturbance matrix [26x6]:

, , , , , , , , , , ,,,,,, , , ,,,,,,,,,,,,,,,,,,,,,,,,,,,,,,,,,,,,,,,,,,,,,,,,,,,

As identity matrices, the output (C), state cost weighting (Q), and control cost weighting (R) matrices were chosen for this investigation. These matrices' dimensions are [26 x 26], [26 x 26], and [3 x 3], respectively.

**Appendix B**

The system being analysed possesses the subsequent nominal parameters [24, 30, 33].

| Parameters | Values |
| --- | --- |
| Capacity rating for every control area (P_ri_) | 2000 MW |
| Nominal frequency (f^0^) | 60 Hz |
| Base Power (P_base_) | 2000 MVA |
| Frequency bias constants(Bi) | 0.425 p. u MW/Hz |
| Regulation constants (R_1_=R_3_, R_2_=R_4_) | (0.417,0.1227) Hz/p.u. |
| Time constant of the steam turbine regulator (T_G_) | 0.06 s |
| The time constant for turbines that produce steam (T_t_) | 0.3 s |
| Electrical system’s gain constant : (K_PS1_, K _PS2_) | 120 Hz/p.u. MW |
| The electricity system's time constant : (T_P1_,T _P2_) | 20 s |
| Coefficient of synchronization for AC links (T_12_) | 0.2712 |
| Coefficient of area size proportion (a_12_) | -1 |
| The electricity capacity of the tie-line (P_tie_) | 200 MW |
| Lead time constant for the gas turbine speed regulator: (X_G_) | 0.6 s |
| The lag time constant of the gas turbine speed regulator: (Y_G_) | 1.1 s |
| The valve positioner's gas turbine constant : (b _g_ ,c _g_) | 0.049 s,1 |
| Fuel time constant for gas turbines : (T _F_) | 0.239 s |
| Reaction time delay for gas turbine combustion (T_CR_) | 0.01 s |
| Discharge volume time constant for gas turbine compressors :(T_CD_) | 0.2 s |
| Gain of ultra-capacitor (K_UC_) | -0.7 |
| Time constant of ultra-capacitor (T_UC_) | 0.9 s |
| Gain of solar power (K_PV_) | 1 |
| Time constant of solar power (T_PV_) | 1.8 s |
| Dashpot time constant (T_R_) | 5s |
| Speed of a hydro turbine time constant for transient droop in the governor(T_RH_ ) | 28.75s |
| Time constant for the hydro governor (T_GH_ ) | 0.2s |
| Water starting time constant (T_W_) | -1s |
| Gain of electric vehicle (K_EV_) | 0.4 |
| Time constant of electric vehicle (T_EV_) | 0.8 s |
